# Toward Comprehensive Assessment of Beliefs and Attitudes Related to Physical Activity in Young Adults: Pilot Study

**DOI:** 10.2196/69094

**Published:** 2025-10-16

**Authors:** Theodorus D B Noordover, Aave Hannus, Kenn Konstabel

**Affiliations:** 1 Department of Psychology University of Tartu Tartu Estonia; 2 National Institute for Health Development Tallinn Estonia

**Keywords:** theory of planned behavior, physical activity, university students, questionnaire, acceptance and commitment therapy

## Abstract

**Background:**

Studies show that despite the positive effects of physical activity (PA), most university students are not active enough. For interventions, it is necessary to understand the determinants of PA behavior. Theory of planned behavior (TPB) is one of the most widely used frameworks to describe the psychological determinants of health behavior. Research has shown that in addition to the determinants included in TPB (attitudes, subjective norms, perceived control, and intention), fear of negative outcomes (eg, discomfort or pain) is a major barrier to increasing one’s PA. It has been shown that accepting the possibility of unpleasant outcomes may help in reaching one’s PA goals.

**Objective:**

The purpose of this study was to create a questionnaire of PA determinants based on TPB and complemented by the topic of acceptance of unpleasant outcomes. The questionnaire is thus meant for evaluating the effectiveness of health psychological PA interventions in university students and young adults.

**Methods:**

This study was carried out using qualitative and quantitative methods and consisted of three phases: (1) an elicitation study for item generation, (2) pretesting for clarity and understanding, and (3) item selection using conceptual and psychometric criteria (based on a pretest with N=447) to maximize domain coverage and avoid redundancy.

**Results:**

A questionnaire covering the core topics of TPB plus acceptance of negative outcomes was constructed, using a combination of qualitative and quantitative methods. The final shortened questionnaire consists of the following question blocks: positive and negative behavioral beliefs, acceptance of negative outcomes, subjective norms (injunctive and descriptive norms, and motivation to comply), and control beliefs. In terms of predicting self-reported PA, the shortened questionnaire was equal to the unabridged version. Notably, negative behavioral beliefs and acceptance of negative outcomes had opposite-signed correlations with self-reported PA (–0.22 and 0.32, *P<.*001). Despite the aim of avoiding redundancy, several item bundles (eg, positive and negative behavioral beliefs, and acceptance of negative outcomes) were highly homogenous in the final version, and are thus usable as psychometric scales.

**Conclusions:**

This questionnaire can assess a range of PA determinants and has good psychometric properties. The questionnaire can be used to assess the beliefs and attitudes (behavioral beliefs, perceived norms, control beliefs, and acceptance of negative consequences) related to PA in young adults when planning interventions, as well as evaluating the effects of health psychological interventions aiming to increase PA.

## Introduction

### Background

Physical inactivity is associated with several health risks, which increased physical activity (PA) can reduce [1]. In students, PA has been shown to relate to better overall (mental) health, happiness, and quality of life [2-4] as well as better academic performance [5].

Despite these benefits, many people, including university students, are not physically active enough. Depending on the criteria, the percentage of students not meeting the World Health Organization PA recommendations varies from 22% to 80% [3,5-7], and they spend an average of 7 to 10 hours per day being sedentary [8].

Increasing students’ PA can thus be beneficial for physical and mental health, as well as academic performance. For planning interventions, one needs systematic information about the determinants of PA, including attitudes, beliefs, perceived barriers, etc. A review of PA barriers in undergraduate students [9] suggested external barriers (eg, lack of time and facilities) are more prevalent than internal ones. The generality of this conclusion, however, is difficult to test: studies have used different methods (the method of qualitative studies is often not described; in quantitative studies, the choice of barriers to select from differs across studies); several barriers are not easily classifiable as internal versus external (eg, stress or work-related fatigue); the geographical variability in barriers is not systematically considered. A more recent review cited lack of time, motivation, and of accessible places as the most important barriers for high school and university students [10].

Notably, male students generally show higher levels of PA compared to female students [6,11], indicating the need for more tailored interventions.

### Attitudes and Beliefs as Determinants of PA

The theory of planned behavior (TPB) is a widely used framework for understanding health behavior and developing interventions [12,13]. The central component in the TPB is the intention to carry out a certain behavior. Intentions, in turn, are influenced by attitudes, subjective norms, and perceived behavioral control [14].

Attitudes refer to “the degree to which a person has a favourable or unfavourable evaluation or appraisal of the behaviour in question” [15]; subjective norms refer to “perceived social pressure to perform or not perform the behaviour” [15]. Perceived behavior control refers to “the perceived ease or difficulty of performing the behaviour” [15]. The more positive these 3 factors are, the stronger the intention to perform the specific behavior [15].

In TPB, attitudes are determined by behavioral beliefs and outcome evaluations [16]. Behavioral beliefs are related to the belief that carrying out the behavior in question is associated with certain results or consequences, while outcome evaluations refer to the valuation of these consequences [16]. For example, one may believe that engaging in PA leads to feeling good (behavioral belief), and this feeling is evaluated as positive (outcome evaluation).

Subjective norms are decomposable into normative beliefs (whether people in general, or some specific referents, approve or disapprove of a behavior) and motivation to comply. However, the actual behaviors of the referents (descriptive norm) may also be important. As a hypothetical example, an individual’s parents may approve their being physically active (normative beliefs), engage in PA themselves (descriptive norms), and the individual is somewhat motivated to align with their parents’ expectations (motivation to comply) [16].

Perceived control, also called personal agency, is subdivided into control beliefs and perceived power. Control beliefs relate to the likelihood of factors helping or hindering a behavior, while perceived power refers to how much these factors impact the ease or difficulty of performing the behavior [16]. As a hypothetical example, one may believe that one’s equipment for skiing is poor (strength of control belief) and that without good equipment, skiing is extremely hard (control belief power). Ajzen [17] argues that the concept of self-efficacy is fundamentally similar to that of control beliefs: self-efficacy and perceived controllability can be conceptualized as partially overlapping but distinct aspects of perceived behavioral control. However, self-efficacy can be thought of as a direct way to ask about one’s ability to perform a behavior, whereas the TPB framework offers an indirect measure (strength of control belief multiplied by the power of control belief). The indirect measure cannot be implemented for all beliefs: for example, one can easily ask about the perceived power of changeable factors (eg, how much does bad weather make it more difficult to be active), but the perceived strength of such factors (eg, how bad is the weather) can only be asked about a specific moment or situation.

While TPB has been criticized on different grounds [18], our main goal here is to avoid leaving out any concept that has important consequences on PA and is feasible to be asked in a self-report questionnaire.

In addition to the determinants included in TPB, fear of negative outcomes is found to be an important barrier to increasing PA in several studies. For example, students in Colombia [19] but not so much in the United States [20] see the threat of injuries as an important barrier to PA. Fatigue brought by exercise, as well as similar items, has been found to be an important barrier in several studies [21-23]. However, acceptance and commitment therapy (ACT) has shown promise as a framework for PA intervention [24]. ACT promotes psychological flexibility by encouraging present-moment awareness and acceptance of internal experiences and acting in line with personal values [25].

In ACT, acceptance means embracing one’s experiences without trying to change them [25]. That is, one may engage in a behavior even if one believes it is likely to lead to negative outcomes, provided that one is willing to accept these outcomes. In TPB, conversely, a likely negative outcome should directly lead to a decrease in the intention to perform the behavior.

### Current TPB Questionnaires: What Is Missing

Excluding the questionnaires targeting specific health conditions (eg, [26]), there are not many TPB-based PA questionnaires available; two could be applicable in a general population of university students: (1) a questionnaire based on extended TPB by Cheng et al [27] comprising subscales on attitudes (eight items), subjective norms (three items), perceived behavioral control (five items), and intention (three items), and (2) a TPB-based questionnaire by González et al [28], comprising seven items on behavioral beliefs, three on subjective norms, eight on intention or self-efficacy, and two on perceived control. Both questionnaires, however, share a few shortcomings. Many items are paraphrases of the same question: for example, one of the injunctive norm questions states that “the majority of people important to me want me to exercise […]”; in a second item, “want me” is replaced by “think I should,” and in a third item by “expect me to” [28]. Having 3 almost identical questions about a topic has an illusory benefit of elevated Cronbach α, but otherwise offers no more information than a single question. Moreover, the construct of attitudes in both questionnaires has a very narrow meaning, essentially, “good” as opposed to “bad,” without differentiating between behavioral outcomes and their evaluations [29]. The norm questions in both questionnaires are overly general, referring to “most people I care about.” This is a good solution in a short questionnaire, but it does not enable one to differentiate between sources of social influence. Finally, behavioral control in both questionnaires includes only perceived self-efficacy, whereas in TPB, it “[…] is assumed to reflect past experience as well as anticipated impediments and obstacles factors they [i.e., the respondents] believe could make it easier or more difficult for them to perform the behaviour” [15].

In this paper, we take a bottom-up approach to construct a questionnaire of psychological determinants of PA aimed at university students. Besides being a research instrument, a detailed questionnaire of PA determinants can help develop and evaluate interventions, as well as counsel individuals about health behavior. Our primary aim is content coverage; additionally, we need to consider time limitations: respondents tend to be less attentive and cooperative when asked a large number of similar questions [30].

### This Study

The present study aimed to create a TPB-based questionnaire for university students, in accordance with good practices of creating such questionnaires [29].

Among the available TPB-based questionnaires, none offers a comprehensive coverage of all TPB constructs. In addition to the TPB constructs, we decided to assess 1 additional facet: acceptance of negative consequences. This is potentially important for complex behaviors that may bring about both positive and negative consequences. In TPB, the behavioral attitude is determined by behavioral beliefs and outcome evaluations; if a consequence is believed to follow a behavior with high certainty (behavioral belief) and this consequence is evaluated negatively (outcome evaluation), it should reduce the motivation to engage in that behavior. If the occurrence of a consequence (such as fatigue) cannot be avoided, the only way to protect one’s motivation would thus be to change the evaluation of that consequence. An alternative—accepting a consequence (eg, fatigue) without changing the evaluation—is not explicitly described in TPB. A person who accepts fatigue as a consequence of PA would not avoid PA because of the behavioral belief, but would still avoid other behaviors that bring about similar amounts of fatigue (eg, working too hard or going to sleep too late). One should emphasize that this addition is in principle compatible with the general framework of TPB: one might describe acceptance of negative consequences, for instance, in terms of decision weights to be applied to different perceived consequences—negative consequences that one is willing to accept, matter less to the decision to engage in the behavior.

This study consisted of 3 phases. We started with a qualitative elicitation study to collect ideas and potential items. Thematic analysis [31] of the responses was carried out jointly by the 3 authors of this paper, the results of which were condensed into a draft questionnaire. This was followed by pretesting for clarity and readability. The respondents also provided free-text comments, which were considered in modifying the questionnaire. The modified questionnaire was used in quantitative pretesting. In the following analysis, the aim was to shorten each block of the questionnaire by keeping the most informative items. For this purpose, psychometric analyses, as well as conceptual analysis, were used as tools.

In sum, this study aimed to construct a questionnaire of beliefs and attitudes regarding PA among university students, using thematic analysis of free-response descriptions from the target group as a starting point. The questionnaire will be structured around TPB constructs, complemented by the acceptance of negative outcomes. The questionnaire can be used to assess the PA motivation of young adults when planning interventions (for example, to find out the most salient perceived obstacles to PA), as well as to evaluate the effects of health psychological interventions aiming to increase PA.

## Methods

### Study Design

**Figure 1:**
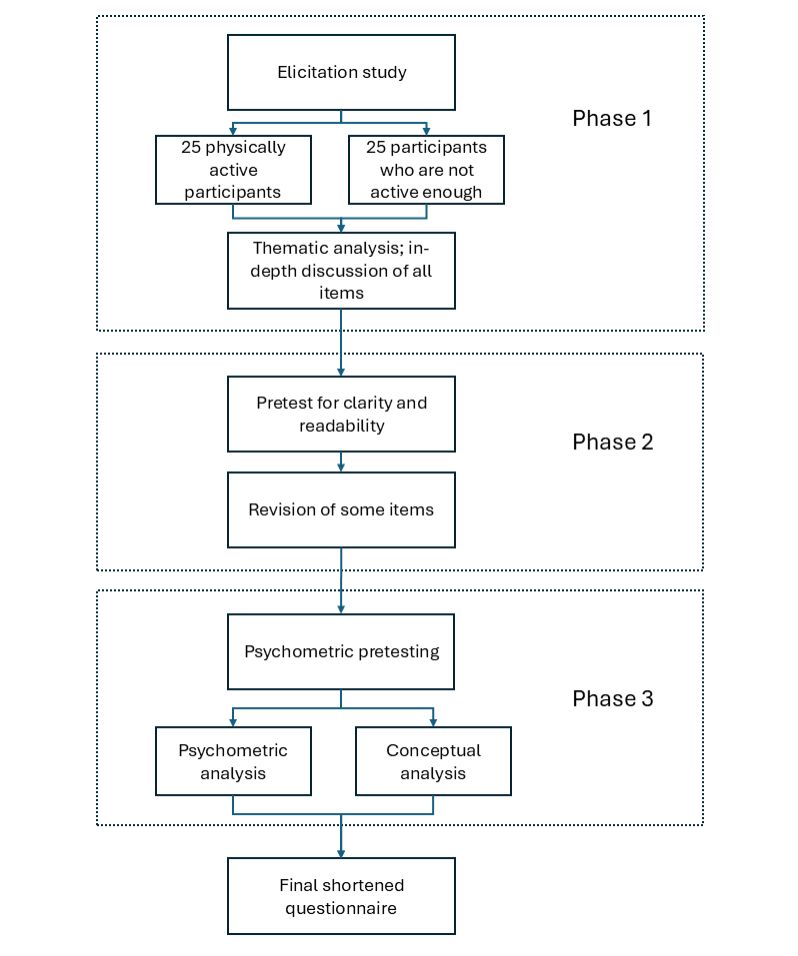
Stages of questionnaire construction in this study.

This study lasted from January 2021 to February 2022. The stages of questionnaire construction in this study are depicted in Figure 1. The data were collected using Google Docs (phase 1) and LimeSurvey (phases 2 and 3), and analyzed using SPSS (IBM Corp; version 28.0.1.1), and R (R Foundation; version 4.3.3) [32].

### Ethical Considerations

This study was conducted in accordance with the Declaration of Helsinki. As per applicable regulations, ethical approval was not required: according to its statute, the Research Ethics Committee of the University of Tartu assesses research projects aiming to collect personally identifiable data or having potential health impact. The committee does not issue opinions on whether or not a study requires ethical approval; in cases of doubt, the researchers can consult their faculty’s research integrity counselor. The present authors’ assessment that ethical approval was not required was confirmed post hoc by the research integrity counselor of the Faculty of Social Sciences.

Participants remained anonymous, and no sensitive or potentially identifying information was collected. Invitations, sent via public university mailing lists, briefly explained this study’s purpose, estimated completion time, and ensured anonymity and research-only use of data. Participants could contact the first author with questions. No compensation was provided.

### Phase 1: Elicitation Study

#### Overview

With the elicitation study, we aimed to generate potential items for the questionnaire, using a web-based survey with free-text answers. The answers were thematically analyzed to create a draft questionnaire covering the most important determinants of PA mentioned by the target group. The elicitation survey, as well as analytic steps in thematic analysis and item generation, were based on guidelines by Francis et al [29].

#### Data Collection

Francis et al [29] recommended a sample size of 25 for the elicitation study. We set the goal of collecting the same number of responses (N=25) from respondents who self-identify as physically active, and from respondents who report not being physically active enough. Data were collected in 2021 by sending an invitation and a link to an anonymous web-based survey to student mailing lists in a public university in Estonia, as well as posting it on social media. Initially (January 15-26), only 25 answers were received, some of which were very brief. Therefore, a second round was carried out (February 19-26), spreading the invitation somewhat more widely, resulting in a higher-than-expected number of answers (n=260). Of the total of 285 respondents, 99 were male, 183 female, 2 nonbinary, and 1 unspecified. The first 25 complete responses from each group (self-identified as active and inactive) were selected for thematic analysis, excluding respondents who provided mostly very short textual responses: for example, “well” in response to the question about one’s feelings after PA, or “none” for the question about the disadvantages of PA. Of the 50 respondents selected for analysis, 35 (70%) were female and 15 were male, with the age ranging from 19 to 41 (mean 24.2, SD 5.0) years.

#### Questionnaire

The questionnaire (Multimedia Appendix 1), based on Francis et al [29], included the following sections: (1) background: gender, age, university, study area, work, and PA habits; (2) perceived benefits of PA for people in general, importance of these benefits to the respondent (rating scale from 1 to 7); (3) disadvantages of being physically active for people in general. importance thereof for the respondent (rating scale from 1 to 7); (4) benefits of being physically active (for self-admitted active respondents) or hypothetical benefits of becoming more active (for self-admitted low active respondents); (5) feelings during and after PA, feelings about the idea of being physically active, what one enjoys or hates about being physically active; (6) people who think the respondent should be more or less physically active; (7) factors that make it easier or more difficult for the respondent to be physically active; (8) other comments on PA; and (9) whether the respondent wants and thinks they need to be more physically active.

#### Thematic Analysis

Thematic analysis [31] was conducted to identify recurrent themes in the answers to each question. After omitting empty responses, the text pool contained 572 separate answers (4642 words), some of which were split into 2 or 3 based on content (eg, when a respondent reported being “tired” and “happy” after PA). As a first step, the first author (TDBN) assigned a code to each answer and grouped synonymous or near-synonymous answers. All coding was reviewed by the second (AH) and third (KK) authors; discrepancies were discussed and resolved by consensus. As a second step, items were written for each section of the questionnaire, corresponding to the constructs of TPB. This was done in group sessions with the participation of all authors; every answer was considered as a potential item. During this process, some items were paraphrased to improve clarity; ambiguous or idiosyncratic answers were omitted. In some cases, item categories were combined into a single item: for example, “(feeling) energized” was not included as a separate item because “more energy” was already included; “benefit to health” was not included because there was already “better health.” All such cases were documented with the reasons for excluding a response from the item pool.

We rephrased some responses to ensure broad applicability. For instance, “less sleep disorders” became “better sleep”—so that respondents with normal sleep would still be able to relate to the item. Similarly, “better mood when you have achieved something” was shortened to “better mood” to cover instances where PA itself was not a major achievement. Responses about PA “taking time” were merged with the category of “less time for other activities” (all activities consume time; the issue is how that time impacts other pursuits). Examples of responses that were omitted from the questionnaire: (1) an evolutionary explanation that all organisms tend to conserve energy and thus avoid effort without a clear need, and (2) an explanation of how PA contributes to the oxygenation of the brain. These were judged to be too specific for most respondents.

### Phase 2: Pretesting for Clarity and Understanding

The second phase involved pilot-testing the 112-item questionnaire (Multimedia Appendix 2) for clarity and understandability, and collecting improvement recommendations. After each of the 9 question blocks, the respondents were prompted: “We would like to receive your feedback on the questions (numbers of relevant questions) that you answered. Were the questions clear? Do you have any recommendations to improve the questions?” In total, there were 49 completed responses, which were all analyzed. The sample consisted of Estonian university students, 85.7% female, with the age range of 19 to 53 (mean 30.5, SD 10.3) years.

### Phase 3: Psychometric Pretesting and Shortening the Questionnaire

#### Overview

The third phase had two goals: (1) reducing items while retaining the maximum amount of information. As the intention was to measure all TPB concepts, item reduction could be used only within a block of questions, not across all blocks, and (2) to show the psychometric properties of the shortened scales. In some cases, this could not be done directly, as some of the items were combined or generalized in the final scale. For example, the “mother” and “father” items in the perceived norms section were combined into a single item, “parents.” In such cases, we used the upward-rounded average of the 2 items. Psychometric methods were used in combination with rational analysis of item content (eg, semantic and logical relationships between items). Additionally, somewhat different criteria had to be used in different blocks.

It should also be noted that the TPB is not a psychometric theory: it makes no predictions about the correlations between items within a given domain (eg, behavioral beliefs or injunctive norms), or between the domains. This means that even though one might be tempted to use an overall confirmatory factor analysis to test the psychometric properties of the questionnaire, such an analysis would be meaningless due to the absence of relevant theoretical predictions. Latent variable models are commonly seen as an essential in questionnaire construction, but their use is too often unjustified and solves fewer problems than expected [33,34]. In TPB, the constructs are not treated as latent variables: the theory does not state that, for instance, the different behavioral beliefs reflect a common cause and should be correlated. For this reason, psychometric analyses (which in most cases imply a form of reflective latent variable model) serve a supporting role in this paper. For example, good internal consistency and clean factor structure are not our primary aims; instead, we aim at maximizing the informativeness and content coverage.

#### Sample and Procedure

Self-report data with the refined questionnaire (Multimedia Appendix 3) were collected from November 2021 to February 2022. Participants were recruited through the several mailing lists of 6 Estonian universities. Of the 797 responses, only complete ones (where the respondent had reached the last page) from people currently studying in a university were used; 349 incomplete responses, as well as one respondent who reported as not to be currently studying, were discarded. The final sample included 447 responses (83% female, 15.4% male, and 1.6% other or unreported gender). The age ranged between 18 and 61 (mean 25.0, SD 7.7) years.

#### Strategy of Item Reduction

In most questionnaire blocks, the following analytic strategy was used for item reduction (an exception is described below):

1. Parallel analysis was used to test dimensionality, with the goal of making sure that each nonrandom dimension of variability in the item set was represented in the final, shortened questionnaire.
2. To interpret the meaning of these dimensions, exploratory factor analysis (EFA) was used in combination with rational analysis of item content.
3. Internal consistency was computed for each block of items. As the previous steps aim at content breadth rather than homogeneity, a high internal consistency is not necessarily expected, and is not a reflection of the quality of the scale. It may happen that there are several distinct and weakly correlated classes within a domain, each of which contributes to the prediction of the outcome. However, it is also possible that the item set is relatively homogenous, which would justify, for example, combining the items into a summary score. This situation is neither hypothesized in TPB nor necessarily desirable, but it is useful to know and can inform future research. We therefore report internal consistency statistics for the final version of each block of items.

This general scheme had to be adapted or modified in a few cases.

1. In the case of behavioral beliefs, an aim was to cover both positive and negative perceived consequences. For negative consequences, conceptual considerations were given more weight than factor analysis, because these items concerned mostly specific physiological conditions (eg, sweating, fatigue, and injuries). Conversely, positive consequences were mostly subjective, with several items being similar in content.
2. Rational analysis occasionally suggested more than one item to represent a factor. For instance, “being satisfied with myself” was included even though there was already an item (“feeling better”) representing the first factor of behavioral beliefs because being satisfied with oneself refers to cognitive evaluation, which is semantically different from just feeling better (affective reaction).
3. For logically related item blocks, the same items were selected for all blocks, considering the empirical results within each block. First, the section of negative perceived consequences had the same items as the section of acceptance of (negative) consequences. Second, the three norm-related item blocks all contained the same items.
4. In phase 2, several respondents perceived the two control-related sections (control beliefs and power of control) as very similar; this was confirmed by high correlations between corresponding items. It was thus decided to conduct a joint factor analysis of these items and to use the reduced set of items only once in the final questionnaire.

Instead of factor analysis, the individual correlations between selected pairs of items were considered in the norm-related blocks. Where possible, we combined the empirically related items into one. This had to be done considering the conceptual relations between categories: for example, merging the items “mother” and “father” into a more general item “parents” makes sense, whereas merging “mother” and “sister” into “female family members” would be less intuitive and “children” have no common denominator with “colleagues,” even if empirically related.

#### Strategy for Showing the Psychometric Properties of the Shortened Scales

We used principal axes factoring, which, differently from ML estimation, does not require multivariate normality [35]. The results of Bartlett test, the Kaiser-Meier-Olkin criterion, as well as skewness and kurtosis of variables were examined before analysis. For domains that can arguably be treated as unidimensional, we examined the data with item response theory (IRT; graded response model [36]) to compare the informativeness of items at different levels of the latent variable. Instead of separate threshold (“difficulty”) parameters, we use the IRF-based location index [37] implemented in R’s *mirt* package [38]. Roughly, this index refers to the value of the latent variable where the expected response of an item is at the scale midpoint. Instead of IRT discrimination parameters, we present factor loadings, which convey essentially the same information but have the advantage of being directly comparable to the EFA factor loadings.

## Results

### Phase 1: Elicitation Study

#### Overview

First, we analyzed each question separately. For instance, the question “What do you think are the benefits of being physically active for people in general?” yielded 117 distinct answers (many respondents provided several answers), which were grouped into 22 categories (each with at least 2 mentions); 21 answers could not be coded. For example, the category “better overall health” included answers such as “stronger health,” “improves overall health,” and “beneficial for health”; the category “better mood” included answers such as “good mood,” “better mood,” and “improved mood.”

#### Behavioral Beliefs and Acceptance of Negative Consequences

The answers referring to beliefs about the consequences of PA were combined into 34 items. Several perceived consequences of PA (such as better mood and fatigue) appeared across multiple questions. Of these 34 items, 26 were positive consequences of PA, and 8 were negative. Initially, we planned to follow the guidelines by Francis et al [29], asking 2 questions for each item: behavioral beliefs (likelihood of PA leading to this outcome) and outcome evaluations (desirability of the outcome). However, we could not effectively incorporate the desirability ratings for 2 reasons. First, all items were either clearly positive or clearly negative, making half of the response scale seem unnatural to many respondents. Think, for instance, of the degree of “undesirability” of good health or “desirability” of injuries. Francis et al [29] noticed the same issue, suggesting omitting “downright silly” questions. Second, the perceived outcomes of PA seem mostly to be matters of degree rather than all-or-none questions. Pain is undesirable, but mild pain is mildly undesirable, and extreme pain is extremely undesirable. Similarly, a good mood is more desirable if it lasts longer. Modifying the items so that the desirability question would make sense would make the section excessively long and complex, requiring additional pretesting. This might involve adding modifiers (eg, “5 extra years of life” or “serious injuries”) or introducing comparisons (eg, desirability of good health compared to good salary). We thus decided to omit the outcome evaluations. As discussed in the Introduction, however, we introduced a section on accepting negative outcomes (8 items).

#### Subjective Norms

For the question about individuals who think the respondent should be more physically active, we identified 32 distinct answers, categorized into 8 groups (mother, father, sisters, friends, colleagues, partner, parents, and family), excluding some unique answers (myself, nobody, and everyone). For a related question about people who think the respondent should not be more physically active, we found 18 distinct answers, categorized into 6 groups (nobody [the most frequent answer]; mother, father, friend, partner, and parents). Ultimately, 8 items were included in the preliminary questionnaire (mother, father, sisters, brothers, friends, colleagues, fellow students, and relatives [excluding parents and siblings]). The same items were used across all 3 norm-related question blocks.

#### Control Beliefs

We received 88 distinct responses for factors making PA easier and 85 for factors making it harder, categorized respectively into 19 and 23 groups, and combined into 24 items. Initially, the items included two instructions to assess control beliefs: (1) strength of belief (eg, “to what extent does the time you spend being physically active, depend on weather”), and (2) power of control belief (eg, “how difficult does bad weather make you to be physically active?”).

We also added two general PA-related self-efficacy and controllability items: (1) “The amount of time that I spend on being physically active is completely up to me,” and (2) “I am confident that I can be more physically active in future.”

### Phase 2: Pretesting for Clarity and Understanding

We reviewed every specific comment to determine if changes were needed in items or instructions; some changes were made in most sections. For instance, we clarified that questions on perceived consequences referred to the respondent personally, not people in general, following feedback. When questions were unclear, we rephrased them to improve readability. In several cases, no changes were made. For example, some of the behavioral belief items were perceived to be redundant by 1 respondent; no items were omitted, though, because that was the task for the third phase.

Most of the changes were related to readability of the items, potential ambiguity, or compatibility among items, or between instructions and items. For example, the item “relatives (except for parents and siblings)” was changed to “relatives (except for parents, siblings and children)” because the intention was to ask about the “other” relatives, not mentioned in previous items (parents, siblings, and children).

### Phase 3: Item Selection and Psychometric Pretesting

#### Overview

Detailed analyses and the final questionnaire are included in online supplements (Multimedia Appendices 4 and 5). Here, we report only the main considerations for each question block.

#### Behavioral Beliefs and Acceptance

We ran parallel analysis on 35 behavioral belief items, suggesting 6 factors that we retained in factor analysis (principal axes method, Varimax rotation; details in Multimedia Appendix 5, Section S2).

We aimed to select no more than half of the items, using the following criteria: (1) each factor should be represented by at least one high-loading item; (2) factors with heterogenous content should be represented by more than one item; and (3) preference is to be given to shorter, more general, less ambiguous and universally applicable items.

Based on these criteria, 6 positive and 8 negative behavioral consequences were selected. For example, better mood, feeling better, and feeling good all had high loadings on the first factor, and are all conceptually similar, so we retained only the highest-loading item. Conversely, “self-satisfaction” loaded on both first (n=0.6) and the second (n=0.4) factors and is conceptually distinct from the 3 items mentioned above (referring to the respondent as the object of the feeling), so we kept it. A detailed description of the considerations in item selection is given in Multimedia Appendix 5, Section S2.

An IRT analysis revealed that while the positive behavioral consequences can be treated as a unidimensional latent variable, the selected 6 indicators all had a relatively low value of the location parameter. Therefore, to use the positive beliefs items as a scale, it will be useful to add a few items with a higher location parameter (eg, “better focus”); Table 1 includes 3 such items in addition to the initial 6 selected for content validity.

**Table 1:** Negative behavioral beliefs and acceptance of negative consequences: factor loadings, IRT^a^ parameters, and correlations with PA^b^.

	Negative behavioral beliefs				Acceptance of the negative consequences of PA		
	λ^c^	*gl* ^d^	*r* ^e^	λ		*gl*	*r*
Feeling pain	0.57	1.35	–0.17^f^	0.48		0.96	0.06
Muscle pain	0.63	–0.51	–0.14^f^	0.65		–1.29	0.25^f^
Injury or injuries	0.39	1.77	0.10^g^	0.41		0.90	0.18^f^
Feeling tired	0.68	0.14	–0.21^f^	0.74		–0.65	0.25^f^
Feeling exhausted	0.84	0.45	–0.24^f^	0.64		0.07	0.15^f^
Sweating	0.37	–3.15	0.09	0.68		–1.68	0.20^f^
Feeling uncomfortable	0.74	0.54	–0.33^f^	0.68		–0.34	0.12^g^
Less time for other activities	0.48	–0.01	–0.14^f^	0.53		–0.29	0.43^f^

^a^IRT: item response theory.

^b^PA: physical activity.

^c^λ: item response theory loading.

^d^*gl*: generalized location parameter.

^e^*r*: correlations with self-reported physical activity.

^f^*P*<.01.

^g^*P*<.05.

Negative consequences showed mostly positive and significant correlations, averaging 0.30 (SD 0.13), and ranging from 0.08 (sweating vs less time for other activities) to 0.59 (feeling exhausted vs feeling uncomfortable). Positive consequences were all positively correlated with an average of 0.49 (SD 0.13) and ranged from 0.04 (stable weight vs weight gain) to 0.84 (feeling better vs peace of mind). Notably, items referring to subjective experiences were highly intercorrelated: for example, the correlation between “more energy” and “better mood” (arguably distinct concepts) was 0.69. Cross-correlations between negative and positive consequences were mostly negative, averaging –0.12 (SD 0.12) and ranging from –0.39 (more energy vs feeling pain) to 0.24 (muscle pain vs weight gain due to an increase in muscle mass). See Multimedia Appendix 6 for a full table of correlations.

Finally, acceptance of negative consequences items were all positively and significantly correlated, averaging 0.31 (SD 0.10) and ranging from 0.12 (sweating vs feeling pain) to 0.49 (sweating vs feeling uncomfortable and feeling tired vs feeling exhausted).

For positive behavioral beliefs, the first principal axes factor explained 51.9% of variance, to which the next unrotated factor added 4.1%. The shortened scale closely approximates the full version, with a strong correlation of 0.94 (*P*<.001). Table 2 presents the IRT factor loadings and location parameters.

**Table 2:** Positive behavioral beliefs: IRT^a^ factor loading and generalized location parameter.

	λ^b^	*gl* ^c^
Feeling better	0.87	–1.49
Better physical health	0.87	–1.77
Better long-term health^d^	0.82	–0.91
Stable weight	0.49	–1.43
Staying in shape	0.83	–1.39
Self-satisfaction	0.80	–1.50
More energy	0.76	–0.85
Better focus	0.78	–0.77
Greater work capacity	0.84	–0.73

^a^IRT: item response theory.

^b^λ: item response theory factor loading.

^c^*gl*: generalized location parameter.

^d^This item was a generalization of 2 items: preventing health problems and a longer lifespan. The upward-rounded average of these items was used in the analysis.

We omitted “weight gain” from negative behavioral beliefs and acceptance of negative consequences due to its ambiguity, low correlation with other negative items, and limited relevance for typical PA intervention targets. The remaining 8 items had an average correlation of 0.3, with a Cronbach α of 0.78 and McDonald ω of 0.83. The first factor explained 32%, to which the next unrotated factor added 6%.

The average interitem correlation of acceptance items was 0.31, with a Cronbach α of 0.79 and McDonald ω of 0.85. The first factor explained 32.1% of the variance, to which the second factor added 13%.

Correlations between negative behavioral beliefs and acceptance of the corresponding outcome were generally low, ranging from –0.15 to 0.26. Sum scores of negative behavioral beliefs and acceptance had opposite correlations with self-reported PA: –0.22 and 0.33 (both *P*s<.001). Table 1 presents the IRT parameters of negative behavioral beliefs and acceptance items and their correlations with self-reported PA.

#### Subjective Norms

Correlations between items of injunctive norms and motivation to comply were high, positive, and statistically significant (average *r*=0.62, SD 0.11 and *r*=0.57, SD 0.11, respectively). Correlations between conceptually related item pairs (such as mother vs father, brothers vs sisters, and colleagues vs other students) were somewhat higher than average. However, descriptive norms had generally lower positive correlations (average *r*=0.24, SD 0.15), and not all were statistically significant (Multimedia Appendix 5, Section S3).

Average correlations calculated across 3 matrices are shown in Table 3.

**Table 3:** Average correlations between subjective norms (mean of 3 correlation matrices: injunctive norms, descriptive norms, and motivation to comply).^a^

			1. My mother		2. My father	3. My brothers		4. My sisters		5. My friends		6. My colleagues		7. Other (university) students		8. My partner		9. My children
1. My mother																		
	*r*	1		0.59^IDM^		0.49^IDM^	0.54^IDM^		0.37^IM^		0.33^IdM^		0.30^IM^		0.41^IdM^		0.36^IM^	
	*P* value	—^b^		—		—	—		—		—		—		—		—	
2. My father																		
	*r*	0.59^IDM^		1		0.55^IDM^	0.46^IdM^		0.37^IM^		0.36^IdM^		0.35^IdM^		0.39^IM^		0.34^IM^	
	*P* value	—		—		—	—		—		—		—		—		—	
3. My brothers																		
	*r*	0.49^IDM^		0.55^IDM^		1	0.67^IDM^		0.48^IDM^		0.45^IM^		0.41^IM^		0.42^IdM^		0.55^IDM^	
	*P* value	—		—		—	—		—		—		—		—		—	
4. My sisters																		
	*r*	0.54^IDM^		0.46^IdM^		0.67^IDM^	1		0.49^IDM^		0.47^IdM^		0.41^IdM^		0.50^IDM^		0.65^IDM^	
	*P* value	—		—		—	—		—		—		—		—		—	
5. My friends																		
	*r*	0.37^IM^		0.37^IM^		0.48^IDM^	0.49^IDM^		1		0.60^IDM^		0.55^IDM^		0.54^IDM^		0.64^IDM^	
	*P* value	—		—		—	—		—		—		—		—		—	
6. My colleagues																		
	*r*	0.33^IdM^		0.36^IdM^		0.45^IM^	0.47^IdM^		0.60^IDM^		1		0.66^IDM^		0.34^IM^		0.64^IDM^	
	*P* value	—		—		—	—		—		—		—		—		—	
7. Other (university) students																		
	*r*	0.30^IM^		0.35^IdM^		0.41^IM^	0.41^IdM^		0.55^IDM^		0.66^IDM^		1		0.35^IM^		0.46^IdM^	
	*P* value	—		—		—	—		—		—		—		—		—	
8. My partner																		
	*r*	0.41^IdM^		0.39^IM^		0.42^IdM^	0.50^IDM^		0.54^IDM^		0.34^IM^		0.35^IM^		1		0.55^IM^	
	*P* value	—		—		—	—		—		—		—		—		—	
9. My children																		
	*r*	0.36^IM^		0.34^IM^		0.55^IDM^	0.65^IDM^		0.64^IDM^		0.64^IDM^		0.46^IdM^		0.55^IM^		1	
	*P* value	—		—		—	—		—		—		—		—		—	
10. Relatives (other than parents, siblings, and children)																		
	*r*	0.50^IDM^		0.48^IDM^		0.53^IDM^	0.49^IDM^		0.50^IDM^		0.57^IDM^		0.55^IDM^		0.38^IdM^		0.53^IDM^	
	*P* value	—		—		—	—		—		—		—		—		—	

^a^Subscript letters denote significance levels (*P* values) for each correlation matrix. I: injunctive norms; D/d: descriptive norms; M: motivation to comply. Lowercase letters: *P*<.05; capital letters: *P*<.01. For example, the superscript IDM means that the correlation was significant at 0.01 level in all 3 matrices; the superscript IM means that the correlation was significant at 0.01 level in injunctive norms and motivation to comply, but not in descriptive norms; the superscript IdM means that the correlation was significant at 0.01 level for injunctive norms and motivation to comply, but at 0.05 level for descriptive norms.

^b^Not available.

For the final questionnaire, we combined items based on their correlations, as well as common sense. For example, the items “my mother” and “my father” had a moderate correlation (n=0.59), making it reasonable to combine them as “parents.” However, despite a moderate correlation between “my friends” and “my children” (n=0.64), combining them would not be appropriate, as they do not form a natural category. We omitted the item “other relatives” due to its heterogeneity and varying personal meanings, as well as its moderate-to-high correlations with other categories. In sum, the number of items in all 3 sections was reduced from 10 to 6: parents, siblings, friends, colleagues/costudents, partner, and children.

For injunctive norms, the first factor explained 64.4% of the variance, with the second factor adding 7.1%. The average correlation among the 8 items was 0.61, with Cronbach α of 0.91 and McDonald ω of 0.96. For descriptive norms, the first factor explains 32% of the variance, with the second factor adding 11.8%. The average correlation among the items was 0.29, with a Cronbach α of 0.67 and McDonald ω of 0.84. The Kaiser-Meier-Olkin value was 0.56, indicating that the data may not be suitable for factor analysis. For motivation to comply, the first factor explained 58.4% of the variance, whereas the second one added 30.1%. The average correlation among the items was 0.67, with a Cronbach α of 0.89 and a McDonald ω of 0.96. Table 4 presents the IRT factor loadings and location parameters for these constructs.

**Table 4:** Perceived norms and motivation to comply: factor loadings and IRT^a^ parameters.

	Injunctive norms			Descriptive norms			Motivation to comply	
	λ^b^	*gl* ^c^	λ		*gl*	λ		*gl*
Parents	0.74	0.51	0.27		–0.16	0.86		–0.10
Siblings	0.86	0.68	0.44		–0.67	0.88		0.15
Friends	0.91	0.68	0.67		–0.50	0.83		–0.17
Colleagues and fellow students	0.94	0.89	0.55		–0.32	0.72		0.85
Partner	0.80	0.30	0.42		–0.60	0.83		–0.98
Children	0.86	0.56	0.83		–0.95	0.82		–0.64

^a^IRT: item response theory.

^b^λ: item response theory loading.

^c^*gl*: generalized location parameter.

#### Control Beliefs and Self-Efficacy

The 2 self-efficacy and controllability items were moderately correlated (*r*=0.38, *P*<.01); both were included in the final questionnaire.

Parallel analysis of 48 control belief and perceived power items suggested 12 factors. Based on this, a factor analysis was carried out using 12 factors (principal axes with Varimax rotation; details Multimedia Appendix 5, Section S4). Most factors were clearly identifiable and interpretable; it was therefore decided to keep at least 1 item for each factor. A few items were rewritten in a more general way: for example, instead of the “gym,” the item in the final version refers to the “place to be physically active” (subsuming, eg, gyms, parks, and tennis courts). We sometimes retained more than 1 item per factor: for example, the items “weather” and “the place where I live” loaded on the same factor but refer to clearly distinct determinants (one is changeable and not under the person’s control, whereas the second is rather stable, and depends on the respondent’s own choices). Two items referring to safety (“living in an unsafe environment” and “whether the environment is safe”) were at best weakly related to any of the factors; however, it is known that safety concerns can be an important obstacle to PA [39]. It was therefore decided to keep a paraphrase of the initial items (“the safety of the place where I live”) in the final questionnaire. The paraphrase was intended to make the item less ambiguous, referring to the safety of one’s neighborhood, and making other interpretations of the original items (eg, air pollution or geopolitical concerns) less likely.

In the shortened version, parallel analysis suggested 4 factors. Unlike in other domains, the factors beyond the first one were of substantial size (the first 4 Promax-rotated EFA factors explained, respectively, 14%, 7%, 11%, and 8% of the variance). It is therefore not meaningful to treat these responses as indicators of a single latent variable, even though the correlations were mostly positive (average 0.23, ranging from –0.16 to 0.82). The factors had 1 to 6 indicators (loadings above 0.4). Table 5 presents the factor loadings from a 4D graded response model analysis.

**Table 5:** Control beliefs: IRT^a^ loadings and factor correlations (Promax rotation)^b^.

Item				F1^c^		F2	F3		F4
	The weather		0.40		0.18		–0.28	0.16	
	How I am feeling		–0.01		0.68		0.07	0.15	
	My health		–0.14		0.74		0.07	–0.10	
	My planning skills		–0.03		0.46		0.13	–0.04	
	How fast I see the results		0.63		0.02		–0.17	0.04	
	Whether I have time or not		–0.04		0.07		0.08	0.97	
	Whether there is a gym nearby		0.10		0.04		0.90	0.07	
	Whether the gym is open or not		0.06		0.04		0.98	0.01	
	Whether I have a car or not [...]		0.62		–0.24		0.19	–0.02	
	Having the necessary equipment		0.62		0.00		0.19	–0.05	
	My financial situation		0.45		0.10		0.26	–0.06	
	Where I live		0.66		–0.03		–0.02	–0.01	
	Whether the environment is safe		0.29		0.38		–0.22	–0.07	
	Whether I have someone to do it with or not [...]		0.41		0.05		0.03	–0.03	
Factor correlations									
	F1								
		*r*	1.00		0.53		0.34	0.30	
		*P* value	—^d^		—		—	—	
	F2								
		*r*	0.53		1.00		–0.09	0.43	
		*P* value	—		—		—	—	
	F3								
		*r*	0.34		–0.09		1.00	–0.06	
		*P* value	—		—		—	—	
	F4								
		*r*	0.30		0.43		–0.06	1.00	
		*P* value	—		—		—	—	

^a^IRT: item response theory.

^b^Some items were shortened for presentation.

^c^F: factor.

^d^Not available.

#### Behavioral Intentions

Two items referring to behavioral intentions correlated *r=*0.50 (*P*<.01) and were both included in the final questionnaire.

#### Predicting Self-Reported PA

Table 6 shows the correlations between the item bundles and self-reported PA.

**Table 6:** Correlations of item bundles with self-reported physical activity^a^.

	Full		Shortened	
	*r*	*P* value	*r*	*P* value
Positive beliefs	0.29	<.001	0.26	<.001
Positive beliefs (2)	N/A^b^	N/A	0.29	<.001
Negative beliefs	–0.18	<.001	–0.22	<.001
Acceptance	0.32	<.001	0.32	<.001
Injunctive norms	–0.40	<.001	–0.39	<.001
Descriptive norms	0.11	.02	0.15	.002
Motivation to comply	0	.98	0	.94
Control beliefs (4 EFA^c^ factors and multivariate prediction)	0.48	.004	0.48	<.001

^a^For positive behavioral beliefs, 2 shortened versions were considered: 6 items selected to optimize content validity (first line) and 9 items, including the aforementioned 6, and adding 3 with the highest location parameter from IRT. For perceived norms, person means across all completed items were used (eg, if the respondent does not have children, then the mean of all other items was used). For control beliefs, 4 factor scores were used to predict the criterion, and the *r* refers thus to the multivariate model (square root of *R*^2^).

^b^N/A: not applicable.

^c^EFA: exploratory factor analysis.

## Discussion

### Principal Findings

This study aimed to create a questionnaire based on the TPB for university students and evaluate its psychometric properties. Additionally, the questionnaire is meant for evaluating the effectiveness of health psychological PA interventions in university students and young adults.

This study consisted of three different phases: (1) an elicitation study for item generation; (2) pretesting for clarity and readability; and (3) item reduction and evaluation of psychometric properties. This resulted in a questionnaire consisting of the following sections: behavioral beliefs regarding PA (14 items), acceptance of negative consequences of PA (6 items), injunctive norms, descriptive norms, and motivation to comply (6 items each), self-efficacy and subjective controllability (2 items), control beliefs (14 items), and behavioral intention (2 items).

Our focus was on covering as many of the different aspects of each construct as possible. This led us, for example, to reduce the number of positive behavioral beliefs more than the number of negative behavioral beliefs. Of the 13 items loading over 0.5 on the first rotated factor, most were related to subjective experiences, and although many of them (eg, better mood and reduced stress) are distinct concepts, the correlations between any pairs of items were considerably higher than the average correlation between the negative perceived consequences.

Our shortened questionnaire is thus usable in situations where the goal is to get a comprehensive overview of the perceived determinants of PA behavior. If the goal is to assess some of the determinants (eg, perceived positive consequences of PA) in more detail or with a greater level of accuracy, one might select more items from these domains.

Before proceeding to a more general discussion, we briefly summarize the most important considerations regarding the 3 phases of this study.

### Phase 1: Elicitation Study

We used thematic analysis to identify recurrent themes mentioned in free-text responses. In thematic analysis, it is important that the authors acknowledge the role of their own theoretical position [31]; we used TPB as a starting point, but used common sense and consistency where appropriate. For example, “brothers” were added to injunctive norms despite not being mentioned in the elicitation study; the same items were used for injunctive and descriptive norms, as well as negative perceived consequences and acceptance. In making judgments about which responses to combine, or whether or not to include an item in the questionnaire, every such item was discussed between the 3 authors until consensus was reached. For example, the answer “counterbalance to the sedentary lifestyle” was deemed ambiguous because (1) it was interpreted as a reason for engaging in PA rather than a benefit of it, and (2) the specific positive effect of this counterbalancing was unclear (eg, whether the respondent meant a positive health effect, or reduction in the negative effect of prolonged sitting, or a feeling of self-satisfaction derived from doing something good for one’s health, etc). Finally, some subjectivity was inevitable in the judgment of thematic saturation. We considered that the analysis of 50 responses yielded more distinct items in each category than could be reasonably included in a final questionnaire (eg, 34 behavioral belief items, 20 of which were based on more than one free-text answer, and the remaining 14 were in most cases closely related variants or nuances of the more frequently mentioned consequences of PA). However, the frequent responses (mentioned at least 3 times) were almost all included in the draft questionnaire, whereas infrequent ones (mentioned only once) were more often judged to be too unusual or ambiguous (with a few exceptions: eg, pain and avoiding weight gain as consequences of PA, and sisters and colleagues as sources of social influence were mentioned only once or twice in the elicitation study). It was thus decided not to apply thematic analysis to the rest of the responses, as it was unlikely to result in new important themes that could realistically be included in the questionnaire.

### Phase 2: Pretesting for Clarity and Readability

This phase aimed to check the clarity and readability of the whole questionnaire, including the instructions, and relationships between item blocks. Participants’ comments often highlighted issues with interitem relationships (eg, redundancy within item blocks, or comparing 1 item block with another) or instructions (eg, linguistic compatibility between an item and the instructions). The resulting changes were focused on clarifying the content of the items and instructions, thus reducing the error variance due to different understandings of the items.

### Phase 3: Shortening the Questionnaire While Preserving Content Validity

In this phase, we used psychometric methods to reduce the number of items in the questionnaire, prioritizing content validity over other psychometric considerations. While none of the psychometric properties were an aim in themselves, it should be noted that the resulting scales do have a moderate to high degree of internal consistency, which may justify the use of sum-scores when this is needed (except for control beliefs).

We presented evidence regarding construct validity and reliability of the shortened scales. Item bundles of positive and negative behavioral beliefs, acceptance of unfavorable outcomes, injunctive and descriptive norms, as well as motivation to comply, can be meaningfully used as sum scores, even if not always strictly unidimensional. Notably, these shortened item bundles match or even exceed the unshortened versions in predicting self-reported PA.

### Comparison With Previous Studies

For behavioral beliefs, the results in our elicitation study largely mirrored earlier research [13], showing commonly cited advantages such as improvements in physical and psychological health, weight control, increased energy, stress relief, and relaxation. There were, however, a few differences. First, the theme of “improved daily functioning” emerged in more than 2/3 studies reviewed by Downs and Hausenblas [13] but was represented by a single item (“greater working capacity”) in our study. Similarly, improvements in social life (eg, making friends, meeting new people, and socializing) were often mentioned in other studies, but were not found in our study. This is likely a characteristic of the student sample: the participants have generally no major issues with daily functioning, and live in a varied social environment with plenty of opportunities to make friends and meet new people, making these less salient as advantages of PA. If, however, one wants to use the questionnaire in a more general sample, one should consider either a new elicitation study, at least for behavioral beliefs. Several perceived consequences of PA mentioned in the review by Downs and Hausenblas [13] were highly correlated in our empirical pretest (phase 3): for example, improved mood, feeling good, having more energy, and decreased stress, had all high loadings on the same factor, and were represented by a single item in our shortened questionnaire (feeling better). Some researchers might be interested in assessing more specific beliefs about subjective consequences (related to mood, focus, energy, etc) of PA and might thus need to include more items from factor 1 of behavioral beliefs (Multimedia Appendix 5 and Table 4).

The most frequent disadvantages of PA in earlier research [13] were health issues, mostly related to pain and injury. This and other frequent categories (fatigue and lack of time) were also represented in our study. The notion of PA being dangerous (eg, fear of another heart attack) did not emerge in our sample, probably because of the low prevalence of any major health issues. Additionally, the everyday inconveniences (eg, interference with family routine or changes in meal times) did not emerge in our study as a separate theme but would likely be more prevalent in other age groups.

For normative beliefs, most elicitation studies reviewed by Downs and Hausenblas [13] reported family members and friends, as well as health care professionals. The latter category (and several others that emerged in some elicitation studies: boss, teacher, coach, other exercisers, church members, media, and other people with cancer) did not emerge in our study, and, again, might be important for other target groups.

Downs and Hausenblas [13] identified health issues, inconvenience, lack of motivation or energy, lack of time, and lack of social support as common categories of control beliefs. Feelings of motivation and energy were loaded, in our phase 3, on the same factor as mood, and were thus not included in the shortened questionnaire.

### Limitations and Directions for Future Research

The strengths of our approach include a reasonably large sample, as well as having a specific target group, allowing us to compile an extensive list of PA determinants. The list is, however, not necessarily comprehensive for other target groups. Due to the age, lifestyle, and typically good health condition of students, some themes did not emerge in the elicitation study; for example, medical professionals were not mentioned as relevant for injunctive or descriptive norms. As another example, items related to daily functioning, and potential dangers related to vigorous PA (eg, experiencing a heart attack) were not mentioned in the elicitation phase. Finally, inconveniences related to everyday routine and family routine were not mentioned and thus not included in the questionnaire. These themes would be likely to be elicited in different (eg, older) target groups.

Some researchers may be interested in making more fine-grained distinctions among the positive consequences of PA [40]. In these cases, we would recommend complementing the shortened questionnaire with extra items based on either our initial questionnaire or the extant literature.

In retrospect, our initial instructions for the sections on control beliefs and strength of control were too similar. Control beliefs refer to a factor’s influence on PA (eg, weather’s impact on exercise), while strength of control refers to the difficulty posed by an unfavorable factor (eg, bad weather making PA difficult). In theory, these are different questions: for example, one may perceive the difficulty of exercising in bad weather but still do it, resulting in weather having little influence on PA. Questions about control beliefs are typically framed as a likelihood of a favorable or unfavorable factor. This would have been cumbersome in our case: for instance, without going into specifics, it is not possible to say how likely “bad weather” is (there are various degrees and kinds of “bad weather”). After the quantitative pretesting, it became evident that asking these different questions would not make sense, but also that it is relatively easy to ask about the perceived power of the factors influencing one’s PA, but more difficult to ask about the perceived likelihood of these factors. This is an area that needs further investigation, as there is substantial evidence about the role of perceived behavioral control in, for example, moderating the intention-behavior relationship [41], but in most studies, the relative contribution of control belief strength and power of control factors is not investigated (a product of these 2 terms is used; O’Sullivan et al [42] have described the methodological problems that this practice creates).

Future studies could explore alternative framings of the behavioral control questions: for example, self-efficacy of overcoming a specific barrier (such as exercising in bad weather), self-efficacy of creating a favorable situation, temptation to use a given obstacle as an excuse (eg, not exercising and referring to bad weather), and perhaps also self-efficacy of overcoming this temptation. It is an empirical question whether these alternative framings improve the prediction of intention or behavior, and whether the respondents would perceive these as distinct questions.

Another potentially important avenue of research is expanding the concept of intention in TPB. For example, in self-determination theory [43], there is a crucial distinction between intrinsic and extrinsic motivation, as well as a taxonomy of psychological needs that may underlie different intentions.

Even though several potentially important themes are missing, our shortened questionnaire is considerably broader in content coverage than most of the readily available alternatives. This, on the one hand, gives the researcher more confidence that important determinants are included; however, long questionnaires may be a source of frustration and boredom to participants [30] and may not be necessary for each purpose. Therefore, an even shorter inventory might be useful—but we would suggest constructing it empirically, looking for the most important predictors of behavior, or the strongest moderators of the intention-behavior relationship. An even shorter questionnaire is, thus, beyond the scope of this study.

In this study, we aimed at creating a comprehensive inventory, focusing thus on content validity [44], or, more specifically, on readability, relevance, and content coverage of the items. Among the other aspects of validity to be investigated in future studies, we would prefer to highlight 2. First, if a measure is to be useful in the context of intervention studies, it needs to be sensitive to change in attitudes. One way to investigate this would be a combination of qualitative and quantitative methods to determine whether the questionnaire captures the change in attitudes that people perceive as important. Second, predictive and construct validity need to be investigated: for example, the degree to which the measure predicts behavior or behavioral intention.

The present questionnaire was developed in Estonian; the English translation is provided only for reference and needs additional pretesting before use. Additionally, the cultural variability of PA-related attitudes and beliefs was not addressed in the present research but needs attention in future studies. Besides culture, other potential sources of influence include age, gender, health and weight status, and socioeconomic background. This study was conducted with university students; even though education in public universities in Estonia is mostly free, socioeconomic disparities exist [45], making it likely that persons with the lowest socioeconomic status were excluded from the sample. An adaptation of the present questionnaire to another cultural, demographic, or socioeconomic context should ideally include a new elicitation study, possibly using the present questionnaire as a starting point, focussing on new topics that are not yet included.

Finally, some of the TPB constructs were not assessed in this study: evaluation of negative outcomes was replaced by acceptability, and evaluation of positive outcomes was omitted. We made these decisions because the outcomes in question (eg, good mood or pain) come in degrees (a very good mood is more desirable than just a little bit good mood, etc) and a general desirability or undesirability question does not make sense. Asking about the desirability of specific degrees of such outcomes would make the questionnaire longer; moreover, the degrees of subjective states are, by definition, also subjective. We could, however, ask about the acceptability of negative outcomes, which is related but not identical to the concept of outcome evaluations, and also related to the TPB construct of controllability: if a given outcome is acceptable, it has therefore less control over the respondent’s behavior.

This decision makes it impossible to compute the combined TPB scores for attitudes. We should, however, note that the use of product scores (eg, likelihood of an outcome multiplied by its desirability) is questionable on methodological grounds, creating a so-called “expectancy-value muddle” [42] and blurring the contribution of the individual components: the multiplication makes good sense conceptually but may fail for measurement reasons. First, it is a good practice to include interaction (product) terms in a regression analysis only after including the corresponding 2-order terms [46]. Second, subjective probabilities are essentially different from actual probabilities [47], and the question of whether they can be meaningfully multiplied needs additional research.

### Conclusions

In this paper, we used a bottom-up approach to construct a questionnaire of determinants of PA in university students. To develop the initial pool of items retrieved from the elicitation study into a questionnaire, we used a combined approach based on free-response comments from the target group, factor analysis, and expert discussions considering match with theoretical constructs, as well as logical and common-sense considerations, with the principal aim of reducing redundancy while retaining informativeness. The shortened questionnaire includes a reasonably comprehensive set of determinants as described by TPB (with a few justified omissions and additions) for this target group. For other target groups, additional factors might need to be considered (eg, medical experts as a source of injunctive norms or heart attack as a possible adverse result of PA). Further research is needed to investigate the predictive and construct validity of the questionnaire, as well as its sensitivity to change in beliefs and attitudes. Finally, some types of studies may need a shorter questionnaire, or more nuanced measurement of some specific determinants (eg, more differentiation in mood states believed to result from PA).

## Appendix

Multimedia Appendix 1 Phase 1 (elicitiation study) questionnaire.

Multimedia Appendix 2 Initial version of the questionnaire.

Multimedia Appendix 3 Questionnaire modified according to the results of phase 2 (pretesting for clarity and understandability).

Multimedia Appendix 4 Final shortened questionnaire.

Multimedia Appendix 5 Descriptive statistics and factor analysis results.

Multimedia Appendix 6 Correlations among positive and negative perceived consequences of physical activity.

Multimedia Appendix 7 Shortened questionnaire: assumption checks and psychometric analyses.

## Abbreviations

ACT: acceptance and commitment therapy
EFA: exploratory factor analysis
IRT: item response theory
PA: physical activity
TPB: theory of planned behavior

## Data Availability

Anonymous data from the third phase are available online [48]. The data from the first and second phases are available from the authors upon reasonable request.
