# Supplementary material for: Toward Comprehensive Assessment of Beliefs and Attitudes Related to Physical Activity in Young Adults: Pilot Study
Source: JMIR Form Res. 2025 Oct 16;9:e69094. doi: 10.2196/69094 (PMC12576300; doi:10.2196/69094)
Supplement: Multimedia Appendix 1 [file formative_v9i1e69094_app1.docx]

**Questionnaire for first phase in English**

Hi, thank you for taking the time to fill in this questionnaire. In the beginning of the questionnaire we will ask you some questions about your background, after that we will ask you questions related to being physically active. The questionnaire should not take more than 15 minutes to fill in. Only fill in the questionnaire if you are a full-time university student and you think you are not physically active enough.

1. Gender

- Male
- Female
- Other

1. Age
2. Name of your university?
3. What do you study?
4. Do you work? If so, what job do you have and how many hours during the week do you work?
5. Describe your ordinary physical activity habits: how often, for how long, and what kinds of activities do you do regularly? Use no more than 1-3 sentences. Physical activity is defined as "any type of movement that requires a certain amount of effort that involves a significant increase in heart rate / breathing frequency (e.g., walking, working out, cycling, jogging and tennis).
6. What do you think are the benefits of being physically active for people in general?
7. How important are these benefits to you?

Not important at all (1) - - - - - - - - - - - - - - - - - - - - - - - - - - - - - - - - - - - Very important (7)

1. What do you think are the disadvantages of being physically active for people in general?
2. How important are these disadvantages to you?

Not important at all (1) - - - - - - - - - - - - - - - - - - - - - - - - - - - - - - - - - - - Very important (7)

1. If you think you are physically active enough, what benefits do you get from being physically active?
2. If you think you are not physically active enough, what benefits could you get from being more physically active?
3. How do you feel while you are physically active?
4. How do you feel after being physically active?
5. How do you feel about the idea of being physically active?
6. What do you enjoy / hate about being physically active?
7. Are there any people (who matter to you) who think you should be (more) physically active? Who are they?
8. Are there any people (who matter to you) who think you should NOT be physically active (OR : be LESS physically active)? Who are they?
9. What factors or circumstances make it easier for you to be physically active?
10. What factors or circumstances make it difficult or impossible for you to be physically active?
11. Is there anything else that comes to your mind when you think about being physically active?
12. How much do you feel like you want to be more physically active?

Very little (1) - - - - - - - - - - - - - - - - - - - - - - - - - - - - - - - - - - - - - - - - - - - - - - - - - - - A lot (7)

23. How much do you feel like you need to be more physically active?

Very little (1) - - - - - - - - - - - - - - - - - - - - - - - - - - - - - - - - - - - - - - - - - - - - - - - - - - - A lot (7)

**Questionnaire for first phase in Estonian**

Tere, aitäh, et leidsid aega selle küsimustiku täitmiseks. Küsimustiku alguses esitame mõned küsimused sinu tausta kohta, pärast seda kehalise aktiivsusega seotud küsimusi. Küsimustiku täitmine ei peaks võtma rohkem kui 10 minutit. Vasta küsimustikule ainult siis, kui sa oled täiskoormusega üliõpilane ja arvad, et sa pole piisavalt füüsiliselt aktiivne.

1. Sugu

- Mees
- Naine
- Other

1. Vanus
2. Ülikooli nimi?
3. Mida sa õpid?
4. Kas sa töötad? Kui jah, siis mis tööd sa teed ja mitu tundi nädalas?
5. Kirjelda oma tavalisi kehalise tegevuse harjumusi: kui tihti, kui kaua ja milliseid tegevusi sa regulaarselt teed? Kasutage mitte rohkem kui 1-3 lauset. Kehaline aktiivsus on määratletud kui "igasugune liikumine, mis nõuab teatud pingutust, mis hõlmab südame löögisageduse / hingamissageduse olulist suurenemist (nt kõndimine, treenimine, jalgrattasõit, jooksmine ja tennis).
6. Mis on sinu arvates kehaliselt aktiivne olemise eelised inimestele üldiselt?
7. Kui olulised on need eelised sinu jaoks?

Pole üldse oluline (1) - - - - - - - - - - - - - - - - - - - - - - - - - - - - - - - - - - - - - - - Väga oluline (7)

1. Mis on sinu arvates kehaliselt aktiivne olemise puudused inimeste jaoks üldiselt?
2. Kui olulised on need puudused sinu jaoks?

Pole üldse oluline (1) - - - - - - - - - - - - - - - - - - - - - - - - - - - - - - - - - - - - - - - Väga oluline (7)

1. Kui sa arvad, et oled kehaliselt piisavalt aktiivne, siis millist kasu saad kehaliselt aktiivsest tegevusest?
2. Kui sa arvad, et sa pole piisavalt kehaliselt aktiivne, siis millist kasu võiksid saada, kui oleksid kehaliselt aktiivsem?
3. Kuidas sa ennast tunned, kui oled kehaliselt aktiivne?
4. Kuidas sa tunned end pärast kehalist aktiivsust?
5. Kuidas sa suhted ideesse olla kehalist aktiivne?
6. Mis sulle kehalise aktiivse olemise juures meeldib / ei meeldi?
7. Kas on inimesi (kes on sulle tähtsad), kes arvavad, et sa peaksid olema (rohkem) kehaliselt aktiivne? Kes nad on?
8. Kas on inimesi (kes on sinu jaoks olulised), kes arvavad, et sa EI peaks olema kehaliselt aktiivne (VÕI: olema vähem kehaliselt aktiivne)? Kes nad on?
9. Millised tegurid või asjaolud hõlbustavad sinu kehaliselt aktiivsust?
10. Millised tegurid või asjaolud muudavad sinu jaoks kehaliselt aktiivne olemise raskeks või võimatuks?
11. Kas sul oleks veel midagi lisada, mis tuleb pähe, kui mõtled kehalisele aktiivsusele?
12. Mil määral sa tunned, et tahaksid olla kehaliselt aktiivsem?

Väga vähe (1) - - - - - - - - - - - - - - - - - - - - - - - - - - - - - - - - - - - - - - - - - - - - - - Väga palju (7)

1. Mil määral sa tunned, et pead olema kehaliselt aktiivsem?

Väga vähe (1) - - - - - - - - - - - - - - - - - - - - - - - - - - - - - - - - - - - - - - - - - - - - - - Väga palju (7)
