# Supplementary material for: Toward Comprehensive Assessment of Beliefs and Attitudes Related to Physical Activity in Young Adults: Pilot Study
Source: JMIR Form Res. 2025 Oct 16;9:e69094. doi: 10.2196/69094 (PMC12576300; doi:10.2196/69094)
Supplement: Multimedia Appendix 2 [file formative_v9i1e69094_app2.docx]

**Questionnaire for second phase English**

**Theory of planned behavior questionnaire for university students**
Hi, thank you for taking the time to fill in this questionnaire. In the beginning there will be some questions about your background and after that we will ask you questions related to being physically active. Throughout the questionnaire we ask you to evaluate the questions that you answer (e.g., "were the questions clear" and "do you have any recommendations to improve the questions?"). We ask you to be as critical as possible when evaluating the questions. This will help us to improve the questionnaire. Filling in the questionnaire should not take more than 20 minutes. Only fill in the questionnaire if you are a full-time university student

**Background information**

1. Gender. If you choose “other” then please specify under “Please enter your comment here:".

- Male
- Female
- Other:_______________________________________________________________

1. Please type in your age below.
2. What is the name of the university where you study?
3. What do you study?
4. Do you work? If so, please write down under "Please enter your comment here:" what kind of work you do.

- Yes:_______________________________________________________________
- No

1. Over the past three (3) months, how often in your leisure time have you been physically active (playing sports, doing gardening, high-speed cycling or brisk walking, etc.) for at least 30 minutes at a time so that you are slightly out of breath or sweating?

- Never
- Once a month or less
- 2-3 times per month
- Once a week
- 2-3 times a week
- 4-6 times a week
- Every day

**The consequences of being physically active**

1. In the following list are some of the consequences of physical activity. Please rate how likely these consequences are.

| 1. an injury or injuries | 1 (= not likely at all) ------------------------------------------ 7 (= very likely) |
| --- | --- |
| 1. having less time for other things | 1 (= not likely at all) ------------------------------------------ 7 (= very likely) |
| 1. feeling tired | 1 (= not likely at all) ------------------------------------------ 7 (= very likely) |
| 1. feelings of achievement | 1 (= not likely at all) ------------------------------------------ 7 (= very likely) |
| 1. better physical health | 1 (= not likely at all) ------------------------------------------ 7 (= very likely) |
| 1. better mental health | 1 (= not likely at all) ------------------------------------------ 7 (= very likely) |
| 1. a better appearance | 1 (= not likely at all) ------------------------------------------ 7 (= very likely) |
| 1. better sleep | 1 (= not likely at all) ------------------------------------------ 7 (= very likely) |
| 1. feeling good | 1 (= not likely at all) ------------------------------------------ 7 (= very likely) |
| 1. sweating | 1 (= not likely at all) ------------------------------------------ 7 (= very likely) |
| 1. feeling uncomfortable | 1 (= not likely at all) ------------------------------------------ 7 (= very likely) |
| 1. feeling pain | 1 (= not likely at all) ------------------------------------------ 7 (= very likely) |
| 1. feeling exhausted | 1 (= not likely at all) ------------------------------------------ 7 (= very likely) |
| 1. more energy | 1 (= not likely at all) ------------------------------------------ 7 (= very likely) |
| 1. better mood | 1 (= not likely at all) ------------------------------------------ 7 (= very likely) |
| 1. better overall health | 1 (= not likely at all) ------------------------------------------ 7 (= very likely) |
| 1. relieving stress | 1 (= not likely at all) ------------------------------------------ 7 (= very likely) |
| 1. maintaining health | 1 (= not likely at all) ------------------------------------------ 7 (= very likely) |
| 1. better focus | 1 (= not likely at all) ------------------------------------------ 7 (= very likely) |
| 1. less health worries | 1 (= not likely at all) ------------------------------------------ 7 (= very likely) |
| 1. an increase in physical strength | 1 (= not likely at all) ------------------------------------------ 7 (= very likely) |
| 1. the prevention of getting a disease/diseases | 1 (= not likely at all) ------------------------------------------ 7 (= very likely) |
| 1. better self-esteem | 1 (= not likely at all) ------------------------------------------ 7 (= very likely) |
| 1. muscle pain | 1 (= not likely at all) ------------------------------------------ 7 (= very likely) |
| 1. self-satisfaction | 1 (= not likely at all) ------------------------------------------ 7 (= very likely) |
| 1. rest from school/work | 1 (= not likely at all) ------------------------------------------ 7 (= very likely) |
| 1. staying in shape | 1 (= not likely at all) ------------------------------------------ 7 (= very likely) |
| 1. prevention of weight gain | 1 (= not likely at all) ------------------------------------------ 7 (= very likely) |
| 1. stable weight | 1 (= not likely at all) ------------------------------------------ 7 (= very likely) |
| 1. peace of mind | 1 (= not likely at all) ------------------------------------------ 7 (= very likely) |
| 1. better well-being | 1 (= not likely at all) ------------------------------------------ 7 (= very likely) |
| 1. better posture | 1 (= not likely at all) ------------------------------------------ 7 (= very likely) |
| 1. higher life expectancy | 1 (= not likely at all) ------------------------------------------ 7 (= very likely) |
| 1. higher productivity | 1 (= not likely at all) ------------------------------------------ 7 (= very likely) |

1. We would like to receive your feedback on the questions (in this case we are referring to "7. In the following list... etc.") that you answered (e.g., were the questions clear? Do you have any recommendations to improve the questions?).
2. How acceptable or unacceptable are the following possible outcomes of physical activity for you?

*Here “acceptable” means that it is OK and that you can allow this outcome to be there. While “unacceptable” means that it is not OK and you can’t allow the outcome to be there.*

| 1. Getting an injury or injuries | -3 (= not acceptable at all)-------------------------------3 (= very acceptable) |
| --- | --- |
| 1. Having less time for other things | -3 (= not acceptable at all)-------------------------------3 (= very acceptable) |
| 1. Feeling tired | -3 (= not acceptable at all)-------------------------------3 (= very acceptable) |
| 1. Sweating | -3 (= not acceptable at all)-------------------------------3 (= very acceptable) |
| 1. Feeling uncomfortable | -3 (= not acceptable at all)-------------------------------3 (= very acceptable) |
| 1. Feeling pain | -3 (= not acceptable at all)-------------------------------3 (= very acceptable) |
| 1. Feeling exhausted | -3 (= not acceptable at all)-------------------------------3 (= very acceptable) |
| 1. Muscle pain | -3 (= not acceptable at all)-------------------------------3 (= very acceptable) |

1. We would like to receive your feedback on the questions (in this case we are refering to "9. How acceptable or unacceptable... etc.) that you answered (e.g., were the questions clear? Do you have any recommendations to improve the questions?).

**Physical activity and others**

1. The following people think that I should be more physically active...

*If one or more of these people do not exist or you have no contact with them, please choose “no answer”. Also, if you don’t know what the other person thinks, please choose “no answer”.*

| 1. My mom | 1 (= not true at all) ---------------------------- 7 (= very true) |
| --- | --- |
| 1. My dad | 1 (= not true at all) ---------------------------- 7 (= very true) |
| 1. My brother(s) | 1 (= not true at all) ---------------------------- 7 (= very true) |
| 1. My sister(s) | 1 (= not true at all) ---------------------------- 7 (= very true) |
| 1. My friend(s) | 1 (= not true at all) ---------------------------- 7 (= very true) |
| 1. My colleague(s) | 1 (= not true at all) ---------------------------- 7 (= very true) |
| 1. Other students(s) | 1 (= not true at all) ---------------------------- 7 (= very true) |
| 1. My relative(s) (other than parents/siblings) | 1 (= not true at all) ---------------------------- 7 (= very true) |

1. The following people are physically active on a regular basis...

*If one or more of these people do not exist, please choose “no answer”. Also, if you don’t know if the other person is physically active on a regular basis, please choose “no answer”.*

| 1. My mom | 1 (= not true at all) ---------------------------- 7 (= very true) |
| --- | --- |
| 1. My dad | 1 (= not true at all) ---------------------------- 7 (= very true) |
| 1. My brother(s) | 1 (= not true at all) ---------------------------- 7 (= very true) |
| 1. My sister(s) | 1 (= not true at all) ---------------------------- 7 (= very true) |
| 1. My friend(s) | 1 (= not true at all) ---------------------------- 7 (= very true) |
| 1. My colleague(s) | 1 (= not true at all) ---------------------------- 7 (= very true) |
| 1. Other students(s) | 1 (= not true at all) ---------------------------- 7 (= very true) |
| 1. My relative(s) (other than parents/siblings) | 1. (= not true at all) ---------------------------- 7 (= very true) |

1. When talking about being physically active, how much do you care what the following people think you should do (e.g., if your mom tells you to be more physically active, how much do you care about her saying this?)

*If one or more of these people do not exist, please choose “no answer”. Also, if you believe that the other person does not think anything about what you should do, please choose “no answer”.*

| 1. My mom | 1 (= I don’t care at all) ------------------------ 7 (= I care a lot) |
| --- | --- |
| 1. My dad | 1 (= I don’t care at all) ------------------------ 7 (= I care a lot) |
| 1. My brother(s) | 1 (= I don’t care at all) ------------------------ 7 (= I care a lot) |
| 1. My sister(s) | 1 (= I don’t care at all) ------------------------ 7 (= I care a lot) |
| 1. My friend(s) | 1 (= I don’t care at all) ------------------------ 7 (= I care a lot) |
| 1. My colleague(s) | 1 (= I don’t care at all) ------------------------ 7 (= I care a lot) |
| 1. Other students | 1 (= I don’t care at all) ------------------------ 7 (= I care a lot) |
| 1. My relative(s) (other than parents/siblings) | 1 (= I don’t care at all) ------------------------ 7 (= I care a lot) |

1. We would like to receive your feedback on the questions (in this case we are refering to “11. The following people think… etc.”; “12. The following people are… etc.”; “13. When talking about… etc.”) that you answered (e.g., were the questions clear? Do you have any recommendations to improve the questions?).

**Different factors that influence physical activity levels**

1. The amount of time that I spend on being physically active depends on...

*If one or more of these options don’t apply to you, please choose "no answer".*

| 1. the weather | 1 (= not true at all) ---------------------------- 7 (= very true) |
| --- | --- |
| 1. whether I have the time for it or not | 1 (= not true at all) ---------------------------- 7 (= very true) |
| 1. how motivated I am | 1 (= not true at all) ---------------------------- 7 (= very true) |
| 1. my financial situation | 1 (= not true at all) ---------------------------- 7 (= very true) |
| 1. how I feel | 1 (= not true at all) ---------------------------- 7 (= very true) |
| 1. my energy levels | 1 (= not true at all) ---------------------------- 7 (= very true) |
| 1. where I live | 1 (= not true at all) ---------------------------- 7 (= very true) |
| 1. whether there is a gym nearby | 1 (= not true at all) ---------------------------- 7 (= very true) |
| 1. whether the gym is open or not | 1 (= not true at all) ---------------------------- 7 (= very true) |
| 1. whether I have someone to do it with or not ( | 1 (= not true at all) ---------------------------- 7 (= very true) |
| 1. my planning skills | 1 (= not true at all) ---------------------------- 7 (= very true) |
| 1. whether the environment is safe | 1 (= not true at all) ---------------------------- 7 (= very true) |
| 1. the amount of responsibilities that I have | 1 (= not true at all) ---------------------------- 7 (= very true) |
| 1. the amount of time I have to spend at work/school | 1 (= not true at all) ---------------------------- 7 (= very true) |
| 1. the amount of time I have to spend doing school/work related things | 1 (= not true at all) ---------------------------- 7 (= very true) |
| 1. whether I have a car or not (e.g., to go to the gym) | 1 (= not true at all) ---------------------------- 7 (= very true) |
| 1. my mood | 1 (= not true at all) ---------------------------- 7 (= very true) |
| 1. my stress levels | 1 (= not true at all) ---------------------------- 7 (= very true) |
| 1. having the necessary equipment | 1 (= not true at all) ---------------------------- 7 (= very true) |
| 1. whether people around me are physically active | 1 (= not true at all) ---------------------------- 7 (= very true) |
| 1. my children | 1 (= not true at all) ---------------------------- 7 (= very true) |
| 1. the Covid-19 situation | 1 (= not true at all) ---------------------------- 7 (= very true) |
| 1. my illness(es)/disease(s) | 1 (= not true at all) ---------------------------- 7 (= very true) |
| 1. how fast I see the results | 1 (= not true at all) ---------------------------- 7 (= very true) |
| 1. other priorities that I have | 1 (= not true at all) ---------------------------- 7 (= very true) |

16. Please answer the following:

| 1. The amount of time that I spend on being physically active is completely up to me. | 1 (= not true at all) ---------------------------- 7 (= very true) |
| --- | --- |
| 1. I am confident that I can be more physically active. | 1. (= not true at all) ---------------------------- 7 (= very true) |

1. We would like to receive your feedback on the questions (in this case we are refering to “15. The amount of time... etc.”; "16. Please answer the following:”) that you answered (e.g., were the questions clear? Do you have any recommendations to improve the questions?).

**The influence of different factors on physical activity levels**

18. How difficult do the following factors make it for you to be physically active?

*If one or more of these options don’t apply to you, please choose "no answer".*

| 1. Bad weather | 1 (= not difficult at all) ---------------------------- 7 (= very difficult) |
| --- | --- |
| 1. Not having the time | 1 (= not difficult at all) ---------------------------- 7 (= very difficult) |
| 1. Low motivation | 1 (= not difficult at all) ---------------------------- 7 (= very difficult) |
| 1. My financial situation | 1 (= not difficult at all) ---------------------------- 7 (= very difficult) |
| 1. Feeling bad | 1 (= not difficult at all) ---------------------------- 7 (= very difficult) |
| 1. Low energy levels | 1 (= not difficult at all) ---------------------------- 7 (= very difficult) |
| 1. The place where I live | 1 (= not difficult at all) ---------------------------- 7 (= very difficult) |
| 1. Not having a gym nearby | 1 (= not difficult at all) ---------------------------- 7 (= very difficult) |
| 1. When the gym is closed | 1 (= not difficult at all) ---------------------------- 7 (= very difficult) |
| 1. When I don't have anyone to do it with | 1 (= not difficult at all) ---------------------------- 7 (= very difficult) |
| 1. Poor planning skills | 1 (= not difficult at all) ---------------------------- 7 (= very difficult) |
| 1. Living in an unsafe environment | 1 (= not difficult at all) ---------------------------- 7 (= very difficult) |
| 1. Having lots of responsibilities | 1 (= not difficult at all) ---------------------------- 7 (= very difficult) |
| 1. Spending lots of time at work/school | 1 (= not difficult at all) ---------------------------- 7 (= very difficult) |
| 1. Spending lots of time doing work/school related things | 1 (= not difficult at all) ---------------------------- 7 (= very difficult) |
| 1. Not having a car | 1 (= not difficult at all) ---------------------------- 7 (= very difficult) |
| 1. Being in a bad mood | 1 (= not difficult at all) ---------------------------- 7 (= very difficult) |
| 1. Having high stress levels | 1 (= not difficult at all) ---------------------------- 7 (= very difficult) |
| 1. Not having the necessary equipment | 1 (= not difficult at all) ---------------------------- 7 (= very difficult) |
| 1. Not having people around me who are physical active | 1 (= not difficult at all) ---------------------------- 7 (= very difficult) |
| 1. My children | 1 (= not difficult at all) ---------------------------- 7 (= very difficult) |
| 1. The Covid-19 situation | 1 (= not difficult at all) ---------------------------- 7 (= very difficult) |
| 1. My illness(es)/disease(s) | 1 (= not difficult at all) ---------------------------- 7 (= very difficult) |
| 1. Not seeing the results fast enough | 1 (= not difficult at all) ---------------------------- 7 (= very difficult) |
| 1. Other priorities that I have | 1 (= not difficult at all) ---------------------------- 7 (= very difficult) |

1. We would like to receive your feedback on the questions (in this case we are refering to “18. How difficult do the following… etc.”) that you answered (e.g., were the questions clear? Do you have any recommendations to improve the questions?).

**Intentions**

20. Please answer the following

| 1. I want to be more physically active | 1 (= not true at all) ---------------------------- 7 (= very true) |
| --- | --- |
| 1. My intention is to be more physically active | 1 (= not true at all) ---------------------------- 7 (= very true) |

1. We would like to receive your feedback on the questions (in this case we are refering to "20. Please answer the following:") that you answered (e.g., were the questions clear? Do you have any recommendations to improve the questions?).

**Questionnaire for second phase in Estonian**

Tere, tänan sind, et leidsid aega selle küsimustiku täitmiseks. Alguses on mõned küsimused sinu tausta kohta ja pärast seda esitame sulle küsimusi, mis on seotud kehalise aktiivsusega. Küsimustiku jooksul palume sul hinnata vastatud küsimusi (nt "kas küsimused olid selged" ja "kas sul on soovitusi küsimuste parandamiseks?"). Palume küsimuste hindamisel olla võimalikult kriitilised. See aitab meil küsimustikku täiustada. Küsimustiku täitmine ei kesta kauem kui 20 minutit. Täida küsimustik ainult siis, kui oled täiskoormusega ülikooli üliõpilane.

**Taustainfo**

1. Sugu. Kui valid "muu”, täpsustage palun alla "Palun lisa oma kommentaar siia:”

- Mees
- Naine
- Muu:_______________________________________________________________

1. Palun sisestage allpool oma vanus.
2. Mis on ülikooli nimi, kus sa õpid?
3. Mida sa ōpid?
4. Kas sa töötad? Kui jah, kirjutage palun alla "Palun lisa oma kommentaar siia:" millist tööd teed.

- Jah:_______________________________________________________________
- Ei

1. Kui sageli oled sa viimase kolme (3) kuu jooksul tegelenud vabal ajal aktiivse liikumisega (nt tervisesport, aiatöö, kiires tempos jalgrattasõit või kõndimine) vähemalt poole tunni vältel korraga, nii et hakkad kergelt hingeldama või higistama?

- Mitte kordagi
- Umbes kord kuus või harvem
- 2-3 korda kuus
- Kord nädalas
- 2-3 korda nädalas
- 4-6 korda nädalas
- Iga päev

**Füüsilise aktiivsuse tagajärjed**

1. Järgnevalt on loetletud mõned võimalikud kehalise aktiivsuse tagajärjed. Palun hinda igal real, kui tõenäoliseks sa neid tagajärgi pead.

| 1. Vigastus vōi vigastud | 1 (= pole üldse tõenäoline) --------------------------- 7 (= väga tõenäoline) |
| --- | --- |
| 1. muude asjade jaoks vähem aega | 1 (= pole üldse tõenäoline) --------------------------- 7 (= väga tõenäoline) |
| 1. väsimustunne | 1 (= pole üldse tõenäoline) --------------------------- 7 (= väga tõenäoline) |
| 1. tunne, et sa oled midagi saavutanud | 1 (= pole üldse tõenäoline) --------------------------- 7 (= väga tõenäoline) |
| 1. parem füüsiline tervis | 1 (= pole üldse tõenäoline) --------------------------- 7 (= väga tõenäoline) |
| 1. parem vaimne tervis | 1 (= pole üldse tõenäoline) --------------------------- 7 (= väga tõenäoline) |
| 1. parem välimus | 1 (= pole üldse tõenäoline) --------------------------- 7 (= väga tõenäoline) |
| 1. parem uni | 1 (= pole üldse tõenäoline) --------------------------- 7 (= väga tõenäoline) |
| 1. hea enesetunne | 1 (= pole üldse tõenäoline) --------------------------- 7 (= väga tõenäoline) |
| 1. higistamine | 1 (= pole üldse tõenäoline) --------------------------- 7 (= väga tõenäoline) |
| 1. ebamugavustunne | 1 (= pole üldse tõenäoline) --------------------------- 7 (= väga tõenäoline) |
| 1. valu tundmine | 1 (= pole üldse tõenäoline) --------------------------- 7 (= väga tõenäoline) |
| 1. kurnatuse tunne | 1 (= pole üldse tõenäoline) --------------------------- 7 (= väga tõenäoline) |
| 1. rohkem energiat | 1 (= pole üldse tõenäoline) --------------------------- 7 (= väga tõenäoline) |
| 1. parem tuju | 1 (= pole üldse tõenäoline) --------------------------- 7 (= väga tõenäoline) |
| 1. parem üldine tervis | 1 (= pole üldse tõenäoline) --------------------------- 7 (= väga tõenäoline) |
| 1. stressi maandamine | 1 (= pole üldse tõenäoline) --------------------------- 7 (= väga tõenäoline) |
| 1. tervise säilitamine | 1 (= pole üldse tõenäoline) --------------------------- 7 (= väga tõenäoline) |
| 1. parem keskendusvōime | 1 (= pole üldse tõenäoline) --------------------------- 7 (= väga tõenäoline) |
| 1. vähem tervisemuresid | 1 (= pole üldse tõenäoline) --------------------------- 7 (= väga tõenäoline) |
| 1. füüsilise jōu tōus | 1 (= pole üldse tõenäoline) --------------------------- 7 (= väga tõenäoline) |
| 1. terviseprobleemide ennetamine | 1 (= pole üldse tõenäoline) --------------------------- 7 (= väga tõenäoline) |
| 1. parem enesehinnang | 1 (= pole üldse tõenäoline) --------------------------- 7 (= väga tõenäoline) |
| 1. lihasvalu | 1 (= pole üldse tõenäoline) --------------------------- 7 (= väga tõenäoline) |
| 1. enesega rahulolu | 1 (= pole üldse tõenäoline) --------------------------- 7 (= väga tõenäoline) |
| 1. puhata koolist/tööst | 1 (= pole üldse tõenäoline) --------------------------- 7 (= väga tõenäoline) |
| 1. vormis püsimine | 1 (= pole üldse tõenäoline) --------------------------- 7 (= väga tõenäoline) |
| 1. kaalutōusu vältimine | 1 (= pole üldse tõenäoline) --------------------------- 7 (= väga tõenäoline) |
| 1. stabiilne kaal | 1 (= pole üldse tõenäoline) --------------------------- 7 (= väga tõenäoline) |
| 1. meelerahu | 1 (= pole üldse tõenäoline) --------------------------- 7 (= väga tõenäoline) |
| 1. parem enesetunne | 1 (= pole üldse tõenäoline) --------------------------- 7 (= väga tõenäoline) |
| 1. parem rüht | 1 (= pole üldse tõenäoline) --------------------------- 7 (= väga tõenäoline) |
| 1. Pikem eluiga | 1 (= pole üldse tõenäoline) --------------------------- 7 (= väga tõenäoline) |
| 1. Suurem töövõime | 1 (= pole üldse tõenäoline) --------------------------- 7 (= väga tõenäoline) |

1. Palun kommenteeri eelnevaid küsimusi ("7. Järgnevalt on loetletud... jne."). Kas küsimused olid sinu jaoks selged? Kas sinu meelest oleks vaja küsimusi muuta või parandada?
2. Kui vastuvõetavad või vastuvõetamatud on sinu jaoks järgmised võimalikud kehalise aktiivsuse tulemused?

*Siin tähendab "vastuvõetav", et see on sinu jaoks aktsepteeritav ja võid lasta sel juhtuda. Samas "vastuvõetamatu" tähendab, et see pole sinu jaoks aktsepteeritav ja sulle ei sobi, et selline asi juhtub.*

| 1. Vigastus(ed) | -3 (= pole üldse valmis vastuvōetav)-----------3 (= väga vastuvōetav) |
| --- | --- |
| 1. Vähem aega muude asjade jaoks | -3 (= pole üldse valmis vastuvōetav)-----------3 (= väga vastuvōetav) |
| 1. Väsimustunne | -3 (= pole üldse valmis vastuvōetav)-----------3 (= väga vastuvōetav) |
| 1. Higistamine | -3 (= pole üldse valmis vastuvōetav)-----------3 (= väga vastuvōetav) |
| 1. Ebamugavustunne | -3 (= pole üldse valmis vastuvōetav)-----------3 (= väga vastuvōetav) |
| 1. Valutunne | -3 (= pole üldse valmis vastuvōetav)-----------3 (= väga vastuvōetav) |
| 1. Väsimustunne | -3 (= pole üldse valmis vastuvōetav)-----------3 (= väga vastuvōetav) |
| 1. Lihasvalu | -3 (= pole üldse valmis vastuvōetav)-----------3 (= väga vastuvōetav) |

1. Palun kommenteeri eelnevaid küsimusi (9. "Kui vastuvõetavad ..... jne"). Kas küsimused olid sinu jaoks selged? Kas sinu meelest oleks vaja küsimusi muuta või parandada?

**Füüsiline aktiivsus ja teised inimesed**

1. Järgnevad inimesed arvavad, et peaksin olema kehaliselt aktiivsem ...

*Kui ühte või mitut neist inimestest pole olemas või sa ei ole nendega kontaktis, siis valige "Vastust pole”. Samuti, kui te ei tea, mida teine inimene arvab, valige "Vastust pole".*

| 1. Minu ema | 1 (= pole üldse tōsi) --------------------------- 7 (= väga tōsi) |
| --- | --- |
| 1. Minu isa | 1 (= pole üldse tōsi) --------------------------- 7 (= väga tōsi) |
| 1. Minu vend/vennad | 1 (= pole üldse tōsi) --------------------------- 7 (= väga tōsi) |
| 1. Minu ōde/ōed | 1 (= pole üldse tōsi) --------------------------- 7 (= väga tōsi) |
| 1. Minu sõber/sōbrad | 1 (= pole üldse tōsi) --------------------------- 7 (= väga tōsi) |
| 1. Minu kolleeg(id) | 1 (= pole üldse tōsi) --------------------------- 7 (= väga tōsi) |
| 1. Teised ōpilased | 1 (= pole üldse tōsi) --------------------------- 7 (= väga tōsi) |
| 1. Sugulased (v.a vanemad ja ōed-vennad) | 1 (= pole üldse tōsi) --------------------------- 7 (= väga tōsi) |

1. Järgmised inimesed on regulaarselt kehaliselt aktiivsed ...

*Kui ühte või mitut neist inimestest ei eksisteeri, valige "Vastust pole". Samuti, kui te ei tea, kas teine inimene on regulaarselt füüsiliselt aktiivne, valige "Vastust pole".*

| 1. Minu ema | 1 (= pole üldse tōsi) --------------------------- 7 (= väga tōsi) |
| --- | --- |
| 1. Minu isa | 1 (= pole üldse tōsi) --------------------------- 7 (= väga tōsi) |
| 1. Minu vend/vennad | 1 (= pole üldse tōsi) --------------------------- 7 (= väga tōsi) |
| 1. Minu ōde/ōed | 1 (= pole üldse tōsi) --------------------------- 7 (= väga tōsi) |
| 1. Minu sõber/sōbrad | 1 (= pole üldse tōsi) --------------------------- 7 (= väga tōsi) |
| 1. Minu kolleeg(id) | 1 (= pole üldse tōsi) --------------------------- 7 (= väga tōsi) |
| 1. Teised ōpilased | 1 (= pole üldse tōsi) --------------------------- 7 (= väga tōsi) |
| 1. Sugulased (v.a vanemad ja ōed-vennad) | 1 (= pole üldse tōsi) --------------------------- 7 (= väga tōsi) |

1. Millisel määral sulle läheb korda järgmiste inimeste arvamus sinu kehalise aktiivsuse kohta? (nt kui sinu ema ütleb sulle, et sa peaksid olema kehaliselt aktiivsem, siis kui palju tema arvamus sulle korda läheb?)

*Kui ühte või mitut neist inimestest ei eksisteeri, valige “Vastust pole“. Samuti, kui arvad, et teine inimene ei mõtle midagi, mida peaksid tegema, valige palun "Vastust pole”.*

| 1. Minu ema | 1 (= ei hooli üldse) ------------------------- 7 (= ma hoolin väga) |
| --- | --- |
| 1. Minu isa | 1 (= ei hooli üldse) ------------------------- 7 (= ma hoolin väga) |
| 1. Minu vend/vennad | 1 (= ei hooli üldse) ------------------------- 7 (= ma hoolin väga) |
| 1. Minu ōde/ōed | 1 (= ei hooli üldse) ------------------------- 7 (= ma hoolin väga) |
| 1. Minu sõber/sōbrad | 1 (= ei hooli üldse) ------------------------- 7 (= ma hoolin väga) |
| 1. Minu kolleeg(id) | 1 (= ei hooli üldse) ------------------------- 7 (= ma hoolin väga) |
| 1. Teised (üli)ōpilased | 1 (= ei hooli üldse) ------------------------- 7 (= ma hoolin väga) |
| 1. Minu elukaaslane/partner | 1 (= ei hooli üldse) ------------------------- 7 (= ma hoolin väga) |
| 1. Minu laps(ed) | 1 (= ei hooli üldse) ------------------------- 7 (= ma hoolin väga) |
| 1. Sugulased (v.a vanemad, ōed-vennad ja lapsed) | 1 (= ei hooli üldse) ------------------------- 7 (= ma hoolin väga) |

1. Palun kommenteeri eelnevaid küsimusi (11. "Järgnevad inimesed arvavad ..... jne”; “12. Järgmised inimesed on regulaarselt… jne.”; “13. Millisel määral sulle läheb… jne.”). Kas küsimused olid sinu jaoks selged? Kas sinu meelest oleks vaja küsimusi muuta või parandada?

**Erinevad tegurid, mis mõjutavad füüsilist aktiivsust**

1. Kehaliselt aktiivseks olemiseks kuluv aeg sõltub sellest...

*Kui üks või mitu neist valikutest ei kehti sinu kohta, valige "vastust pole".*

| 1. Ilmast | 1 (= pole üldse tōsi) --------------------7 (= väga tōsi) |
| --- | --- |
| 1. kas mul on aega või mitte | 1 (= pole üldse tōsi) --------------------7 (= väga tōsi) |
| 1. kui motiveeritud ma olen | 1 (= pole üldse tōsi) --------------------7 (= väga tōsi) |
| 1. minu majanduslikust olukorrast | 1 (= pole üldse tōsi) --------------------7 (= väga tōsi) |
| 1. kuidas ma end tunnen | 1 (= pole üldse tōsi) --------------------7 (= väga tōsi) |
| 1. minu energiatasemest | 1 (= pole üldse tōsi) --------------------7 (= väga tōsi) |
| 1. kus ma elan | 1 (= pole üldse tōsi) --------------------7 (= väga tōsi) |
| 1. kas läheduses on jōusaal | 1 (= pole üldse tōsi) --------------------7 (= väga tōsi) |
| 1. kas jõusaal on avatud või mitte | 1 (= pole üldse tōsi) --------------------7 (= väga tōsi) |
| 1. kas mul on kellegagi seda teha või mitte | 1 (= pole üldse tōsi) --------------------7 (= väga tōsi) |
| 1. minu planeerimisoskusest | 1 (= pole üldse tōsi) --------------------7 (= väga tōsi) |
| 1. kas keskkond on ohutu | 1 (= pole üldse tōsi) --------------------7 (= väga tōsi) |
| 1. kohustuste mahust, mis mul on | 1 (= pole üldse tōsi) --------------------7 (= väga tōsi) |
| 1. kui palju aega pean tööl/koolis veetma | 1 (= pole üldse tōsi) --------------------7 (= väga tōsi) |
| 1. kui palju aega pean kulutama kooli/tööga seotud asjade tegemisele | 1 (= pole üldse tōsi) --------------------7 (= väga tōsi) |
| 1. kas mul on auto vōi mitte (nt jōusaali minna) | 1 (= pole üldse tōsi) --------------------7 (= väga tōsi) |
| 1. minu tujust | 1 (= pole üldse tōsi) --------------------7 (= väga tōsi) |
| 1. minu stressitasemest | 1 (= pole üldse tōsi) --------------------7 (= väga tōsi) |
| 1. vajalike seadmete olemasolust | 1 (= pole üldse tōsi) --------------------7 (= väga tōsi) |
| 1. kas inimesed minu ümber on füüsiliselt aktiivsed | 1 (= pole üldse tōsi) --------------------7 (= väga tōsi) |
| 1. mu lapstest | 1 (= pole üldse tōsi) --------------------7 (= väga tōsi) |
| 1. Covid-19 olukorrast | 1 (= pole üldse tōsi) --------------------7 (= väga tōsi) |
| 1. minu tervisest | 1 (= pole üldse tōsi) --------------------7 (= väga tōsi) |
| 1. kui kiiresti ma tulemusi näen | 1 (= pole üldse tōsi) --------------------7 (= väga tōsi) |
| 1. minu teisest eesmärkidest | 1 (= pole üldse tōsi) --------------------7 (= väga tōsi) |

16. Palun vastake järgmisetele väidetele:

| 1. Aeg, mille kulutan kehaliselt aktiivsele tegevusele, sõltub täielikult minust. | 1 (= pole üldse tōsi) -------------------7 (= väga ōige) |
| --- | --- |
| 1. Olen kindel, et ma saan oma kehalist aktiivsust suurendada. | 1. (= pole üldse tōsi) -------------------7 (= väga ōige) |

1. Palun kommenteeri eelnevaid küsimusi (“15. Kehaliselt aktiivseks olemiseks ..... jne.” ja “16. Palun vastake… jne.“). Kas küsimused olid sinu jaoks selged? Kas sinu meelest oleks vaja küsimusi muuta või parandada?

**Erinevate tegurite mõju füüsilise aktiivsuse tasemele**

1. Mil määral teevad järgmised tegurid kehalise aktiivsuse sinu jaoks raskeks?

*Kui üks või mitu neist valikutest ei kehti sinu kohta, valige "vastust pole".*

| 1. Halb ilm | 1 (= üldse mitte) -------------------7 (= väga suurel maaral) |
| --- | --- |
| 1. Pole aega | 1 (= üldse mitte) -------------------7 (= väga suurel maaral) |
| 1. Madal motivatsioon | 1 (= üldse mitte) -------------------7 (= väga suurel maaral) |
| 1. Minu majanduslik olukord | 1 (= üldse mitte) -------------------7 (= väga suurel maaral) |
| 1. halb enesetunne | 1 (= üldse mitte) -------------------7 (= väga suurel maaral) |
| 1. Madal energiatase | 1 (= üldse mitte) -------------------7 (= väga suurel maaral) |
| 1. Koht, kus ma elan | 1 (= üldse mitte) -------------------7 (= väga suurel maaral) |
| 1. Läheduses pole jōusaali | 1 (= üldse mitte) -------------------7 (= väga suurel maaral) |
| 1. Jõusaal on suletud | 1 (= üldse mitte) -------------------7 (= väga suurel maaral) |
| 1. Kui mul pole kellegagi seda teha | 1 (= üldse mitte) -------------------7 (= väga suurel maaral) |
| 1. Halb planeerimisoskus | 1 (= üldse mitte) -------------------7 (= väga suurel maaral) |
| 1. Elamine ebaturvalises keskkonnas | 1 (= üldse mitte) -------------------7 (= väga suurel maaral) |
| 1. Omades palju kohutusi | 1 (= üldse mitte) -------------------7 (= väga suurel maaral) |
| 1. Palju aega tööl/koolis | 1 (= üldse mitte) -------------------7 (= väga suurel maaral) |
| 1. Kulutada palju aega tööga/kooliga seotud asjadele | 1 (= üldse mitte) -------------------7 (= väga suurel maaral) |
| 1. Auto puudumine | 1 (= üldse mitte) -------------------7 (= väga suurel maaral) |
| 1. Olles halvas tujus | 1 (= üldse mitte) -------------------7 (= väga suurel maaral) |
| 1. Kōrge stressitase | 1 (= üldse mitte) -------------------7 (= väga suurel maaral) |
| 1. Vajalike seadmete puudumine | 1 (= üldse mitte) -------------------7 (= väga suurel maaral) |
| 1. Kui mu ümber pole inimesi, kes on füüsiliselt aktiivsed | 1 (= üldse mitte) -------------------7 (= väga suurel maaral) |
| 1. Minu lapsed | 1 (= üldse mitte) -------------------7 (= väga suurel maaral) |
| 1. Covid-19 olukord | 1 (= üldse mitte) -------------------7 (= väga suurel maaral) |
| 1. Minu haigus(ed) | 1 (= üldse mitte) -------------------7 (= väga suurel maaral) |
| 1. Ei näe tulemusi piisavalt kiiresti | 1 (= üldse mitte) -------------------7 (= väga suurel maaral) |
| 1. Muud prioriteedid, mis mul on | 1. (= üldse mitte) -------------------7 (= väga suurel maaral) |

1. Palun kommenteeri eelnevaid küsimusi (“18. Mil määral teevad järgmised… jne.”). Kas küsimused olid sinu jaoks selged? Kas sinu meelest oleks vaja küsimusi muuta või parandada?

**Kavatsused**

20. Palun vastake järgmisetele väidetele:

| 1. Tahan olla kehaliselt aktiivsem. | 1 (= pole üldse tōsi) ------------------------------------7 (= väga tõsi) |
| --- | --- |
| 1. Minu eesmärk on olla kehaliselt aktiivsem. | 1 (= pole üldse tōsi) ------------------------------------7 (= väga tõsi) |

1. Palun kommenteeri eelnevaid küsimusi (“20. Palun vastake järgmistele… jne.”). Kas küsimused olid sinu jaoks selged? Kas sinu meelest oleks vaja küsimusi muuta või parandada?
