# Supplementary material for: Toward Comprehensive Assessment of Beliefs and Attitudes Related to Physical Activity in Young Adults: Pilot Study
Source: JMIR Form Res. 2025 Oct 16;9:e69094. doi: 10.2196/69094 (PMC12576300; doi:10.2196/69094)
Supplement: Multimedia Appendix 3 [file formative_v9i1e69094_app3.docx]

**Questionnaire for third phase in English**

Hi, thank you for taking the time to fill in this questionnaire. In the beginning there will be some questions about your background and after that we will ask you questions related to being physically active. The main purpose of the questionnaire is to assess what you think are the consequences of physical activity, how other people perceive your physical activity levels and what factors affect your level of physical activity. In total there are 15 questions. Filling in the questionnaire should not take more than 15 minutes. Only fill in the questionnaire if you are a full-time university student.

**Background information**

1. Gender. If you choose “other” then please specify under “Please enter your comment here:".

- Male
- Female
- Other:_______________________________________________________________

1. Please type in your age below.
2. What is the name of the university where you study?
3. What do you study?
4. Do you work? If so, please write down under "Please enter your comment here:" what kind of work you do.

- Yes:_______________________________________________________________
- No

1. Over the past three (3) months, how often in your leisure time have you been physically active (playing sports, doing gardening, high-speed cycling or brisk walking, etc.) for at least 30 minutes at a time so that you are slightly out of breath or sweating?

- Never
- Once a month or less
- 2-3 times per month
- Once a week
- 2-3 times a week
- 4-6 times a week
- Every day

**The consequences of being physically active**

1. In the following list are some of the physical and mental consequences of physical activity (e.g., sports, gardening, cycling or walking). Please rate how likely these consequences are for yourself. These consequences can occur during physical activity as well as after physical activity.

| 1. Injury/injuries | 1 (= not likely at all) ------------------------------------------ 7 (= very likely) |
| --- | --- |
| 1. Less time for other activities | 1 (= not likely at all) ------------------------------------------ 7 (= very likely) |
| 1. Feeling tired | 1 (= not likely at all) ------------------------------------------ 7 (= very likely) |
| 1. Feelings like you have accomplished something | 1 (= not likely at all) ------------------------------------------ 7 (= very likely) |
| 1. Better physical health | 1 (= not likely at all) ------------------------------------------ 7 (= very likely) |
| 1. Better mental health | 1 (= not likely at all) ------------------------------------------ 7 (= very likely) |
| 1. Better appearance | 1 (= not likely at all) ------------------------------------------ 7 (= very likely) |
| 1. Better sleep | 1 (= not likely at all) ------------------------------------------ 7 (= very likely) |
| 1. Feeling good | 1 (= not likely at all) ------------------------------------------ 7 (= very likely) |
| 1. Sweating | 1 (= not likely at all) ------------------------------------------ 7 (= very likely) |
| 1. Feeling uncomfortable | 1 (= not likely at all) ------------------------------------------ 7 (= very likely) |
| 1. Feeling pain (not a normal "painful" feeling of muscle tension/pain) | 1 (= not likely at all) ------------------------------------------ 7 (= very likely) |
| 1. Feeling exhausted | 1 (= not likely at all) ------------------------------------------ 7 (= very likely) |
| 1. More energy | 1 (= not likely at all) ------------------------------------------ 7 (= very likely) |
| 1. Better mood | 1 (= not likely at all) ------------------------------------------ 7 (= very likely) |
| 1. Better overall health | 1 (= not likely at all) ------------------------------------------ 7 (= very likely) |
| 1. Decreasing stress | 1 (= not likely at all) ------------------------------------------ 7 (= very likely) |
| 1. Maintaining health | 1 (= not likely at all) ------------------------------------------ 7 (= very likely) |
| 1. Better focus | 1 (= not likely at all) ------------------------------------------ 7 (= very likely) |
| 1. Less health worries | 1 (= not likely at all) ------------------------------------------ 7 (= very likely) |
| 1. An increase in physical strength | 1 (= not likely at all) ------------------------------------------ 7 (= very likely) |
| 1. Preventing health problems | 1 (= not likely at all) ------------------------------------------ 7 (= very likely) |
| 1. Better self-esteem | 1 (= not likely at all) ------------------------------------------ 7 (= very likely) |
| 1. Muscle pain | 1 (= not likely at all) ------------------------------------------ 7 (= very likely) |
| 1. Self-satisfaction | 1 (= not likely at all) ------------------------------------------ 7 (= very likely) |
| 1. Resting from school/work | 1 (= not likely at all) ------------------------------------------ 7 (= very likely) |
| 1. Staying in shape | 1 (= not likely at all) ------------------------------------------ 7 (= very likely) |
| 1. Preventing weight gain | 1 (= not likely at all) ------------------------------------------ 7 (= very likely) |
| 1. Weight gain (e.g., muscle mass) | 1 (= not likely at all) ------------------------------------------ 7 (= very likely) |
| 1. Stable weight | 1 (= not likely at all) ------------------------------------------ 7 (= very likely) |
| 1. Peace of mind | 1 (= not likely at all) ------------------------------------------ 7 (= very likely) |
| 1. Feeling better | 1 (= not likely at all) ------------------------------------------ 7 (= very likely) |
| 1. Better posture | 1 (= not likely at all) ------------------------------------------ 7 (= very likely) |
| 1. Longer lifespan | 1 (= not likely at all) ------------------------------------------ 7 (= very likely) |
| 1. Greater work capacity | 1 (= not likely at all) ------------------------------------------ 7 (= very likely) |

1. To what extent are you prepared to accept the following consequences of physical activity (-3 not at all willing to accept; 3 I am completely willing to accept)?

*Here “willing to accept” means that it is OK and that you can allow this outcome to be there. While “not willing to accept” means that it is not OK and you can’t allow the outcome to be there.*

| 1. Injury/injuries | -3 (= not at all willing to accept)--3 (= I am completely willing to accept) |
| --- | --- |
| 1. Less time for other activities (e.g., work, school, other responsibilities) | -3 (= not at all willing to accept)--3 (= I am completely willing to accept) |
| 1. Feeling tired | -3 (= not at all willing to accept)--3 (= I am completely willing to accept) |
| 1. Sweating | -3 (= not at all willing to accept)--3 (= I am completely willing to accept) |
| 1. Feeling uncomfortable | -3 (= not at all willing to accept)--3 (= I am completely willing to accept) |
| 1. Feeling pain (not a normal "painful" feeling of muscle tension/pain) | -3 (= not at all willing to accept)--3 (= I am completely willing to accept) |
| 1. Feeling exhausted | -3 (= not at all willing to accept)--3 (= I am completely willing to accept) |
| 1. Muscle pain | -3 (= not at all willing to accept)--3 (= I am completely willing to accept) |

**Physical activity and others**

1. The following people think that I should be more physically active...

*If one or more of these people do not exist or you have no contact with them, please choose “no answer”. Also, if you don’t know what the other person thinks, please choose “no answer”.*

| 1. My mother | 1 (= not true at all) ---------------------------- 7 (= very true) |
| --- | --- |
| 1. My father | 1 (= not true at all) ---------------------------- 7 (= very true) |
| 1. My brother(s) | 1 (= not true at all) ---------------------------- 7 (= very true) |
| 1. My sister(s) | 1 (= not true at all) ---------------------------- 7 (= very true) |
| 1. My friend(s) | 1 (= not true at all) ---------------------------- 7 (= very true) |
| 1. My colleague(s) | 1 (= not true at all) ---------------------------- 7 (= very true) |
| 1. Other (university)students | 1 (= not true at all) ---------------------------- 7 (= very true) |
| 1. My partner | 1 (= not true at all) ---------------------------- 7 (= very true) |
| 1. My child(ren) | 1 (= not true at all) ---------------------------- 7 (= very true) |
| 1. Relatives (other than parents/siblings/children) | 1 (= not true at all) ---------------------------- 7 (= very true) |

1. The following people are physically active on a regular basis...

*If one or more of these people do not exist, please choose “no answer”. Also, if you don’t know if the other person is physically active on a regular basis, please choose “no answer”.*

| 1. My mother | 1 (= not true at all) ---------------------------- 7 (= very true) |
| --- | --- |
| 1. My father | 1 (= not true at all) ---------------------------- 7 (= very true) |
| 1. My brother(s) | 1 (= not true at all) ---------------------------- 7 (= very true) |
| 1. My sister(s) | 1 (= not true at all) ---------------------------- 7 (= very true) |
| 1. My friend(s) | 1 (= not true at all) ---------------------------- 7 (= very true) |
| 1. My colleague(s) | 1 (= not true at all) ---------------------------- 7 (= very true) |
| 1. Other (university)students | 1 (= not true at all) ---------------------------- 7 (= very true) |
| 1. My partner | 1 (= not true at all) ---------------------------- 7 (= very true) |
| 1. My child(ren) | 1 (= not true at all) ---------------------------- 7 (= very true) |
| 1. Relatives (other than parents/siblings/children) | 1 (= not true at all) ---------------------------- 7 (= very true) |

1. When talking about being physically active, how much do you care what the following people think you should do (e.g., if your mom tells you to be more physically active, how much do you care about her saying this?)

*If one or more of these people do not exist, please choose “no answer”. Also, if you believe that the other person does not think anything about what you should do, please choose “no answer”.*

| 1. My mother | 1 (= I don’t care at all) ------------------------ 7 (= I care a lot) |
| --- | --- |
| 1. My father | 1 (= I don’t care at all) ------------------------ 7 (= I care a lot) |
| 1. My brother(s) | 1 (= I don’t care at all) ------------------------ 7 (= I care a lot) |
| 1. My sister(s) | 1 (= I don’t care at all) ------------------------ 7 (= I care a lot) |
| 1. My friend(s) | 1 (= I don’t care at all) ------------------------ 7 (= I care a lot) |
| 1. My colleague(s) | 1 (= I don’t care at all) ------------------------ 7 (= I care a lot) |
| 1. Other (university)students | 1 (= I don’t care at all) ------------------------ 7 (= I care a lot) |
| 1. My partner | 1 (= I don’t care at all) ------------------------ 7 (= I care a lot) |
| 1. My child(ren) | 1 (= I don’t care at all) ------------------------ 7 (= I care a lot) |
| 1. Relatives (other than parents/siblings/children) | 1 (= I don’t care at all) ------------------------ 7 (= I care a lot) |

**Different factors that influence physical activity levels**

1. The amount of time that I spend on being physically active depends on...

*If one or more of these options don’t apply to you, please choose "no answer".*

| 1. The weather | 1 (= not true at all) ---------------------------- 7 (= very true) |
| --- | --- |
| 1. Whether I have time or not | 1 (= not true at all) ---------------------------- 7 (= very true) |
| 1. How motivated I am | 1 (= not true at all) ---------------------------- 7 (= very true) |
| 1. My financial situation | 1 (= not true at all) ---------------------------- 7 (= very true) |
| 1. How I am feeling | 1 (= not true at all) ---------------------------- 7 (= very true) |
| 1. My energy levels | 1 (= not true at all) ---------------------------- 7 (= very true) |
| 1. Where I live | 1 (= not true at all) ---------------------------- 7 (= very true) |
| 1. Whether there is a gym nearby | 1 (= not true at all) ---------------------------- 7 (= very true) |
| 1. Whether the gym is open or not | 1 (= not true at all) ---------------------------- 7 (= very true) |
| 1. Whether I have someone to do it with or not (e.g., play sports, exercise with someone, etc.) | 1 (= not true at all) ---------------------------- 7 (= very true) |
| 1. My planning skills | 1 (= not true at all) ---------------------------- 7 (= very true) |
| 1. Whether the environment is safe | 1 (= not true at all) ---------------------------- 7 (= very true) |
| 1. The amount of obligations that I have | 1 (= not true at all) ---------------------------- 7 (= very true) |
| 1. How many work or school commitments I have | 1 (= not true at all) ---------------------------- 7 (= very true) |
| 1. Whether I have a car or not (e.g., to go to the gym) | 1 (= not true at all) ---------------------------- 7 (= very true) |
| 1. My mood | 1 (= not true at all) ---------------------------- 7 (= very true) |
| 1. My stress levels | 1 (= not true at all) ---------------------------- 7 (= very true) |
| 1. Having the necessary equipment | 1 (= not true at all) ---------------------------- 7 (= very true) |
| 1. Whether people around me are physically active | 1 (= not true at all) ---------------------------- 7 (= very true) |
| 1. My children | 1 (= not true at all) ---------------------------- 7 (= very true) |
| 1. The Covid-19 situation | 1 (= not true at all) ---------------------------- 7 (= very true) |
| 1. My health | 1 (= not true at all) ---------------------------- 7 (= very true) |
| 1. How fast I see the results | 1 (= not true at all) ---------------------------- 7 (= very true) |
| 1. My other goals | 1 (= not true at all) ---------------------------- 7 (= very true) |

13. Please answer the following:

| 1. The amount of time that I spend on being physically active is completely up to me. | 1 (= not true at all) ---------------------------- 7 (= very true) |
| --- | --- |
| 1. I am confident that I can be more physically active. | 1 (= not true at all) ---------------------------- 7 (= very true) |

**The influence of different factors on physical activity levels**

14. How difficult do the following factors make it for you to be physically active?

*If one or more of these options don’t apply to you, please choose "no answer".*

| 1. Bad weather | 1 (= not difficult at all) ---------------------------- 7 (= very difficult) |
| --- | --- |
| 1. Not having time | 1 (= not difficult at all) ---------------------------- 7 (= very difficult) |
| 1. Low motivation | 1 (= not difficult at all) ---------------------------- 7 (= very difficult) |
| 1. My financial situation | 1 (= not difficult at all) ---------------------------- 7 (= very difficult) |
| 1. Feeling bad | 1 (= not difficult at all) ---------------------------- 7 (= very difficult) |
| 1. Low energy levels | 1 (= not difficult at all) ---------------------------- 7 (= very difficult) |
| 1. The place where I live | 1 (= not difficult at all) ---------------------------- 7 (= very difficult) |
| 1. There is no gym nearby | 1 (= not difficult at all) ---------------------------- 7 (= very difficult) |
| 1. The gym (or other sports facility) is closed | 1 (= not difficult at all) ---------------------------- 7 (= very difficult) |
| 1. When I don't have anyone to do it with (e.g., play sports, exercise with someone, etc.) | 1 (= not difficult at all) ---------------------------- 7 (= very difficult) |
| 1. My planning skills | 1 (= not difficult at all) ---------------------------- 7 (= very difficult) |
| 1. Living in an unsafe environment | 1 (= not difficult at all) ---------------------------- 7 (= very difficult) |
| 1. Having many obligations | 1 (= not difficult at all) ---------------------------- 7 (= very difficult) |
| 1. A lot of work or school related obligations | 1 (= not difficult at all) ---------------------------- 7 (= very difficult) |
| 1. Not having a car | 1 (= not difficult at all) ---------------------------- 7 (= very difficult) |
| 1. Being in a bad mood | 1 (= not difficult at all) ---------------------------- 7 (= very difficult) |
| 1. High stress levels | 1 (= not difficult at all) ---------------------------- 7 (= very difficult) |
| 1. Lack of necessary equipment | 1 (= not difficult at all) ---------------------------- 7 (= very difficult) |
| 1. When there are no people around me who are physically active | 1 (= not difficult at all) ---------------------------- 7 (= very difficult) |
| 1. My children | 1 (= not difficult at all) ---------------------------- 7 (= very difficult) |
| 1. The Covid-19 situation | 1 (= not difficult at all) ---------------------------- 7 (= very difficult) |
| 1. My health problems | 1 (= not difficult at all) ---------------------------- 7 (= very difficult) |
| 1. Not seeing the results fast enough | 1 (= not difficult at all) ---------------------------- 7 (= very difficult) |
| 1. Other priorities that I have | 1 (= not difficult at all) ---------------------------- 7 (= very difficult) |

**Intentions**

15. Please answer the following

| 1. I would like to be more physically active | 1 (= not true at all) ---------------------------- 7 (= very true) |
| --- | --- |
| 1. I have already made plans on how to be more physically active in the future. | 1 (= not true at all) ---------------------------- 7 (= very true) |

**Questionnaire for third phase in Estonian**

Tere, tänan sind, et leidsid aega selle küsimustiku täitmiseks. Alguses on mõned küsimused sinu tausta kohta ja pärast seda esitame sulle küsimusi, mis on seotud kehalise aktiivsusega. Küsimustiku põhieesmärk on välja selgitada, millised on kehalise aktiivsuse tagajärjed sinu arvates, kuidas teised inimesed sinu kehalise aktiivsuse taset näevad ja millised tegurid sinu kehalise aktiivsuse taset mõjutavad. Kokku on 15 küsimust. Küsimustiku täitmine ei kesta kauem kui 15 minutit. Täida küsimustik ainult siis, kui oled täiskoormusega ülikooli üliõpilane.

**Taustainfo**

1. Sugu. Kui valid "muu”, täpsustage palun alla "Palun lisa oma kommentaar siia:”

- Mees
- Naine
- Muu:_______________________________________________________________

1. Palun sisestage allpool oma vanus.
2. Mis on ülikooli nimi, kus sa õpid?
3. Mida sa õpid?
4. Kas sa töötad? Kui jah, kirjutage palun alla "Palun lisa oma kommentaar siia:" millist tööd teed.

- Jah:_______________________________________________________________
- Ei

1. Kui sageli oled sa viimase kolme (3) kuu jooksul tegelenud vabal ajal aktiivse liikumisega (nt tervisesport, aiatöö, kiires tempos jalgrattasõit või kõndimine) vähemalt poole tunni vältel korraga, nii et hakkad kergelt hingeldama või higistama?

- Mitte kordagi
- Umbes kord kuus või harvem
- 2-3 korda kuus
- Kord nädalas
- 2-3 korda nädalas
- 4-6 korda nädalas
- Iga päev

**Füüsilise aktiivsuse tagajärjed**

1. Järgnevalt on loetletud mõned võimalikud kehalise aktiivsusega (nt sport, aiatöö, kiires tempos jalgrattasõit või kõndimine) seostatavad füüsilised ja vaimsed tagajärjed. Palun anna igal real hinnang, kui tõenäoliseks sa neid enda kohta pead. Need tagajärjed võivad ilmneda füüsilise tegevuse ajal, aga ka pärast füüsilist tegevust.

| 1. Vigastus(ed) | 1 (= pole üldse tõenäoline) --------------------------- 7 (= väga tõenäoline) |
| --- | --- |
| 1. Muud tegevuste jaoks vähem aega | 1 (= pole üldse tõenäoline) --------------------------- 7 (= väga tõenäoline) |
| 1. Väsimustunne | 1 (= pole üldse tõenäoline) --------------------------- 7 (= väga tõenäoline) |
| 1. Tunne, et sa oled midagi saavutanud | 1 (= pole üldse tõenäoline) --------------------------- 7 (= väga tõenäoline) |
| 1. Parem füüsiline tervis | 1 (= pole üldse tõenäoline) --------------------------- 7 (= väga tõenäoline) |
| 1. Parem vaimne tervis | 1 (= pole üldse tõenäoline) --------------------------- 7 (= väga tõenäoline) |
| 1. Parem välimus | 1 (= pole üldse tõenäoline) --------------------------- 7 (= väga tõenäoline) |
| 1. Parem uni | 1 (= pole üldse tõenäoline) --------------------------- 7 (= väga tõenäoline) |
| 1. Hea enesetunne | 1 (= pole üldse tõenäoline) --------------------------- 7 (= väga tõenäoline) |
| 1. Higistamine | 1 (= pole üldse tõenäoline) --------------------------- 7 (= väga tõenäoline) |
| 1. Ebamugavustunne | 1 (= pole üldse tõenäoline) --------------------------- 7 (= väga tõenäoline) |
| 1. Valu tundmine (mitte normaalne lihaste pingutamist “valulik“ tunne) | 1 (= pole üldse tõenäoline) --------------------------- 7 (= väga tõenäoline) |
| 1. Kurnatuse tunne | 1 (= pole üldse tõenäoline) --------------------------- 7 (= väga tõenäoline) |
| 1. Rohkem energiat | 1 (= pole üldse tõenäoline) --------------------------- 7 (= väga tõenäoline) |
| 1. Parem tuju | 1 (= pole üldse tõenäoline) --------------------------- 7 (= väga tõenäoline) |
| 1. Parem üldine tervis | 1 (= pole üldse tõenäoline) --------------------------- 7 (= väga tõenäoline) |
| 1. Stressi maandamine | 1 (= pole üldse tõenäoline) --------------------------- 7 (= väga tõenäoline) |
| 1. Tervise säilitamine | 1 (= pole üldse tõenäoline) --------------------------- 7 (= väga tõenäoline) |
| 1. Parem keskendusvōime | 1 (= pole üldse tõenäoline) --------------------------- 7 (= väga tõenäoline) |
| 1. Vähem tervisemuresid | 1 (= pole üldse tõenäoline) --------------------------- 7 (= väga tõenäoline) |
| 1. Füüsilise jōu tōus | 1 (= pole üldse tõenäoline) --------------------------- 7 (= väga tõenäoline) |
| 1. Terviseprobleemide ennetamine | 1 (= pole üldse tõenäoline) --------------------------- 7 (= väga tõenäoline) |
| 1. Parem enesehinnang | 1 (= pole üldse tõenäoline) --------------------------- 7 (= väga tõenäoline) |
| 1. Lihasvalu | 1 (= pole üldse tõenäoline) --------------------------- 7 (= väga tõenäoline) |
| 1. Enesega rahulolu | 1 (= pole üldse tõenäoline) --------------------------- 7 (= väga tõenäoline) |
| 1. Koolist/tööst puhkamine | 1 (= pole üldse tõenäoline) --------------------------- 7 (= väga tõenäoline) |
| 1. Vormis püsimine | 1 (= pole üldse tõenäoline) --------------------------- 7 (= väga tõenäoline) |
| 1. Kaalutōusu vältimine | 1 (= pole üldse tõenäoline) --------------------------- 7 (= väga tõenäoline) |
| 1. Kaalu suurenemine (nt lihasmassi) | 1 (= pole üldse tõenäoline) --------------------------- 7 (= väga tõenäoline) |
| 1. Stabiilne kaal | 1 (= pole üldse tõenäoline) --------------------------- 7 (= väga tõenäoline) |
| 1. Meelerahu | 1 (= pole üldse tõenäoline) --------------------------- 7 (= väga tõenäoline) |
| 1. Parem enesetunne | 1 (= pole üldse tõenäoline) --------------------------- 7 (= väga tõenäoline) |
| 1. Parem rüht | 1 (= pole üldse tõenäoline) --------------------------- 7 (= väga tõenäoline) |
| 1. Pikem eluiga | 1 (= pole üldse tõenäoline) --------------------------- 7 (= väga tõenäoline) |
| 1. Suurem töövõime | 1 (= pole üldse tõenäoline) --------------------------- 7 (= väga tõenäoline) |

1. Mil määral oled valmis leppima järgmiste kehalise aktiivsuse tagajärgedega (-3 ei ole üldse valmis leppima; 3 olen täiesti valmis leppima)?

*Siin tähendab "valmis leppima", et see on sinu jaoks aktsepteeritav ja võid lasta sel juhtuda. Samas "ei valmis leppima" tähendab, et see pole sinu jaoks aktsepteeritav ja sulle ei sobi, et selline asi juhtub.*

| 1. Vigastus(ed) | -3 (= ei ole üldse valmis leppima)--3 (= olen täiesti valmis leppima) |
| --- | --- |
| 1. Vähem aega muude asjade jaoks (näiteks töö, kool, teised kohustused) | -3 (= ei ole üldse valmis leppima)--3 (= olen täiesti valmis leppima) |
| 1. Väsimustunne | -3 (= ei ole üldse valmis leppima)--3 (= olen täiesti valmis leppima) |
| 1. Higistamine | -3 (= ei ole üldse valmis leppima)--3 (= olen täiesti valmis leppima) |
| 1. Ebamugavustunne | -3 (= ei ole üldse valmis leppima)--3 (= olen täiesti valmis leppima) |
| 1. Valu tundmine (mitte normaalne lihaste pingutamist “valulik“ tunne) | -3 (= ei ole üldse valmis leppima)--3 (= olen täiesti valmis leppima) |
| 1. Kurnatuse tunne | -3 (= ei ole üldse valmis leppima)--3 (= olen täiesti valmis leppima) |
| 1. Lihasvalu | -3 (= ei ole üldse valmis leppima)--3 (= olen täiesti valmis leppima) |

**Füüsiline aktiivsus ja teised inimesed**

9. Järgnevad inimesed arvavad, et peaksin olema kehaliselt aktiivsem ...

*Kui ühte või mitut neist inimestest pole olemas või sa ei ole nendega kontaktis, siis valige "Vastust pole”. Samuti, kui sa ei tea, mida teine inimene arvab, valige "Vastust pole".*

| 1. Minu ema | 1 (= pole üldse ōige) ------------------------- 7 (= väga ōige) |
| --- | --- |
| 1. Minu isa | 1 (= pole üldse ōige) ------------------------- 7 (= väga ōige) |
| 1. Minu vend/vennad | 1 (= pole üldse ōige) ------------------------- 7 (= väga ōige) |
| 1. Minu ōde/ōed | 1 (= pole üldse ōige) ------------------------- 7 (= väga ōige) |
| 1. Minu sõber/sōbrad | 1 (= pole üldse ōige) ------------------------- 7 (= väga ōige) |
| 1. Minu kolleeg(id) | 1 (= pole üldse ōige) ------------------------- 7 (= väga ōige) |
| 1. Teised (üli)ōpilased | 1 (= pole üldse ōige) ------------------------- 7 (= väga ōige) |
| 1. Minu elukaaslane/partner | 1 (= pole üldse ōige) ------------------------- 7 (= väga ōige) |
| 1. Minu laps(ed) | 1 (= pole üldse ōige) ------------------------- 7 (= väga ōige) |
| 1. Sugulased (v.a vanemad, ōed-vennad ja lapsed) | 1 (= pole üldse ōige) ------------------------- 7 (= väga ōige) |

10. Järgmised inimesed on regulaarselt kehaliselt aktiivsed ...

*Kui ühte või mitut neist inimestest ei eksisteeri, valige "Vastust pole". Samuti, kui sa ei tea, kas teine inimene on regulaarselt füüsiliselt aktiivne, valige "Vastust pole".*

| 1. Minu ema | 1 (= pole üldse ōige) ------------------------- 7 (= väga ōige) |
| --- | --- |
| 1. Minu isa | 1 (= pole üldse ōige) ------------------------- 7 (= väga ōige) |
| 1. Minu vend/vennad | 1 (= pole üldse ōige) ------------------------- 7 (= väga ōige) |
| 1. Minu ōde/ōed | 1 (= pole üldse ōige) ------------------------- 7 (= väga ōige) |
| 1. Minu sõber/sōbrad | 1 (= pole üldse ōige) ------------------------- 7 (= väga ōige) |
| 1. Minu kolleeg(id) | 1 (= pole üldse ōige) ------------------------- 7 (= väga ōige) |
| 1. Teised (üli)ōpilased | 1 (= pole üldse ōige) ------------------------- 7 (= väga ōige) |
| 1. Minu elukaaslane/partner | 1 (= pole üldse ōige) ------------------------- 7 (= väga ōige) |
| 1. Minu laps(ed) | 1 (= pole üldse ōige) ------------------------- 7 (= väga ōige) |
| 1. Sugulased (v.a vanemad, ōed-vennad ja lapsed) | 1 (= pole üldse ōige) ------------------------- 7 (= väga ōige) |

1. Millisel määral sulle läheb korda järgmiste inimeste arvamus sinu kehalise aktiivsuse kohta? (nt kui sinu ema ütleb sulle, et sa peaksid olema kehaliselt aktiivsem, siis kui palju tema arvamus sulle korda läheb?)

*Kui ühte või mitut neist inimestest ei eksisteeri, valige “Vastust pole“. Samuti, kui sa ei tea, mida teine inimene arvab, valige palun "Vastust pole”.*

| 1. Minu ema | 1 (= ei hooli üldse) ------------------------- 7 (= ma hoolin väga) |
| --- | --- |
| 1. Minu isa | 1 (= ei hooli üldse) ------------------------- 7 (= ma hoolin väga) |
| 1. Minu vend/vennad | 1 (= ei hooli üldse) ------------------------- 7 (= ma hoolin väga) |
| 1. Minu ōde/ōed | 1 (= ei hooli üldse) ------------------------- 7 (= ma hoolin väga) |
| 1. Minu sõber/sōbrad | 1 (= ei hooli üldse) ------------------------- 7 (= ma hoolin väga) |
| 1. Minu kolleeg(id) | 1 (= ei hooli üldse) ------------------------- 7 (= ma hoolin väga) |
| 1. Teised (üli)ōpilased | 1 (= ei hooli üldse) ------------------------- 7 (= ma hoolin väga) |
| 1. Minu elukaaslane/partner | 1 (= ei hooli üldse) ------------------------- 7 (= ma hoolin väga) |
| 1. Minu laps(ed) | 1 (= ei hooli üldse) ------------------------- 7 (= ma hoolin väga) |
| 1. Sugulased (v.a vanemad, ōed-vennad ja lapsed) | 1 (= ei hooli üldse) ------------------------- 7 (= ma hoolin väga) |

**Erinevad tegurid, mis mõjutavad füüsilist aktiivsust**

1. Kehaliselt aktiivseks olemiseks kuluv aeg sõltub sellest...

*Kui üks või mitu neist valikutest ei kehti sinu kohta, valige "vastust pole".*

| 1. Ilmast | 1 (= pole üldse ōige) -------------------7 (= väga ōige) |
| --- | --- |
| 1. Kas mul on aega või mitte | 1 (= pole üldse ōige) -------------------7 (= väga ōige) |
| 1. Kui motiveeritud ma olen | 1 (= pole üldse ōige) -------------------7 (= väga ōige) |
| 1. Minu majanduslikust olukorrast | 1 (= pole üldse ōige) -------------------7 (= väga ōige) |
| 1. Kuidas ma end tunnen | 1 (= pole üldse ōige) -------------------7 (= väga ōige) |
| 1. Minu energiatasemest | 1 (= pole üldse ōige) -------------------7 (= väga ōige) |
| 1. Kus ma elan | 1 (= pole üldse ōige) -------------------7 (= väga ōige) |
| 1. Kas läheduses on jōusaal | 1 (= pole üldse ōige) -------------------7 (= väga ōige) |
| 1. Kas jõusaal on avatud või mitte | 1 (= pole üldse ōige) -------------------7 (= väga ōige) |
| 1. Kas mul on kellegagi seda teha või mitte (nt kellegagi koos sportida, treenida jne.) | 1 (= pole üldse ōige) -------------------7 (= väga ōige) |
| 1. Minu planeerimisoskusest | 1 (= pole üldse ōige) -------------------7 (= väga ōige) |
| 1. Kas keskkond on ohutu | 1 (= pole üldse ōige) -------------------7 (= väga ōige) |
| 1. Kohustuste mahust, mis mul on | 1 (= pole üldse ōige) -------------------7 (= väga ōige) |
| 1. Kui palju tööga või kooliga seotud kohustusis mul on | 1 (= pole üldse ōige) -------------------7 (= väga ōige) |
| 1. Kas mul on auto või mitte (nt jõusaali minna) | 1 (= pole üldse ōige) -------------------7 (= väga ōige) |
| 1. Minu tujust | 1 (= pole üldse ōige) -------------------7 (= väga ōige) |
| 1. Minu stressitasemest | 1 (= pole üldse ōige) -------------------7 (= väga ōige) |
| 1. Vajalike seadmete olemasolust | 1 (= pole üldse ōige) -------------------7 (= väga ōige) |
| 1. Kas inimesed minu ümber on füüsiliselt aktiivsed | 1 (= pole üldse ōige) -------------------7 (= väga ōige) |
| 1. Mu lastest | 1 (= pole üldse ōige) -------------------7 (= väga ōige) |
| 1. Covid-19 olukorrast | 1 (= pole üldse ōige) -------------------7 (= väga ōige) |
| 1. Minu tervisest | 1 (= pole üldse ōige) -------------------7 (= väga ōige) |
| 1. Kui kiiresti ma tulemusi näen | 1 (= pole üldse ōige) -------------------7 (= väga ōige) |
| 1. Minu teistest eesmärkidest | 1 (= pole üldse ōige) -------------------7 (= väga ōige) |

13. Palun vastake järgmisetele väidetele:

| 1. Aeg, mille kulutan kehaliselt aktiivsele tegevusele, sõltub täielikult minust. | 1 (= pole üldse ōige) -------------------7 (= väga ōige) |
| --- | --- |
| 1. Olen kindel, et ma saan oma kehalist aktiivsust suurendada. | 1 (= pole üldse ōige) -------------------7 (= väga ōige) |

**Erinevate tegurite mõju füüsilise aktiivsuse tasemele**

14. Mil määral teevad järgmised tegurid kehalise aktiivsuse sinu jaoks raskeks?

*Kui üks või mitu neist valikutest ei kehti sinu kohta, valige "vastust pole".*

| 1. Halb ilm | 1 (= üldse mitte) -------------------7 (= väga suurel maaral) |
| --- | --- |
| 1. Pole aega | 1 (= üldse mitte) -------------------7 (= väga suurel maaral) |
| 1. Madal motivatsioon | 1 (= üldse mitte) -------------------7 (= väga suurel maaral) |
| 1. Minu majanduslik olukord | 1 (= üldse mitte) -------------------7 (= väga suurel maaral) |
| 1. Halb enesetunne | 1 (= üldse mitte) -------------------7 (= väga suurel maaral) |
| 1. Madal energiatase | 1 (= üldse mitte) -------------------7 (= väga suurel maaral) |
| 1. Koht, kus ma elan | 1 (= üldse mitte) -------------------7 (= väga suurel maaral) |
| 1. Läheduses pole jōusaali | 1 (= üldse mitte) -------------------7 (= väga suurel maaral) |
| 1. Jõusaal (vōi mōni muu spordirajatis) on suletud | 1 (= üldse mitte) -------------------7 (= väga suurel maaral) |
| 1. Kui mul pole kellegagi seda teha (nt kellegagi koos sportida, treenida jne.) | 1 (= üldse mitte) -------------------7 (= väga suurel maaral) |
| 1. Minu planeerimisoskus | 1 (= üldse mitte) -------------------7 (= väga suurel maaral) |
| 1. Elamine ebaturvalises keskkonnas | 1 (= üldse mitte) -------------------7 (= väga suurel maaral) |
| 1. Paljude kohustuste omamine | 1 (= üldse mitte) -------------------7 (= väga suurel maaral) |
| 1. Palju tööga vōi kooliga seotud kohustusis | 1 (= üldse mitte) -------------------7 (= väga suurel maaral) |
| 1. Auto puudumine | 1 (= üldse mitte) -------------------7 (= väga suurel maaral) |
| 1. Halvas tujus olemine | 1 (= üldse mitte) -------------------7 (= väga suurel maaral) |
| 1. Kõrge stressitase | 1 (= üldse mitte) -------------------7 (= väga suurel maaral) |
| 1. Vajalike seadmete puudumine | 1 (= üldse mitte) -------------------7 (= väga suurel maaral) |
| 1. Kui mu ümber pole inimesi, kes on füüsiliselt aktiivsed | 1 (= üldse mitte) -------------------7 (= väga suurel maaral) |
| 1. Minu lapsed | 1 (= üldse mitte) -------------------7 (= väga suurel maaral) |
| 1. Covid-19 olukord | 1 (= üldse mitte) -------------------7 (= väga suurel maaral) |
| 1. Minu terviseprobleemid | 1 (= üldse mitte) -------------------7 (= väga suurel maaral) |
| 1. Ei näe tulemusi piisavalt kiiresti | 1 (= üldse mitte) -------------------7 (= väga suurel maaral) |
| 1. Muud prioriteedid, mis mul on | 1 (= üldse mitte) -------------------7 (= väga suurel maaral) |

**Kavatsused**

15. Palun vastake järgmisetele väidetele:

| 1. Tahaksin olla kehaliselt aktiivsem. | 1 (= pole üldse tõsi)------------------------------------7 (= väga tõsi) |
| --- | --- |
| 1. Olen juba teinud plaane selle kohta, kuidas tulevikus olla kehaliselt aktiivsem. | 1 (= pole üldse tõsi)------------------------------------7 (= väga tõsi) |
