# Supplementary material for: Toward Comprehensive Assessment of Beliefs and Attitudes Related to Physical Activity in Young Adults: Pilot Study
Source: JMIR Form Res. 2025 Oct 16;9:e69094. doi: 10.2196/69094 (PMC12576300; doi:10.2196/69094)
Supplement: Multimedia Appendix 4 [file formative_v9i1e69094_app4.docx]

**Theory of Planned Behaviour Questionnaire in English**

1. Listed below are some of the possible physical and mental consequences associated with physical activity (e.g., sports, gardening, cycling or walking at a fast pace). Please rate on each line how likely you think the consequence is for you. These effects can occur during physical activity, as well as after physical activity.

| 1. Feeling better | (not at all likely) 1---------------------7 (very likely) |
| --- | --- |
| 1. Better physical health | (not at all likely) 1---------------------7 (very likely) |
| 1. Better long-term health (e.g., during the lifetime) | (not at all likely) 1---------------------7 (very likely) |
| 1. Healthy weight | (not at all likely) 1---------------------7 (very likely) |
| 1. Better physical shape | (not at all likely) 1---------------------7 (very likely) |
| 1. Being satisfied with myself | (not at all likely) 1---------------------7 (very likely) |
| 1. Feeling pain (not a normal “painful” feeling of muscle tension/pain) | (not at all likely) 1---------------------7 (very likely) |
| 1. Muscle pain | (not at all likely) 1---------------------7 (very likely) |
| 1. Injury/injuries | (not at all likely) 1---------------------7 (very likely) |
| 1. Feeling tired | (not at all likely) 1---------------------7 (very likely) |
| 1. Feeling exhausted | (not at all likely) 1---------------------7 (very likely) |
| 1. Sweating | (not at all likely) 1---------------------7 (very likely) |
| 1. Feeling uncomfortable | (not at all likely) 1---------------------7 (very likely) |
| 1. Less time for other activities (e.g., work, school, other obligations) | (not at all likely) 1---------------------7 (very likely) |

2. To what extent are you willing to accept the following consequences of physical activity (0 = not at all willing to accept; 7 = I am completely willing to accept)?

*Here, "willing to accept" means that it is acceptable to you and it is ok if it happens. At the same time, "not willing to accept" means that it is not acceptable for you and it does not suit you that such a thing would happen.*

| 1. Feeling pain | (not at all willing to accept) 1------------7 (I am completely willing to accept) |
| --- | --- |
| 1. Muscle pain | (not at all willing to accept) 1------------7 (I am completely willing to accept) |
| 1. Injury/injuries | (not at all willing to accept) 1------------7 (I am completely willing to accept) |
| 1. Feeling tired | (not at all willing to accept) 1------------7 (I am completely willing to accept) |
| 1. Feeling exhausted | (not at all willing to accept) 1------------7 (I am completely willing to accept) |
| 1. Sweating | (not at all willing to accept) 1------------7 (I am completely willing to accept) |
| 1. Feeling uncomfortable | (not at all willing to accept) 1------------7 (I am completely willing to accept) |
| 1. Less time for other activities (e.g., work, school, other obligations) | (not at all willing to accept) 1------------7 (I am completely willing to accept) |

3. Rate to what extent you think the following people think you should be more physically active.

*If one or more of these people do not exist or you are not in contact with them, select "No answer". Also, if you do not know what the other person thinks, select "No answer".*

| 1. My parent(s) | (not true at all) 1----------------------7 (very true) |
| --- | --- |
| 1. My brother(s) and/or sister(s) | (not true at all) 1----------------------7 (very true) |
| 1. My friends | (not true at all) 1----------------------7 (very true) |
| 1. My work and/or study partners (e.g., colleagues, fellow students) | (not true at all) 1----------------------7 (very true) |
| 1. My partner | (not true at all) 1----------------------7 (very true) |
| 1. My children | (not true at all) 1----------------------7 (very true) |

4. Estimate the extent to which the mentioned people are regularly physically active themselves.

*If one or more of these people do not exist, select "No answer". Also, if you don't know if the other person is regularly physically active, choose "No answer".*

| 1. My parent(s) | (not true at all) 1----------------------7 (very true) |
| --- | --- |
| 1. My brother(s) and/or sister(s) | (not true at all) 1----------------------7 (very true) |
| 1. My friends | (not true at all) 1----------------------7 (very true) |
| 1. My work and/or study partners (e.g., colleagues, fellow students) | (not true at all) 1----------------------7 (very true) |
| 1. My partner | (not true at all) 1----------------------7 (very true) |
| 1. My children | (not true at all) 1----------------------7 (very true) |

5. To what extent do you care about the opinions of the following people about your physical activity levels? (e.g., if your mother tells you that you should be more physically active, how much does her opinion matter to you?)

*If one or more of these people do not exist, select "No answer". Also, if you don't know what the other person thinks, select "No answer".*

| 1. My parent(s) | (don’t care at all) 1----------------------7 (I care a lot) |
| --- | --- |
| 1. My brother(s) and/or sister(s) | (don’t care at all) 1----------------------7 (I care a lot) |
| 1. My friends | (don’t care at all) 1----------------------7 (I care a lot) |
| 1. My work and/or study partners (e.g., colleagues, fellow students) | (don’t care at all) 1----------------------7 (I care a lot) |
| 1. My partner | (don’t care at all) 1----------------------7 (I care a lot) |
| 1. My children | (don’t care at all) 1----------------------7 (I care a lot) |

6. Evaluate what your physical activity levels depend on. My time to be physically active depends (on)...

| 1. The weather | (not true at all) 1----------------------7 (very true) |
| --- | --- |
| 1. How I am feeling | (not true at all) 1----------------------7 (very true) |
| 1. My health | (not true at all) 1----------------------7 (very true) |
| 1. My planning skills | (not true at all) 1----------------------7 (very true) |
| 1. How fast I see the results | (not true at all) 1----------------------7 (very true) |
| 1. Do I have time or not | (not true at all) 1----------------------7 (very true) |
| 1. The distance to the place to be physically active (e.g., gym, swimming pool etc.) | (not true at all) 1----------------------7 (very true) |
| 1. The possibilities to use to place to be physically active (e.g., tennis court is closed) | (not true at all) 1----------------------7 (very true) |
| 1. Transportation possibilities (e.g., go with the bus to the swimming pool) | (not true at all) 1----------------------7 (very true) |
| 1. Availability of the necessary equipment (e.g., yoga mat, dumbbells) | (not true at all) 1----------------------7 (very true) |
| 1. My financial situation | (not true at all) 1----------------------7 (very true) |
| 1. The place where I live | (not true at all) 1----------------------7 (very true) |
| 1. The safety of the place where I live | (not true at all) 1----------------------7 (very true) |
| 1. My social environment (e.g., there are no people around me who are physically active, when I do not have someone to do it with). | (not true at all) 1----------------------7 (very true) |
| 1. The Covid-19 situation | (not true at all) 1----------------------7 (very true) |

7. Please answer the following questions:

| 1. The amount of time I spend on physical activity depends mainly on factors (such as the weather) that are outside of my control. | (not true at all) 1----------------------7 (very true) |
| --- | --- |
| 1. I am confident that I can increase my physical activity. | (not true at all) 1----------------------7 (very true) |
| 1. I would like to be more physically active. | (not true at all) 1----------------------7 (very true) |
| 1. I have already made plans how to be more physically active in the future. | (not true at all) 1----------------------7 (very true) |

**Theory of Planned Behaviour Questionnaire in Estonian**

1. Järgnevalt on loetletud mõned võimalikud kehalise aktiivsusega (nt sport, aiatöö, kiires tempos jalgrattasõit või kõndimine) seostatavad füüsilised ja vaimsed tagajärjed. Palun anna igal real hinnang, kui tõenäoliseks sa neid enda kohta pead. Need tagajärjed võivad ilmneda kehalise tegevuse ajal, aga ka pärast kehalist tegevust.

| 1. Parem enesetunne | (pole üldse tōenäoline) 1----------------------7 (väga tōenäoline) |
| --- | --- |
| 1. Parem füüsiline tervis | (pole üldse tōenäoline) 1----------------------7 (väga tōenäoline) |
| 1. Parem pikaajaline tervis (nt. elu jooksul) | (pole üldse tōenäoline) 1----------------------7 (väga tōenäoline) |
| 1. Tervislik kaal | (pole üldse tōenäoline) 1----------------------7 (väga tōenäoline) |
| 1. Parem füüsiline vorm | (pole üldse tōenäoline) 1----------------------7 (väga tōenäoline) |
| 1. Enesega rahulolu | (pole üldse tōenäoline) 1----------------------7 (väga tōenäoline) |
| 1. Valutunne (mitte normaalne lihaste pingutamisest "valulik" tunne) | (pole üldse tōenäoline) 1----------------------7 (väga tōenäoline) |
| 1. Lihasvalu | (pole üldse tōenäoline) 1----------------------7 (väga tōenäoline) |
| 1. Vigastus(ed) | (pole üldse tōenäoline) 1----------------------7 (väga tōenäoline) |
| 1. Väsimustunne | (pole üldse tōenäoline) 1----------------------7 (väga tōenäoline) |
| 1. Kurnatuse tunne | (pole üldse tōenäoline) 1----------------------7 (väga tōenäoline) |
| 1. Higistamine | (pole üldse tōenäoline) 1----------------------7 (väga tōenäoline) |
| 1. Ebamugavustunne | (pole üldse tōenäoline) 1----------------------7 (väga tōenäoline) |
| 1. Vähem aega muude asjade jaoks (näiteks töö, kool, teised kohustused) | (pole üldse tōenäoline) 1----------------------7 (väga tōenäoline) |

2. Mil määral oled valmis leppima järgmiste kehalise aktiivsuse tagajärgedega (0 ei ole üldse valmis leppima; 7 olen täiesti valmis leppima)?

*Siin tähendab "valmis leppima", et see on sinu jaoks aktsepteeritav ja võid lasta sel juhtuda. Samas "ei valmis leppima" tähendab, et see pole sinu jaoks aktsepteeritav ja sulle ei sobi, et selline asi juhtub.*

| 1. Valutunne | (ei ole üldse valmis leppima) 1----------------------7 (olen täiesti valmis leppima) |
| --- | --- |
| 1. Lihasvalu | (ei ole üldse valmis leppima) 1----------------------7 (olen täiesti valmis leppima) |
| 1. Vigastus(ed) | (ei ole üldse valmis leppima) 1----------------------7 (olen täiesti valmis leppima) |
| 1. Väsimustunne | (ei ole üldse valmis leppima) 1----------------------7 (olen täiesti valmis leppima) |
| 1. Kurnatuse tunne | (ei ole üldse valmis leppima) 1----------------------7 (olen täiesti valmis leppima) |
| 1. Higistamine | (ei ole üldse valmis leppima) 1----------------------7 (olen täiesti valmis leppima) |
| 1. Ebamugavustunne | (ei ole üldse valmis leppima) 1----------------------7 (olen täiesti valmis leppima) |
| 1. Vähem aega muude asjade jaoks (näiteks töö, kool, teised kohustused) | (ei ole üldse valmis leppima) 1----------------------7 (olen täiesti valmis leppima) |

3. Hinda, mil määral sinu meelest järgnevalt nimetatud inimesed arvavad, et sina peaksid olema kehaliselt aktiivsem.

*Kui üht või mitut neist inimestest pole olemas või sa ei ole nendega kontaktis, siis vali "Vastust pole”. Samuti, kui sa ei tea, mida teine inimene arvab, vali "Vastust pole".*

| 1. Minu vanem(ad) | (pole üldse tōsi) 1----------------------7 (väga tōsi) |
| --- | --- |
| 1. Minu vend/vennad ja/vōi ōde/ōed | (pole üldse tōsi) 1----------------------7 (väga tōsi) |
| 1. Minu sōbrad | (pole üldse tōsi) 1----------------------7 (väga tōsi) |
| 1. Minu töö- ja/vōi ōpingukaaslased (nt kolleegid, kaasüliōpilased) | (pole üldse tōsi) 1----------------------7 (väga tōsi) |
| 1. Minu elukaaslane/partner/abikaasa | (pole üldse tōsi) 1----------------------7 (väga tōsi) |
| 1. Minu lapsed | (pole üldse tōsi) 1----------------------7 (väga tōsi) |

4. Hinda, mil määral järnevalt nimetatud inimesed on ise regulaarselt kehaliselt aktiivsed...

*Kui üht või mitut neist inimestest ei eksisteeri, vali "Vastust pole". Samuti, kui sa ei tea, kas teine inimene on regulaarselt kehaliselt aktiivne, vali "Vastust pole".*

| 1. Minu vanem(ad) | (pole üldse ōige) 1----------------------7 (väga ōige) |
| --- | --- |
| 1. Minu vend/vennad ja/vōi ōde/ōed | (pole üldse ōige) 1----------------------7 (väga ōige) |
| 1. Minu sōbrad | (pole üldse ōige) 1----------------------7 (väga ōige) |
| 1. Minu töö- ja/vōi ōpingukaaslased (nt kolleegid, kaasüliōpilased) | (pole üldse ōige) 1----------------------7 (väga ōige) |
| 1. Minu elukaaslane/partner/abikaasa | (pole üldse ōige) 1----------------------7 (väga ōige) |
| 1. Minu lapsed | (pole üldse ōige) 1----------------------7 (väga ōige) |

5. Mil määral läheb sulle korda järgmiste inimeste arvamus sinu kehalise aktiivsuse kohta? (nt kui sinu ema ütleb sulle, et sa peaksid olema kehaliselt aktiivsem, siis kui palju tema arvamus sulle korda läheb?)

*Kui üht või mitut neist inimestest ei eksisteeri, vali “Vastust pole“. Samuti, kui sa ei tea, mida teine inimene arvab, vali "Vastust pole”.*

| 1. Minu vanem(ad) | (ei hooli üldse) 1----------------------7 (ma hoolin väga) |
| --- | --- |
| 1. Minu vend/vennad ja/vōi ōde/ōed | (ei hooli üldse) 1----------------------7 (ma hoolin väga) |
| 1. Minu sōbrad | (ei hooli üldse) 1----------------------7 (ma hoolin väga) |
| 1. Minu töö- ja/vōi ōpingukaaslased (nt kolleegid, kaasüliōpilased) | (ei hooli üldse) 1----------------------7 (ma hoolin väga) |
| 1. Minu elukaaslane/partner/abikaasa | (ei hooli üldse) 1----------------------7 (ma hoolin väga) |
| 1. Minu lapsed | (ei hooli üldse) 1----------------------7 (ma hoolin väga) |

6. Hinda, millest sōltub sinu kehalisele aktiivsusele kuluv aeg. Minu kehaliselt aktiivne olemiseks aeg sōltub (sellest)...

| 1. Ilmast | (pole üldse tōsi) 1----------------------7 (väga tōsi) |
| --- | --- |
| 1. Minu enesetundest | (pole üldse tōsi) 1----------------------7 (väga tōsi) |
| 1. Minu tervisest | (pole üldse tōsi) 1----------------------7 (väga tōsi) |
| 1. Minu planeerimisoskusest | (pole üldse tōsi) 1----------------------7 (väga tōsi) |
| 1. Kui kiiresti ma tulemusi näen | (pole üldse tōsi) 1----------------------7 (väga tōsi) |
| 1. Kas mul on aega vōi mitte | (pole üldse tōsi) 1----------------------7 (väga tōsi) |
| 1. Kaugusest kehalise aktiivsuse kohani (nt jōusaal, bassein, jne.) | (pole üldse tōsi) 1----------------------7 (väga tōsi) |
| 1. Kehalise aktiivsuse koha kasutamise vōimalutest (nt tenniseväljak on suletud) | (pole üldse tōsi) 1----------------------7 (väga tōsi) |
| 1. Transportdivōimalustest kehalise aktiivsuse jaoks (nt bussiga ujulasse minekuks) | (pole üldse tōsi) 1----------------------7 (väga tōsi) |
| 1. Vajalike seadmete olemasolust (nt joogamatt, hantlid) | (pole üldse tōsi) 1----------------------7 (väga tōsi) |
| 1. Minu majanduslikust olukorrast | (pole üldse tōsi) 1----------------------7 (väga tōsi) |
| 1. Kohast, kus ma elan | (pole üldse tōsi) 1----------------------7 (väga tōsi) |
| 1. Elukoha turvalisusest | (pole üldse tōsi) 1----------------------7 (väga tōsi) |
| 1. Minu sotsiaalsest keskkonnast (nt mu pole ümber pole inimesi, kes on kehaliselt aktiivsed, kui mul pole kellegagi seda teha) | (pole üldse tōsi) 1----------------------7 (väga tōsi) |
| 1. Covid-19 olukorrast | (pole üldse tōsi) 1----------------------7 (väga tōsi) |

7. Palun vastake järgmistele küsimustele:

| 1. Aeg, mille kulutan kehalisele aktiivsusele, sõltub peamiselt teguritest (nt ilmast), mis ei ole minu kontrolli all. | (pole üldse ōige) 1----------------------7 (väga ōige) |
| --- | --- |
| 1. Olen kindel, et ma saan oma kehalist aktiivsust suurendada. | (pole üldse ōige) 1----------------------7 (väga ōige) |
| 1. Tahaksin olla kehaliselt aktiivsem. | (pole üldse ōige) 1----------------------7 (väga ōige) |
| 1. Olen juba teinud plaane selle kohta, kuidas tulevikus olla kehaliselt aktiivsem. | (pole üldse ōige) 1----------------------7 (väga ōige) |
