# Supplementary material for: Toward Comprehensive Assessment of Beliefs and Attitudes Related to Physical Activity in Young Adults: Pilot Study
Source: JMIR Form Res. 2025 Oct 16;9:e69094. doi: 10.2196/69094 (PMC12576300; doi:10.2196/69094)
Supplement: Multimedia Appendix 5 [file formative_v9i1e69094_app5.docx]

Towards comprehensive assessment of beliefs and attitudes related to physical activity motivation in young adults: Bottom-up construction of a TPB-based questionnaire

Descriptive statistics and factor analysis results

Theodorus D.B. Noordover^1,2^, Aave Hannus^1,2^, Kenn Konstabel^2,1^

^1^Institute of Psychology, University of Tartu, Estonia

^2^National Institute for Health Development, Tallinn, Estonia

Contents

[Section 1: Descriptive and background information 2](#_Toc183079766)

[Supplementary Table 1: Behavioural beliefs associated with physical activity 3](#_Toc183079767)

[Supplementary Table 2: Control beliefs associated with physical activity 4](#_Toc183079768)

[Supplementary Table 3: Perceived power of control beliefs 5](#_Toc183079769)

[Section 2: Behavioural beliefs 5](#_Toc183079770)

[Supplementary Table 4: Behavioural beliefs: factor analysis 9](#_Toc183079771)

[Section 3: Subjective norms 9](#_Toc183079772)

[Supplementary Table 5: Injunctive norms 10](#_Toc183079773)

[Supplementary Table 6: Descriptive norms 10](#_Toc183079774)

[Supplementary Table 7: Motivation to comply 11](#_Toc183079775)

[Section 4: Control beliefs 11](#_Toc183079776)

[Supplementary Table 8: Control beliefs and their perceived power to influence behaviour: factor analysis 15](#_Toc183079777)

*Note.* When discussing factor analysis results, we will use two conventions in this supplement.

1. For interpreting the meaning of a factor, only loadings greater than or equal to 0.3 in absolute value are considered.
2. For brevity, the phrase “item X loads on factor Y” is used to mean “item X had a loading of at least 0.3 in absolute value on factor Y”. Similarly, “N items loaded on factor Y” is used to signify “N items had a loading of at least 0.3 in absolute value on factor Y”. In exploratory factor analysis, every variable is related to (or, in other words, has a loading on) every factor. Nevertheless, we are using this simplified convention to avoid long and repetitive expressions.

# Section 1: Descriptive and background information

*Background information* was gathered using five items related to gender, age, name of university, study program and occupation.

*Physical activity levels* were collected using one item (e.g., “Describe your ordinary physical activity habits...”). Physical activity was defined as "any type of movement that requires a certain amount of effort that involves a significant increase in heart rate / breathing frequency (e.g., walking, working out, cycling, jogging and tennis)” (MacIntosh et al., 2021). Information related to *attitudes* was collected with eight items, using a long-answer text (e.g., “How do you feel about the idea of being physically active?”) and two items measuring the importance of the benefits and disadvantages of physical activity (e.g., “How important are these benefits to you?”). These included 2 pairs of bipolar adjectives on a scale 7-point Likert-type scale ranging from 1 (*not important at all*) to 7 (very *important*). *Subjective norms* data was collected with two items, using a long-answer text (e.g., “Are there any people (who matter to you) who think….”).

*Perceived behavioural control* data was collected with two items, using a long-answer text (e.g., “What factors or circumstances make it….”). Data regarding *intentions* to be more physically active was collected using two items (e.g., “How much do you feel like…”). These included 2 pairs of bipolar adjectives on a scale 7-point Likert-type scale ranging from 1 (*very little*) to 7 (*a lot*). Lastly, one item was included to assess if the participants had anything else they wanted to express when thinking about being physically active (e.g., “Is there anything else that comes to mind…?”). The questionnaire was created with Google Forms.

## Supplementary Table 1: Behavioural beliefs associated with physical activity

|  | M | SD |
| --- | --- | --- |
| 1. Better physical health | 6.2 | 1.2 |
| 1. Better mental health | 6.1 | 1.3 |
| 1. Better overall health | 6.0 | 1.3 |
| 1. Maintaining health | 5.9 | 1.3 |
| 1. Feeling good | 5.9 | 1.3 |
| 1. An increase in physical strength | 5.9 | 1.3 |
| 1. Feeling better | 5.9 | 1.3 |
| 1. Staying in shape | 5.7 | 1.4 |
| 1. Feeling like you have accomplished something | 5.7 | 1.4 |
| 1. Decreasing stress | 5.7 | 1.4 |
| 1. Sweating | 5.7 | 1.4 |
| 1. Better mood | 5.7 | 1.3 |
| 1. Self-satisfaction | 5.7 | 1.3 |
| 1. Better self-esteem | 5.6 | 1.5 |
| 1. Peace of mind | 5.5 | 1.4 |
| 1. Preventing health problems | 5.4 | 1.4 |
| 1. Better sleep | 5.4 | 1.5 |
| 1. Better appearance | 5.4 | 1.6 |
| 1. Resting from school/work | 5.3 | 1.7 |
| 1. Better posture | 5.2 | 1.6 |
| 1. Longer lifespan | 5.1 | 1.6 |
| 1. Less health worries | 5.1 | 1.5 |
| 1. Stable weight | 5.0 | 1.5 |
| 1. Greater working capacity | 5.0 | 1.6 |
| 1. Better focus | 4.9 | 1.6 |
| 1. Preventing weight gain | 4.9 | 1.7 |
| 1. More energy | 4.9 | 1.4 |
| 1. Muscle pain | 4.5 | 1.6 |
| 1. Less time for other activities (e.g., work, school, other obligations) | 4.0 | 1.8 |
| 1. Feeling tired | 3.9 | 1.7 |
| 1. Weight gain (e.g., muscle mass) | 3.7 | 1.7 |
| 1. Feeling exhausted | 3.4 | 1.6 |
| 1. Feeling uncomfortable | 3.3 | 1.7 |
| 1. Injury/injuries | 3.0 | 1.5 |
| 1. Feeling pain (not a normal ”painful” feeling of muscle tension/pain) | 2.8 | 1.6 |

*Note.* This table displays the list of consequences that are believed to occur as a result of being physically active from 7 (very likely) to 1 (not likely at all). M= Mean; SD = Standard Deviation.

## Supplementary Table 2: Control beliefs associated with physical activity

|  | N | M | SD |
| --- | --- | --- | --- |
| 1. My health | 436 | 5.8 | 1.4 |
| 1. How many work or school commitments I have | 442 | 5.7 | 1.5 |
| 1. The amount of obligations I have | 437 | 5.7 | 1.5 |
| 1. How I am feeling | 441 | 5.6 | 1.5 |
| 1. Whether I have time or not | 440 | 5.5 | 1.6 |
| 1. How motivated I am | 437 | 5.5 | 1.7 |
| 1. My energy levels | 442 | 5.3 | 1.6 |
| 1. My planning skills | 436 | 4.9 | 1.9 |
| 1. My mood | 441 | 4.9 | 1.8 |
| 1. My stress levels | 438 | 4.8 | 1.9 |
| 1. My other goals | 405 | 4.6 | 1.8 |
| 1. Whether the environment is safe | 407 | 4.4 | 2.1 |
| 1. The weather | 435 | 4.1 | 2.0 |
| 1. Where I live | 432 | 3.6 | 2.1 |
| 1. How fast I see the results | 428 | 3.3 | 1.9 |
| 1. The Covid-19 situation | 404 | 3.3 | 2.2 |
| 1. Whether people around me are physically active | 432 | 3.1 | 2.0 |
| 1. My children | 69 | 3.1 | 2.2 |
| 1. Having the necessary equipment | 418 | 3.1 | 2.0 |
| 1. Whether I have someone to do it with or not (e.g., play sports, exercise with someone, etc.) | 438 | 2.9 | 2.1 |
| 1. My financial situation | 430 | 2.8 | 2.0 |
| 1. Whether the gym is open or not | 418 | 2.8 | 2.2 |
| 1. Whether there is a gym nearby | 426 | 2.7 | 2.0 |
| 1. Whether I have a car or not (e.g., to go to the gym) | 425 | 2.2 | 1.9 |

*Note.* This table displays the factors that being physically active depend on from 7 (very true) to 1 (not true at all). N = number of participants that gave an answer; M= Mean; SD = Standard Deviation. N ranges from 69 to 442 due to missing data.

## Supplementary Table 3: Perceived power of control beliefs

|  | N | M | SD |
| --- | --- | --- | --- |
| 1. A lot of work or school related obligations | 430 | 5.5 | 1.6 |
| 1. Not having time | 438 | 5.4 | 1.6 |
| 1. Having many obligations | 428 | 5.3 | 1.6 |
| 1. Feeling bad | 438 | 5.1 | 1.7 |
| 1. Low energy levels | 435 | 5.0 | 1.6 |
| 1. Low motivation | 433 | 5.0 | 1.9 |
| 1. Other priorities that I have | 418 | 4.7 | 1.9 |
| 1. High stress levels | 428 | 4.5 | 1.9 |
| 1. My planning skills | 433 | 4.5 | 1.9 |
| 1. My health problems | 404 | 4.4 | 2.0 |
| 1. Being in a bad mood | 433 | 4.2 | 1.9 |
| 1. Bad weather | 436 | 3.9 | 2.1 |
| 1. Not seeing the results fast enough | 422 | 3.1 | 1.9 |
| 1. The Covid-19 situation | 394 | 3.0 | 2.1 |
| 1. The place where I live | 427 | 2.9 | 2.0 |
| 1. Living in an unsafe environment | 365 | 2.8 | 2.0 |
| 1. My children | 73 | 2.7 | 2.1 |
| 1. When I don't have anyone to do it with (e.g., play sports, exercise with someone, etc.) | 432 | 2.7 | 1.9 |
| 1. My financial situation | 428 | 2.6 | 1.8 |
| 1. Lack of necessary equipment | 415 | 2.5 | 1.6 |
| 1. When there are no people around me who are physically active | 427 | 2.5 | 1.8 |
| 1. The gym (or other sports facility) is closed | 415 | 2.4 | 2.0 |
| 1. There is no gym nearby | 412 | 2.0 | 1.5 |
| 1. Not having a car | 409 | 1.8 | 1.4 |

*Note.* This table displays to what extent certain factors make it more difficult to be physically active from 7 (to a large extent) to 1 (not at all). N = number of participants that gave an answer; M= Mean; SD = Standard Deviation. N ranges from 73 to 438 due to missing data.

# Section 2: Behavioural beliefs

A parallel analysis for the 35 items that measure behavioural beliefs suggested 6 factors. Based on this, a factor analysis (principal axes, followed by Varimax rotation) was carried out, retaining 6 factors.

*Factor 1*

Twenty-four items loaded on the first factor. Ten of these items had a higher loading on factor 2 and one item on factor 6. Fourteen items were further analyzed to decide, which item would be selected to represent the factor. Even though one item was selected we want to emphasize that it became apparent that there was one other item that was included in the final questionnaire. This is further discussed under the subheading “extra items”. We decided to choose the item “feeling better” for the final questionnaire. This is based on several reasons. There was a clear distinction between two group of items. The first group with a factor loading between .72 and .81. A second group with a factor loading between .48 and .60.

This first group included items, such as “better mood”, “feeling better”, “decreasing stress”, “peace of mind”, “feeling good” and “better mental health”. The item “feeling better” showed the highest Pearson correlation compared to the other items. The item “better mental health” was mentioned the most (M = 6.08), which was followed by “feeling good” (M = 5.92) and “feeling better” (M = 5.86). However, we believe that “better mental health” is more associated with feeling less depressed or anxious. In addition, “feeling better” seems more appropriate compared to “feeling good” since the word “better” implies an increase of the feeling. For example, someone might feel bad and as a result of physical activity feel better but that does not mean that the person feels good. The person might simply feel less bad. So, it makes more sense to include the item “feeling better”.

The second group with factor loadings between .48 and .60 represents factor 1 but to a lesser degree due to the lower factor loadings compared to the first group. Even though the first group of items is related to feelings, this is not the completely the case for the second group where it is not clear what item could represent the factor. This is because of several reasons.

One reason is related to items, that have multiple factor loadings, such as the items “better general health” that loaded on three factors and “maintaining health” that loaded on four factors.

The second reason is associated how the item is more relevant for one group of people compared to another group. For example, people who do heavy weightlifting might feel that they have accomplished something (related to the item “feeling like you have accomplished something”) and feel satisfied with themselves (related to the item “self-satisfaction) after a good workout, while this might be less likely the case for those who go for a walk. So, these items would not be representative for factor 1. Also, the item “resting from school/work” might be more relevant for people who are physically active during school/work days compared to those who are physically active during the weekend.

The third reason is that the item “feeling better” could actually represent some of these items. For example, feeling better can mean that you feel more energetic (item “more energy”) but it can also be associated with better sleep (item “better sleep”) and better focus (item “better focus”).

*Factor 2*

Seventeen items loaded on the second factor. Six of these items had a higher loading on factor 1 and one item had a higher loading on factor 6. Ten items were further analyzed to decide, which item would be selected to represent the factor. Even though one item was selected we want to emphasize that it became apparent that there was one other item that was included in the final questionnaire. This is further discussed under the subheading “extra items”. We decided to create a general item “better long-term health (e.g., during the lifetime)” for the final questionnaire. This is based on several reasons. The first reason is that we wanted to choose an item that everyone could respond to. Items such as “an increase in physical strength”, “better posture”, “better work capacity”, “better appearance”, “better self-esteem”, “staying in shape” and “weight gain (e.g., muscle mass)” could leave out people who are not physically active to increase their strength or to have a better posture. In addition, it would leave out people who don’t work. Also, people who are not physically active to have a better appearance, better self-esteem, stay in shape or to gain weight (e.g., muscle mass) would be left out. For example, these kinds of people might be physically active solely for health purposes. The second reason is related to items having multiple factor loadings, which was the cases for the items “preventing health problems” and the item “less health worries”, which have both loadings on two other factors. Finally, the item “longer lifespan” had one cross-loading. The reason why we replaced the item “longer lifespan” with the item “better long-term health (e.g., during the lifetime)” was because a longer lifespan does not imply better health. Someone might live for a long time and experience health problems. That is why we found it more appropriate to put the emphasis on long-term health. We still decided to refer to the lifespan by including “(e.g., during the lifetime)”.

*Factor 3*

Seven items loaded on the third factor. However, one item had a higher loading on factor 5. We believed that this factor represents items that could be seen as the perceived negative effects of physical activity. In this case we decided not to choose a single item to represent the third factor. This was done, because this could otherwise lead to missing important information regarding whether the individual expects (for example) to experience muscle pain. Also, these items could be used to better understand how the perceived negative effects of physical activity are associated with physical activity levels. Lastly, these items could aid in creating an intervention. For example, if individuals are not physically active and this is associated with the belief of feeling uncomfortable or exhausted then this can be targeted.

*Factor 4*

Four items loaded on the fourth factor. However, two items had a higher loading on factor 2. We decided to create the general item, “healthy weight”. This item was created to represent the items “stable weight” and “preventing weight gain”. We believed this item would be more suitable because weight does not perse stay stable and can fluctuate. The item “preventing weight gain” would exclude those who try to lose weight. However, the item “healthy weight” includes weight that can fluctuate (but still can be within healthy norms) but also includes people who try to lose weight.

*Factor 5*

Six items loaded on the fifth factor. However, one item had a higher loading on factor 1, two items had a higher loading on factor 2 and one item had a higher loading on factor 3. The two items that had the highest loading on the factor “Feeling pain (not a normal ‘painful’ feeling of muscle tension/pain)” and “injury/injuries” were both added to the final questionnaire. This was done for the same reason as described under factor 3.

*Factor 6*

Five items loaded on the sixth factor. However, two items had a higher loading on factor 1 and 2 items had a higher loading on factor 2. We decided to include the item “better physical health” in the final questionnaire. This was the only item that had the highest loading on this factor.

*Extra items*

Regarding the first factor it became apparent that it was appropriate to include another item in the questionnaire related to the cognitive evaluation about oneself. This was based on the items “self-satisfaction” and “better self-esteem”. One of these items had a higher loading on factor 2 but this was only .01 higher compared to factor 1. The Pearson correlation showed a strong correlation between these items (r = .76). We chose a general item “being satisfied with myself” to represent these two items because it provides more clarity compared to “self-satisfaction” and “better self-esteem”. For example, being satisfied with yourself could mean that you are proud of yourself, while self-esteem self-satisfaction are more ambiguous.

Regarding factor 2, we realized that an item associated with one’s appearance would be relevant to include in the final questionnaire. The items that were associated with one’s appearance under factor 2 were “better posture”, “better appearance” and “staying in shape”. We chose a general item “better physical shape” because we believed that it covered these three items the best. It can be assumed that a better physical shape consists of having a better posture, a better appearance and it goes beyond staying in shape since the belief is related to a better physical shape.

## Supplementary Table 4: Behavioural beliefs: factor analysis

|  | **1** | **2** | **3** | **4** | **5** | **6** |
| --- | --- | --- | --- | --- | --- | --- |
| 1.     Better mood | **.81** | .20 | -.20 | .10 | .03 | .09 |
| 2.     Feeling better | **.80** | .29 | -.16 | .16 | .05 | .08 |
| 3.     Decreasing stress | **.79** | .18 | -.14 | .10 | .14 | .08 |
| 4.     Peace of mind | **.76** | .25 | -.17 | .27 | .09 | .00 |
| 5.     Feeling good | **.75** | .29 | -.18 | .05 | .02 | .22 |
| 6.     Better mental health | **.72** | .24 | -.18 | .07 | .10 | .29 |
| 7.     Self-satisfaction | **.60** | .39 | -.04 | .21 | .00 | .10 |
| 8.     More energy | **.59** | .36 | -.28 | .07 | .05 | .18 |
| 9.     Better sleep | **.57** | .36 | -.04 | .18 | .16 | .07 |
| 10.  Resting from school/work | **.57** | .20 | -.21 | .20 | .05 | -.16 |
| 11.  Better focus | **.55** | .46 | -.15 | .17 | .29 | -.03 |
| 12.  Better general health | **.54** | .42 | -.05 | .15 | .23 | .49 |
| 13.  Feeling like you have accomplished something | **.53** | .27 | -.02 | .18 | -.11 | .26 |
| 14.  Maintaining health | **.48** | .37 | -.03 | .24 | .38 | .34 |
| 15.  An increase in physical strength | .32 | **.67** | .02 | .19 | .00 | .37 |
| 16.  Better posture | .41 | **.63** | -.09 | .12 | .01 | -.02 |
| 17.  Longer lifespan | .30 | **.63** | .00 | .23 | .20 | .00 |
| 18.  Greater working capacity | .48 | **.60** | -.16 | .22 | .11 | -.01 |
| 19.  Preventing health problems | .32 | **.58** | .03 | .24 | .39 | .17 |
| 20.  Less health worries | .34 | **.56** | -.08 | .19 | .46 | .08 |
| 21.  Better appearance | .35 | **.54** | -.05 | .33 | -.06 | .31 |
| 22.  Better self-esteem | .51 | **.52** | -.10 | .22 | .04 | .19 |
| 23.  Staying in shape | .39 | **.49** | -.06 | .43 | .03 | .26 |
| 24.  Increasing weight (e.g., muscle mass) | .21 | **.48** | .15 | -.06 | -.18 | .09 |
| 25.  Feeling exhausted | -.21 | -.05 | **.78** | -.02 | -.07 | .01 |
| 26.  Feeling uncomfortable | -.22 | -.05 | **.66** | .03 | -.04 | -.09 |
| 27.  Feeling tired | -.16 | -.07 | **.65** | -.04 | -.03 | -.06 |
| 28.  Muscle pain | -.06 | .14 | **.53** | .06 | -.41 | .12 |
| 29.  Less time for other activities (e.g., work, school, other obligations) | -.08 | -.02 | **.43** | -.09 | -.01 | -.03 |
| 30.  Sweating | .07 | .11 | **.33** | .08 | -.17 | .16 |
| 31.  Preventing weight gain | .20 | .23 | .02 | **.66** | .01 | .05 |
| 32.  Stable weight | .21 | .15 | -.03 | **.59** | .08 | .08 |
| 33.  Feeling pain (not a normal “painful” feeling of muscle tension/pain) | -.11 | -.09 | .43 | .03 | **-.44** | -.15 |
| 34.  Injury/injuries | -.03 | -.05 | .28 | -.05 | **-.43** | .00 |
| 35.  Better physical health | .42 | .41 | -.10 | .26 | .09 | **.55** |

The factor loadings in bold represent the highest loadings for each factor.

¨

# Section 3: Subjective norms

## Supplementary Table 5: Injunctive norms

|  | **1** | **2** | **3** | **4** | **5** | **6** | **7** | **8** | **9** |
| --- | --- | --- | --- | --- | --- | --- | --- | --- | --- |
| 1. My mother | -- |  |  |  |  |  |  |  |  |
| 2. My father | .73** | -- |  |  |  |  |  |  |  |
| 3. My brother(s) | .63** | .69** | -- |  |  |  |  |  |  |
| 4. My sister(s) | .63** | .59** | .77** | -- |  |  |  |  |  |
| 5. My friend(s) | .49** | .45** | .65** | .64** | -- |  |  |  |  |
| 6. My colleague(s) | .43** | .42** | .63** | .60** | .81** | -- |  |  |  |
| 7. Other (university) students | .48** | .45** | .62** | .56** | .77** | .86** | -- |  |  |
| 8. My partner | .50** | .57** | .52** | .64** | .69** | .59** | .60** | -- |  |
| 9. My child(ren) | .49** | .55** | .50** | .63** | .74** | .71** | .52** | .73** | -- |
| 10. Relatives (other than parents, siblings and children) | .73** | .67** | .72** | .70** | .69** | .75** | .74** | .58** | .63** |

*Note*. 1 = my mother; 2= my father; 3 = my brother(s); 4 = my sister(s); 5 = my friend(s); 6 = my colleague(s); 7 = other (university) students; 8 = my partner; 9 = my child(ren); 10 = relatives (other than parents/siblings/children. ** = p < 0.01; N varies from 38 To 381 due to missing data.

## Supplementary Table 6: Descriptive norms

|  | **1** | **2** | **3** | **4** | **5** | **6** | **7** | **8** | **9** |
| --- | --- | --- | --- | --- | --- | --- | --- | --- | --- |
| 1. My mother | -- |  |  |  |  |  |  |  |  |
| 2. My father | **.32**** | -- |  |  |  |  |  |  |  |
| 3. My brother(s) | .19** | .24** | -- |  |  |  |  |  |  |
| 4. My sister(s) | .25** | .17* | **.43**** | -- |  |  |  |  |  |
| 5. My friend(s) | .06 | .11 | .21** | .22** | -- |  |  |  |  |
| 6. My colleague(s) | .17* | .17* | .13 | .22* | .37** | -- |  |  |  |
| 7. Other (university) students | .02 | .16* | .11 | .19* | .24** | .38** | -- |  |  |
| 8. My partner | .16* | .06 | .20* | .24** | .32** | -.02 | .09 | -- |  |
| 9. My child(ren) | .15 | .03 | .45** | .56** | .61** | .62** | .36* | .11 | -- |
| 10. Relatives (other than parents, siblings and children) | .28** | .29** | .25** | .24** | .27** | .33** | .36** | .20* | .49** |

*Note*. 1 = my mother; 2= my father; 3 = my brother(s); 4 = my sister(s); 5 = my friend(s); 6 = my colleague(s); 7 = other (university) students; 8 = my partner; 9 = my child(ren); 10 = relatives (other than parents/siblings/children. * = p < .0.05; ** = p < 0.01. N varies from 31 To 407 due to missing data.

## Supplementary Table 7: Motivation to comply

|  | **1** | **2** | **3** | **4** | **5** | **6** | **7** | **8** | **9** |
| --- | --- | --- | --- | --- | --- | --- | --- | --- | --- |
| 1. My mother | -- |  |  |  |  |  |  |  |  |
| 2. My father | .73** | -- |  |  |  |  |  |  |  |
| 3. My brother(s) | .66** | .74** | -- |  |  |  |  |  |  |
| 4. My sister(s) | .76** | .64** | .80** | -- |  |  |  |  |  |
| 5. My friend(s) | .57** | .56** | .59** | .62** | -- |  |  |  |  |
| 6. My colleague(s) | .39** | .48** | .60** | .59** | .62** | -- |  |  |  |
| 7. Other (university) students | .39** | .44** | .50** | .48** | .62** | .73** | -- |  |  |
| 8. My partner | .57** | .54** | .53** | .63** | .60** | .45** | .37** | -- |  |
| 9. My child(ren) | .46** | .45** | .69** | .76** | .57** | .59** | .49** | .81** | -- |
| 10. Relatives (other than parents, siblings and children) | .50** | .49** | .62** | .53** | .54** | .63** | .55** | .36** | .48** |

*Note*. 1 = my mother; 2= my father; 3 = my brother(s); 4 = my sister(s); 5 = my friend(s); 6 = my colleague(s); 7 = other (university) students; 8 = my partner; 9 = my child(ren); 10 = relatives (other than parents/siblings/children. * = p < .0.05; ** = p < 0.01. N varies from 40 To 410 due to missing data.

# Section 4: Control beliefs

A parallel analysis for the 48 items that measure control beliefs and their perceived power to influence behaviour suggested 12 factors. Based on this a factor analysis was carried out for 12 factors. The data showed a clear distinction between most factors (compared to the factors that measure behavioural beliefs) and it was decided to label each factor separately.

Only under factor 3 and factor 6 it became apparent that other items would be relevant to include. In addition, one of the items did not have a loading on any factor but was also included in the final questionnaire. Excluding these items could lead to missing important information. This is further explained under the subheading “extra items”.

*Factor 1*

Fifteen items loaded on factor 1. However, two of these items had a higher loading on factor 2, one item a higher loading on factor 5 and two items on factor 6. Ten items were further analyzed. We believed that the first factor represented the items that are associated with how someone feels, because energy levels, mood, stress levels and motivations are related to feelings. Therefore, the item “how I am feeling” was included in the final questionnaire.

*Factor 2*

Seven items loaded factor 2. We believed that the second factor represented the items that are associated with time, because having obligations and other priorities can mean that someone has less time to be physically active. Therefore, the item “do I have time or not” was included in the final questionnaire.

*Factor 3*

Nine items loaded on factor 3. However, one item had a higher loading on factor 6, one item on factor 8 and one item on factor 11. Six items were further analyzed. Even though one item was selected we want to emphasize that it became apparent that there were two other items that were included in the final questionnaire. This is further discussed under the subheading “extra items”.

The items “lack of necessary equipment” (.44) and “having the necessary equipment” (.38) clearly had the lowest factor loadings compared to the other four items. We performed a Pearson correlation to assess the relationship between the four other items, which showed that the items “whether there is a gym nearby” and “there is no gym nearby” (r = .69) and “whether the gym is open or not” and “whether there is a gym nearby” (r = .83) had the highest correlation. The Pearson correlation showed that the item “whether there is a gym nearby” had a higher correlation with the item “whether the gym open or not” compared to the item “there is no gym nearby”. We chose the item “whether there is a gym nearby” to represent factor 3. However, we decided to adjust the item since “gym” is too specific. So, the final item that was included in the questionnaire was “the distance to the place to be physically active (e.g., gym, swimming pool etc.)” to put more emphasis on other places where one can be physically active.

*Factor 4*

Four items loaded on factor 4. The fourth factor appeared to be best represented by one general item, “My social environment (e.g., there are no people around me who are physically active, when I do not have someone to do it with)” since the four items were related to the social environment. For example, having someone to be physically active with or having people around who are physically active is related to the social environment.

*Factor 5*

Four items loaded on factor 5. However, one item had a higher loading on factor 1. Three items were further analyzed. The two items “how fast I see the results” (.74) and “not seeing the results fast enough” (.70) had a higher loading compared to the item “my other goals” (.31). This showed that these two items (“how fast I see the results” and “not seeing the results fast enough”) were a better representative for factor 5. Since the final questionnaire investigates the control beliefs instead of perceived power, the wording of the item “how fast I see the results” suits better and was therefore selected to represent factor 5. For example, “my time to be physically active depends on how fast I see the results” suits better than “my time to be physically active depends on not seeing the results fast enough”.

*Factor 6*

Five items loaded on factor 6. However, one item had a higher loading on factor 12. Four items were further analyzed. We performed a Pearson correlation to assess the relationship between the four items, which showed that the items “the weather” and “bad weather” (r = .79) and “where I live” and “the place where I live” (r = .60) had +the highest correlation. In addition, the items “the weather” (.71) and “bad weather” (.67) had a higher factor loading compared to the items “the place where I live” (.42) and “where I live” (.36). We finally decided to include the item “the weather” in the final questionnaire because of its factor loading compared to the other items and due to its wording, which suits the measurement of control beliefs. For example, “my time to be physically active depends on the weather” suits better than “my time to be physically active depends on bad weather”.

*Factor 7*

Two items loaded on factor 7. We decided to choose the item “my children” since both items that loaded on this factor were “my children”. However, after closer analysis of the two items “my children” it became apparent that these items contain many missing values (at least 85%). For the purpose of keeping the questionnaire short we decided to omit this item.

*Factor 8*

Two items loaded on factor 8. We decided to choose the item “the Covid-19 situation” since both items that loaded on this factor were “the Covid-19 situation”.

*Factor 9*

Two items loaded on factor 9. The ninth factor appeared to be best represented by one general item, “transportation possibilities (e.g., go with the bus to the swimming pool)” since the two items were related to transportation. The reason why the general item was chosen is because the two items were too specific, focusing on having a car but not taking into account other transportation possibilities, such a bus or tram.

*Factor 10*

Two items loaded on factor 10. The tenth factor appeared to be best represented by the item “my planning skills” since both items that loaded on this factor were “my planning skills”.

*Factor 11*

Two items loaded on factor 11. The eleventh factor appeared to be best represented by the item, “my financial situation”, since both items that loaded on this factor were “my financial situation”.

*Factor 12*

Four items loaded on factor 12. However, one item had a higher loading on factor 1. Three items were further analyzed. Pearson correlation showed a value of .47 between the items “my health” and “my health problems”. However, the correlation between these two items and the item “living in an unsafe environment” had a value of .27 and .14. We decided to choose the item “my health” to represent factor 12 not only because of the correlation value but also because the wording “my health” is more appropriate than “my health problems”. For example, “my time to be physically active depends on my health” suits better than “my time to be physically active depends on my health problems”.

*Extra items*

Related to factor 3, two other items were included in the final questionnaire. The first item is “the possibilities to use to place to be physically active (e.g., tennis course is closed)”. This item was chosen based on the items “whether the gym is open or not” and “the gym (or other sports facility) is closed”, which had a correlation of *r* = .69. However, the items were too specific and excludes people who don’t use the gym or sports facilities. The second item is “having the necessary equipment (e.g., yoga mat, dumbbells)”. This item was chosen because it includes people who belief they need certain equipment to be physically active. We included the examples (yoga mat and dumbbells) for clarity purposes of the item. Related to factor 6, one other item was included in the final questionnaire, “the place where I live”, because the place where someone lives can influence their physical activity levels. For example, people who live close to a forest might be more likely to go for a walk compared to people who live in the city. Or people who live in a place where there are no proper walking streets might be less likely to go for a walk compared to people who have proper walking streets. One of the items did not have a loading on any factor based on the analysis, “whether the environment is safe”. However, we found it necessary to include this item since the environment is included in some of the items, for example “my social environment” and “the distance to the place to be physically active (e.g., gym, swimming pool etc.)” but safety was not included even though this could play a role. For example, someone who lives in an unsafe environment might be less likely to go for a walk compared to someone who lives in a safe environment. We adjusted the item to put more emphasis on the place where the person lives, “the safety of the place where I live”.

## Supplementary Table 8: Control beliefs and their perceived power to influence behaviour: factor analysis

|  | 1 | 2 | 3 | 4 | 5 | 6 | 7 | 8 | 9 | 10 | 11 | 12 |
| --- | --- | --- | --- | --- | --- | --- | --- | --- | --- | --- | --- | --- |
| 1. My energy levels | **.77** | .16 | .09 | .06 | .05 | .07 | .04 | .00 | .01 | .06 | .03 | .09 |
| 1. My mood | **.76** | .21 | -.01 | .08 | .18 | .18 | .03 | .02 | .02 | .06 | .01 | -.02 |
| 1. Low energy levels* | **.76** | .20 | .08 | .15 | .10 | .08 | .00 | -.06 | .05 | .04 | .04 | .19 |
| 1. My stress levels | **.72** | .22 | .04 | .07 | .13 | .15 | .07 | .07 | .06 | .14 | .02 | -.02 |
| 1. How I am feeling | **.71** | .18 | .02 | .03 | -.06 | .05 | .02 | .06 | .00 | .10 | .12 | .12 |
| 1. How motivated I am | **.70** | .18 | -.04 | .10 | .12 | .11 | -.05 | .08 | -.03 | .09 | .05 | -.03 |
| 1. Being in a bad mood* | **.69** | .19 | .09 | .12 | .30 | .19 | .04 | -.04 | .11 | .06 | -.02 | .10 |
| 1. Low motivation* | **.68** | .21 | .02 | .22 | .22 | .10 | -.02 | -.02 | .00 | .03 | .07 | .11 |
| 1. Feeling bad* | **.67** | .17 | .03 | .09 | -.01 | .07 | .00 | -.04 | .07 | .02 | .03 | .33 |
| 1. High stress levels* | **.65** | .25 | .09 | .14 | .22 | .11 | .11 | -.02 | .09 | .10 | .02 | .06 |
| 1. A lot of work or school related obligations* | .19 | **.88** | .07 | .10 | .05 | .02 | .02 | -.01 | .04 | .10 | .04 | .12 |
| 1. How many work or school commitments I have | .24 | **.80** | .06 | .12 | -.02 | .07 | .01 | .06 | .07 | .07 | .04 | .07 |
| 1. Having many obligations* | .17 | **.80** | .04 | .10 | .06 | .08 | .04 | .02 | .01 | .16 | .02 | .10 |
| 1. The amount of obligations I have | .22 | **.78** | .01 | .07 | .02 | .06 | .04 | .09 | .06 | .12 | .02 | .06 |
| 1. Not having time* | .24 | **.73** | .10 | .07 | .06 | .11 | .01 | -.02 | -.02 | .04 | .05 | .06 |
| 1. Whether I have time or not | .34 | **.61** | .03 | -.01 | .03 | .14 | .00 | .05 | -.05 | .01 | .00 | -.08 |
| 1. Other priorities that I have* | .30 | **.54** | -.06 | .01 | .23 | .07 | .03 | .07 | .05 | .11 | -.01 | .18 |
| 1. Whether the gym is open or not | .04 | .04 | **.84** | -.04 | -.04 | -.10 | -.01 | .11 | .00 | -.03 | .16 | -.05 |
| 1. Whether there is a gym nearby | .03 | .08 | **.82** | -.03 | -.02 | -.04 | .03 | .13 | .01 | .01 | .10 | -.06 |
| 1. There is no gym nearby* | .05 | .04 | **.76** | .07 | .09 | .09 | .03 | .04 | .18 | .01 | .04 | .01 |
| 1. The gym (or other sports) facility is closed* | -.06 | .01 | **.71** | .05 | -.06 | -.04 | .01 | .12 | .13 | .01 | .06 | .02 |
| 1. Lack of necessary equipment* | .12 | .02 | **.44** | .15 | .28 | .16 | -.04 | .09 | .20 | .16 | .12 | .11 |
| 1. Having the necessary equipment | .24 | .02 | **.38** | .13 | .28 | .10 | -.04 | .09 | .20 | .18 | .11 | .04 |
| 1. When I don't have anyone to do it with (e.g., play sports, exercise with someone, etc.)* | .14 | .11 | .05 | **.85** | .05 | .09 | .05 | .02 | .09 | -.05 | .06 | .12 |
| 1. Whether I have someone to do it with or not (e.g., play sports, exercise with someone, etc.) | .15 | .09 | .02 | **.80** | .07 | .04 | -.01 | .03 | .07 | -.05 | .06 | .03 |
| 1. When there are no people around me who are physically active* | .19 | .13 | .08 | **.62** | .17 | .12 | .14 | .12 | .08 | .18 | .03 | -.05 |
| 1. Whether people around me are physically active | .21 | .07 | .06 | **.50** | .15 | .09 | .08 | .12 | .07 | .25 | .02 | -.10 |
| 1. How fast I see the results | .28 | .10 | .05 | .17 | **.74** | .09 | .03 | .07 | .05 | .01 | .10 | .06 |
| 1. Not seeing the results fast enough* | .34 | .09 | .03 | .16 | **.70** | .08 | .02 | .02 | .03 | -.03 | .06 | .10 |
| 1. My other goals | .21 | .29 | .04 | .08 | **.31** | .05 | .07 | .16 | .12 | .26 | .02 | .06 |
| 1. The weather | .35 | .16 | -.13 | .10 | .01 | **.71** | .04 | .05 | .03 | -.01 | .14 | -.04 |
| 1. Bad weather* | .33 | .22 | -.04 | .09 | .03 | **.67** | .08 | .06 | .03 | -.04 | .13 | .00 |
| 1. The place where I live* | .13 | .09 | .35 | .23 | .23 | **.42** | -.03 | -.03 | .21 | .03 | -.01 | .18 |
| 1. Where I live | .19 | .10 | .27 | .16 | .20 | **.36** | -.01 | .00 | .10 | .06 | .03 | .03 |
| 1. Whether the environment is safe | .22 | .08 | -.04 | .05 | .05 | .27 | .02 | .19 | -.06 | .27 | -.04 | .25 |
| 1. My children | .04 | .06 | .00 | .08 | .00 | .02 | **.88** | .05 | .02 | .02 | -.04 | .00 |
| 1. My children* | .06 | .03 | .01 | .07 | .04 | .03 | **.87** | .03 | -.02 | .04 | -.01 | .08 |
| 1. The Covid-19 situation | .01 | .09 | .24 | .10 | .09 | .05 | .04 | **.85** | .09 | .03 | .08 | .05 |
| 1. The Covid-19 situation* | -.01 | .09 | .31 | .12 | .04 | .07 | .06 | **.78** | .07 | .03 | .08 | .15 |
| 1. Not having a car* | .06 | .02 | .22 | .12 | .05 | .07 | .05 | .06 | **.81** | .01 | .09 | .06 |
| 1. Whether I have a car or not (e.g., to go to the gym) | .06 | .08 | .22 | .13 | .07 | .04 | -.05 | .07 | **.73** | .02 | .12 | -.02 |
| 1. My planning skills | .13 | .16 | .06 | .03 | -.04 | .00 | .04 | .03 | .03 | **.73** | .08 | .04 |
| 1. My planning skills* | .17 | .29 | .03 | .10 | .08 | .02 | .00 | -.03 | .00 | **.68** | .03 | .11 |
| 1. My financial situation | .14 | .07 | .27 | .07 | .08 | .11 | -.03 | .13 | .11 | .08 | **.82** | -.01 |
| 1. My financial situation* | .13 | .08 | .37 | .14 | .14 | .13 | -.04 | .05 | .18 | .05 | **.73** | .09 |
| 1. My health problems* | .16 | .15 | .01 | .02 | .11 | -.04 | .07 | .03 | .04 | -.01 | .01 | **.62** |
| 1. My health | .29 | .12 | -.05 | -.03 | -.03 | .02 | .04 | .12 | .00 | .14 | .08 | **.45** |
| 1. Living in an unsafe environment* | .08 | .07 | .04 | .06 | .17 | .32 | -.05 | .07 | .00 | .16 | -.04 | **.37** |

* = the items that measure perceived power to influence behaviour. The factor loadings in bold represent the highest loadings for each factor.
