# Supplementary material for: Toward Comprehensive Assessment of Beliefs and Attitudes Related to Physical Activity in Young Adults: Pilot Study
Source: JMIR Form Res. 2025 Oct 16;9:e69094. doi: 10.2196/69094 (PMC12576300; doi:10.2196/69094)
Supplement: Multimedia Appendix 7 [file formative_v9i1e69094_app7.docx]

Towards comprehensive assessment of beliefs and attitudes related to physical activity motivation in young adults: Bottom-up construction of a TPB-based questionnaire

Theodorus D.B. Noordover^1,2^, Aave Hannus^1,2^, Kenn Konstabel^2,1^

^1^Institute of Psychology, University of Tartu, Estonia

^2^National Institute for Health Development, Tallinn, Estonia

Table of contents

[Introductory note 2](#_Toc210051375)

[Behavioural beliefs 2](#_Toc210051376)

[Kaiser-Meyer-Olkin criterion and Bartlett’s test 2](#_Toc210051377)

[Skewness and kurtosis 3](#_Toc210051378)

[Dimensionality 4](#_Toc210051379)

[Positive behavioural beliefs 5](#_Toc210051380)

[Table X1. Positive behavioural beliefs: factor loadings and IRT parameters 9](#_Toc210051381)

[Negative behavioural beliefs 9](#_Toc210051382)

[Acceptance 10](#_Toc210051383)

[Acceptance and negative behavioural beliefs 12](#_Toc210051384)

[Injunctive norms 14](#_Toc210051385)

[Descriptive norms 18](#_Toc210051386)

[Motivation to comply 22](#_Toc210051387)

[Normative beliefs´ 26](#_Toc210051388)

[Table X3. Normative beliefs: EFA loadings and IRT parameters 26](#_Toc210051389)

[Control beliefs 27](#_Toc210051390)

[Table X4. Control beliefs: EFA loadings of the shortened version 31](#_Toc210051391)

[Prediction of physical activity 33](#_Toc210051392)

[Table X5. Correlations with PA 34](#_Toc210051393)

[Table X6. Multiple regression analysis, predicting PA from TPB+acceptance 35](#_Toc210051394)

# Introductory note

This file is made using Quarto and contains R code with unedited results (changes after “knitting” consisted of adding this note and a few minor formatting changes). The R code used the add-on packages psych, EFAtools, and flextable (for table formatting) plus some custom functions. The data set as well as custom functions are available at <https://osf.io/u7ytc/>

# Behavioural beliefs

## Kaiser-Meyer-Olkin criterion and Bartlett’s test

BB <- grabit(c("BBN", "BBP"))
KMO(BB)

ℹ 'x' was not a correlation matrix. Correlations are found from entered raw data.

── Kaiser-Meyer-Olkin criterion (KMO) ──────────────────────────────────────────

✔ The overall KMO value for your data is marvellous.
 These data are probably suitable for factor analysis.

 Overall: 0.953

 For each variable:
 BBN1 BBN2 BBN3 BBP4 BBP5 BBP6 BBP7 BBP8 BBP9 BBN10 BBN11 BBN12 BBN13
0.792 0.830 0.861 0.978 0.957 0.957 0.956 0.967 0.976 0.722 0.900 0.860 0.867
BBP14 BBP15 BBP16 BBP17 BBP18 BBP19 BBP20 BBP21 BBP22 BBP23 BBN24 BBP25 BBP26
0.971 0.967 0.962 0.970 0.967 0.963 0.954 0.953 0.960 0.958 0.844 0.955 0.965
BBP27 BBP28 BBN29 BBP30 BBP31 BBP32 BBP33 BBP34 BBP35
0.954 0.907 0.924 0.923 0.959 0.963 0.964 0.950 0.970

BARTLETT(BB)

ℹ 'x' was not a correlation matrix. Correlations are found from entered raw data.

✔ The Bartlett's test of sphericity was significant at an alpha level of .05.
 These data are probably suitable for factor analysis.

 𝜒²(595) = 10651.05, p < .001

## Skewness and kurtosis

Fabrigar LR, Wegener DT, eds. Exploratory Factor Analysis. Oxford University Press; 2012.

“Substantial distortions in ML parameter estimates did not emerge until measured variables had an absolute value of skew of two or greater and an absolute value of kurtosis of seven or greater. Thus, data sets with skew and kurtosis values substantially smaller than these guidelines are unlikely to present problems for ML EFA, whereas data sets at or above these values might be problematic.” (citing Curran et al., 1996)

In our analysis, none of the values exceed these thresholds.

k <- kurtosi(BB)
s <- skew(BB)
data.frame(item=getEng(BB), skewness=s %r% 2, kurtosis = k %r% 2 ) |> qflextable()

| item | skewness | kurtosis |
| --- | --- | --- |
| Injury/injuries | 0.97 | 0.35 |
| Less time for other activities | 0.02 | -0.95 |
| Feeling tired | 0.23 | -0.83 |
| Feelings like you have accomplished something | -1.13 | 0.59 |
| Better physical health | -1.93 | 4.22 |
| Better mental health | -1.54 | 1.96 |
| Better appearance | -0.84 | -0.08 |
| Better sleep | -0.90 | 0.28 |
| Feeling good | -1.31 | 1.72 |
| Sweating | -0.91 | 0.19 |
| Feeling uncomfortable | 0.52 | -0.71 |
| Feeling pain (not a normal "painful" feeling of muscle tension/pain) | 1.06 | 0.36 |
| Feeling exhausted | 0.43 | -0.59 |
| More energy | -0.39 | -0.29 |
| Better mood | -0.81 | 0.28 |
| Better overall health | -1.33 | 1.70 |
| Decreasing stress | -1.07 | 0.56 |
| Maintaining health | -1.01 | 0.24 |
| Better focus | -0.37 | -0.59 |
| Less health worries | -0.53 | -0.34 |
| An increase in physical strength | -1.28 | 1.45 |
| Preventing health problems | -0.63 | -0.34 |
| Better self-esteem | -1.17 | 0.81 |
| Muscle pain | -0.14 | -0.81 |
| Self-satisfaction | -0.81 | 0.25 |
| Resting from school/work | -0.85 | -0.13 |
| Staying in shape | -1.05 | 0.62 |
| Preventing weight gain | -0.58 | -0.50 |
| Weight gain (e.g., muscle mass) | 0.09 | -0.88 |
| Stable weight | -0.42 | -0.31 |
| Peace of mind | -0.82 | 0.19 |
| Feeling better | -1.15 | 1.07 |
| Better posture | -0.64 | -0.30 |
| Longer lifespan | -0.60 | -0.17 |
| Greater work capacity | -0.47 | -0.51 |

## Dimensionality

# PARALLEL(BB, n_datasets=100, eigen_type = "EFA", decision_rule = "mean")
Parallel(BB)

ℹ 'x' was not a correlation matrix. Correlations are found from entered raw data.

Parallel Analysis performed using 100 simulated random data sets
Eigenvalues were found using EFA

Decision rule used: means

── Number of factors to retain according to ────────────────────────────────────

◌ EFA-determined eigenvalues: 6


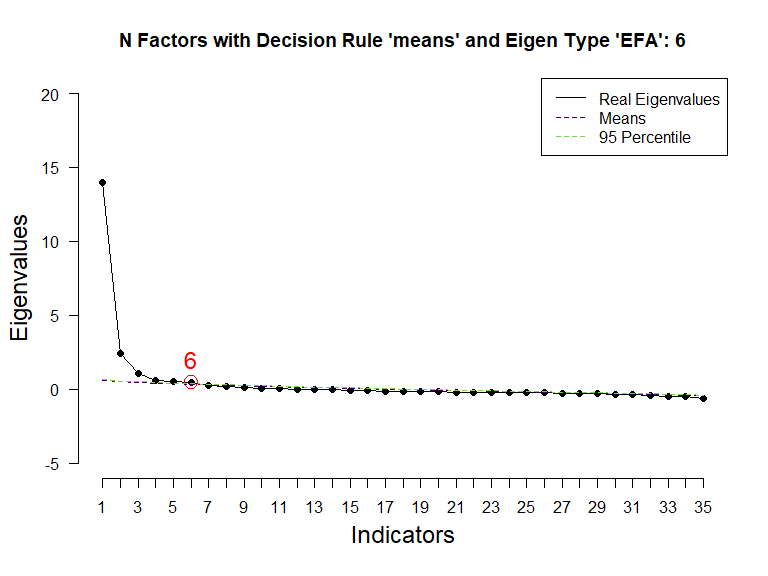


The matrix of factor loadings (6 factors) is presented in Supplementary Table 4.

# Positive behavioural beliefs

BBP <- grabit("BBP")
BBPS <- grabit("BBP", Short = TRUE, Order = "short")
ac1 <- avgcor(BBP) %r% 2
ao1 <- alphaomega(BBP) %r% 2
ac <- avgcor(BBPS) %r% 2
ao <- alphaomega(BBPS) %r% 2

The average correlation between items is 0.51 (larger than in the case of negative beliefs). This may mean that the negative beliefs are more different from each other, or that in our sample of items, negative beliefs were underrepresented in comparison to positive beliefs. The Cronbach’s alpha for the long version (26 items) was 0.96 and McDonald’s omega 0.97.

Based on exploratory factor analysis, we constructed a short version of the scale, including one to two indicators from each of the 4 factors. (Note that in the shortened version, some items were rephrased to either improve readability or generalizability, or to combine several items. For example, the new item “better long-term health” merged the old items “longer lifespan” and “preventing health problems”. In this case, the average of two original items was used (after rounding to integer, to preserve the original number of categories). Therefore, the results are only an approximation of what would happen if the items were presented to the participants exactly as in the final questionnaire.

The average correlation between the items in the shortened version was 0.5, with Cronbach alpha of 0.86 and McDonalds’ omega of 0.9.

efa.bbps1 <- doefa(BBPS,1)
exp <- 100*attr(efa.bbps1, "exp") %r% RT
expn <- 100*attr(efa.bbps1, "expnext")[2] %r% RT
corlosh <- cor.test(rowSums(BBP), rowSums(BBPS))

qflextable(efa.bbps1)

| var | F1 |
| --- | --- |
| Feeling better | 0.76 |
| Better physical health | 0.77 |
| Preventing health problems+Longer lifespan | 0.74 |
| Stable weight | 0.47 |
| Staying in shape | 0.80 |
| Self-satisfaction | 0.74 |

The first factor explained 52% of variance whereas the next unrotated factor explained 4% of variance. The correlation between sumscores of long and short versions was 0.94 (p < 0.00001). This indicates that for the purpose of assessing the positivity of attitude towards PA, the shortened scale provides a reasonable approximation.

We next examined the data with IRT (graded response model) to compare the informativeness of items at different levels of the latent variable. Instead of presenting 6 difficulty parameters separately, we use the IRF-based location index, based on Ali, Chang & Anderson (2015) and implemented in R’s mirtpackage (Chalmers, 2012). Roughly, this index refers to the value of the latent variable where the expected response of is at the scale midpoint.

Ali, U. S., Chang, H.-H., & Anderson, C. J. (2015). Location indices for ordinal polytomous items based on item response theory (Research Report No. RR-15-20). Princeton, NJ: Educational Testing Service. http://dx.doi.org/10.1002/ets2.12065

Chalmers, R., P. (2012). mirt: A Multidimensional Item Response Theory Package for the R Environment. Journal of Statistical Software, 48(6), 1-29. doi:10.18637/jss.v048.i06

tab1 <- coefirt1(mirt.bbp3, labels=getEng(BBP))
tab1 <- data.frame(id=rownames(tab1), short = subset(K, section %in% "BBP")$short, tab1)
tab1 <- tab1[order(tab1$location),]
qflextable(tab1)

| id | short | X. | F1 | discrimination | location |
| --- | --- | --- | --- | --- | --- |
| BBP5 | 2 | Better physical health | 0.87 | 3.04 | -1.71 |
| BBP21 |  | An increase in physical strength | 0.80 | 2.24 | -1.56 |
| BBP4 |  | Feelings like you have accomplished something | 0.70 | 1.67 | -1.54 |
| BBP9 |  | Feeling good | 0.88 | 3.17 | -1.50 |
| BBP6 |  | Better mental health | 0.89 | 3.27 | -1.49 |
| BBP16 |  | Better overall health | 0.89 | 3.41 | -1.46 |
| BBP30 | 4 | Stable weight | 0.48 | 0.94 | -1.46 |
| BBP25 | 6 | Self-satisfaction | 0.82 | 2.47 | -1.45 |
| BBP15 |  | Better mood | 0.85 | 2.74 | -1.42 |
| BBP27 | 5 | Staying in shape | 0.82 | 2.46 | -1.38 |
| BBP32 | 1 | Feeling better | 0.92 | 3.96 | -1.38 |
| BBP17 |  | Decreasing stress | 0.85 | 2.75 | -1.27 |
| BBP22 | 3 | Preventing health problems | 0.78 | 2.11 | -1.25 |
| BBP18 |  | Maintaining health | 0.85 | 2.70 | -1.22 |
| BBP26 |  | Resting from school/work | 0.66 | 1.51 | -1.19 |
| BBP8 |  | Better sleep | 0.78 | 2.10 | -1.18 |
| BBP23 |  | Better self-esteem | 0.86 | 2.90 | -1.14 |
| BBP31 |  | Peace of mind | 0.88 | 3.18 | -1.13 |
| BBP7 |  | Better appearance | 0.76 | 1.97 | -1.11 |
| BBP33 |  | Better posture | 0.76 | 1.99 | -1.02 |
| BBP28 |  | Preventing weight gain | 0.54 | 1.09 | -1.01 |
| BBP34 | 3 | Longer lifespan | 0.73 | 1.83 | -0.97 |
| BBP20 |  | Less health worries | 0.77 | 2.03 | -0.92 |
| BBP14 |  | More energy | 0.79 | 2.16 | -0.82 |
| BBP19 |  | Better focus | 0.79 | 2.20 | -0.76 |
| BBP35 |  | Greater work capacity | 0.82 | 2.40 | -0.75 |

One can see from the table, that the location parameters are all negative: the scale discriminates well between low levels of positive beliefs related to PA, but is less informative about higher levels. This is perhaps especially true for the shortened scale. Therefore, one might consider adding some items from the lower end of the distribution. Note, though, that generating items with varying levels of location parameter was not among the aims of the present study, and future studies might do this more systematically.

add3 <- tail(rownames(tab1),3)
BBPS2 <- cbind(BBPS, D[, add3])
attr(BBPS2, "Eng") <- c(getEng(BBPS), setNames(subset(K, newname %in% add3)$eng, add3))
corlosh2 <- cor.test(rowSums(BBPS2), rowSums(BBP))

After adding 3 items with the highest (least negative) location parameters, the correlation between shortened and full scale becomes a bit larger (0.98).

The purpose, for now, is not to describe the correlational structure of positive behavioural beliefs but to describe it as a unidimensional scale. (For more nuanced information, we recommend using single items. For constructing subscales of positive beliefs, additional studies would be needed.)

efa.bbps1 <- doefa(BBPS,1)
exp <- 100*attr(efa.bbps1, "exp") %r% RT
expn <- 100*attr(efa.bbps1, "expnext")[2] %r% RT
mirtcoef <- coefirt1(mirt.bbps)
qft <- data.frame(efa.bbps1, mirtcoef[,-1])
qflextable(qft)

| var | F1 | F1.1 | discrimination | location |
| --- | --- | --- | --- | --- |
| Feeling better | 0.76 | 0.85 | 2.78 | -1.53 |
| Better physical health | 0.77 | 0.88 | 3.19 | -1.75 |
| Preventing health problems+Longer lifespan | 0.74 | 0.78 | 2.15 | -0.97 |
| Stable weight | 0.47 | 0.53 | 1.05 | -1.33 |
| Staying in shape | 0.80 | 0.86 | 2.90 | -1.35 |
| Self-satisfaction | 0.74 | 0.81 | 2.38 | -1.50 |

efa.bbps2 <- doefa(BBPS2,1)
exp <- 100*attr(efa.bbps2, "exp") %r% RT
expn <- 100*attr(efa.bbps2, "expnext")[2] %r% RT
mirtcoef <- coefirt1(mirt.bbps2)
qft <- data.frame(efa.bbps2, mirtcoef[,-1])
qft$var[3] <- "Better long-term health *"
names(qft)[1:3] <- c(" ", "EFA loading", "IRT loading")

### Table X1. Positive behavioural beliefs: factor loadings and IRT parameters

|  | EFA loading | IRT loading | discrimination | location |
| --- | --- | --- | --- | --- |
| Feeling better | 0.78 | 0.87 | 3.07 | -1.49 |
| Better physical health | 0.74 | 0.87 | 2.94 | -1.77 |
| Better long-term health * | 0.78 | 0.82 | 2.45 | -0.91 |
| Stable weight | 0.44 | 0.49 | 0.96 | -1.43 |
| Staying in shape | 0.76 | 0.83 | 2.55 | -1.39 |
| Self-satisfaction | 0.73 | 0.80 | 2.30 | -1.50 |
| More energy | 0.70 | 0.76 | 1.97 | -0.85 |
| Better focus | 0.74 | 0.78 | 2.14 | -0.77 |
| Greater work capacity | 0.80 | 0.84 | 2.66 | -0.73 |

# Negative behavioural beliefs

BBN <- grabit("BBN")

In the final version, we decided to omit one item (weight gain, e.g. Weight gain (e.g., muscle mass)), as it is not clearly negative, has the lowest correlation with other negative items, is counterintuitive for many respondents, and is mostly relevant for only a specific target group (which, however, is rarely the target of PA interventions).

The average correlation among the remaining 8 items was 0.3, with Cronbach alpha of 0.78 and McDonalds’ omega of 0.83.

The table below shows EFA (principal axes) factor loadings along with IRT (graded response model) parameters: discrimination, generalized location (instead of 6 different thresholds), and standardized factor loadings (F1).

# negative behavioral beliefs, EFA + MIRT
efa.bbns1 <- doefa(BBNS,1)
exp <- 100*attr(efa.bbns1, "exp") %r% RT
expn <- 100*attr(efa.bbns1, "expnext")[2] %r% RT
mirtcoef <- coefirt1(mirt.bbns)
qft.bbn <- data.frame(efa.bbns1, mirtcoef[,-1])
names(qft.bbn)[1:3] <- c(" ", "EFA loading", "IRT loading")

The first factor explained 32% of variance whereas the next unrotated factor explained 6% of variance.

# Acceptance

A <- grabit("A")
ae <- attr(A, "Eng")[c(6,8,1,3, 7, 4, 5, 2)]
A <- A[,c(6,8,1,3, 7, 4, 5, 2)]
attr(A, "Eng") <- ae
#PARALLEL(A, n_datasets=100, eigen_type = "EFA", decision_rule = "mean")
KMO(A)

── Kaiser-Meyer-Olkin criterion (KMO) ──────────────────────────────────────────

✔ The overall KMO value for your data is middling.
 These data are probably suitable for factor analysis.

 Overall: 0.78

 For each variable:
 A6 A8 A1 A3 A7 A4 A5 A2
0.695 0.852 0.758 0.782 0.779 0.731 0.802 0.846

BARTLETT(A)

✔ The Bartlett's test of sphericity was significant at an alpha level of .05.
 These data are probably suitable for factor analysis.

 𝜒²(28) = 873.95, p < .001

k <- kurtosi(A)
s <- skew(A)
data.frame(item=getEng(A), skewness=s %r% 2, kurtosis = k %r% 2, complete.obs=sapply(A, \(x) sum(!is.na(x))) ) |> qflextable()

| item | skewness | kurtosis | complete.obs |
| --- | --- | --- | --- |
| Feeling pain [...] | 0.56 | -0.81 | 447 |
| Muscle pain | -0.94 | 0.04 | 447 |
| Injury/injuries | 0.33 | -1.00 | 447 |
| Feeling tired | -0.51 | -0.58 | 447 |
| Feeling exhausted | 0.05 | -0.87 | 447 |
| Sweating | -1.45 | 1.39 | 447 |
| Feeling uncomfortable | -0.26 | -0.74 | 447 |
| Less time for other activities [...] | -0.14 | -1.11 | 447 |

Parallel(A)

Parallel Analysis performed using 100 simulated random data sets
Eigenvalues were found using EFA

Decision rule used: means

── Number of factors to retain according to ────────────────────────────────────

◌ EFA-determined eigenvalues: 4


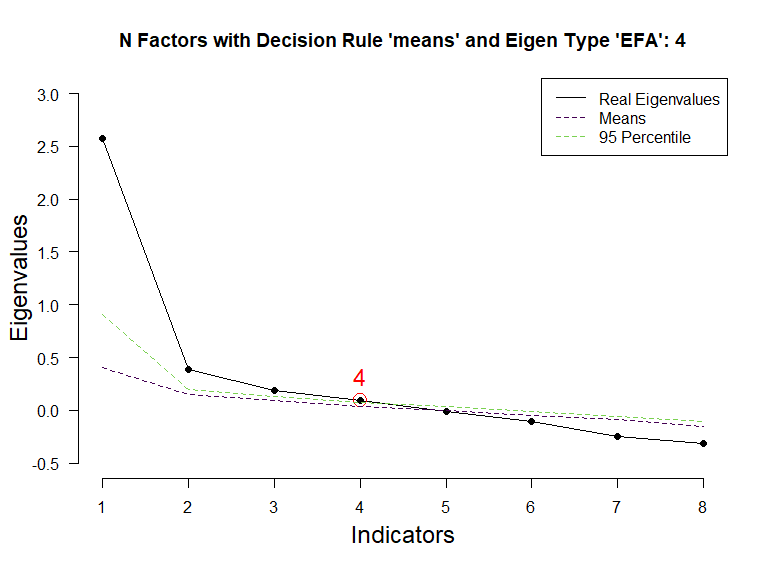


Parallel analysis suggests 4 factors. Even so, we will proceed with unidimensional analyses because the second factor is hard to interpret (only two loadings over 0.3: sweating 0.353 and feeling pain -0.853).

efa.a <- doefa(A,1)
exp <- 100*attr(efa.a, "exp") %r% 3
expn <- 100*attr(efa.a, "expnext")[2] %r% 3

The first factor explains 32.1% of the variance whereas the second factor adds 13%.

ao <- alphaomega(A) %r% 2

The average correlation among the remaining 8 items was 0.31, with Cronbach alpha of 0.79 and McDonalds’ omega of 0.85.

In the next table, EFA loadings are presented as well as IRT discrimination and generalized location parameters. In the last (rightmost) column, there are standardized factor loadings from IRT (GRM) model. Note that IRT factor loadings correspond well to the EFA ones, except for being a little bit higher in most cases. The generalized location parameter varies from -1.68 (sweating) to 0.96 (pain).

mirtcoef <- coefirt1(mirt.a)
qft.acc <- data.frame(efa.a, mirtcoef[,-1])
names(qft.acc)[1:3] <- c(" ", "EFA loading", "IRT loading")

# Acceptance and negative behavioural beliefs

cor.an <- psych::corr.test(A, BBNS)
d <- diag(cor.an$r) %r% 3

The correlations between negative behavioural beliefs and acceptance of the corresponding outcome were generally low, varying from -0.148 to 0.261.

tab2 <- data.frame(item = getEng(A), r = d, p=diag(cor.an$p) %r% 5)
qflextable(tab2)

| item | r | p |
| --- | --- | --- |
| Feeling pain [...] | 0.261 | 0.00000 |
| Muscle pain | 0.184 | 0.00009 |
| Injury/injuries | 0.237 | 0.00000 |
| Feeling tired | -0.087 | 0.06661 |
| Feeling exhausted | 0.064 | 0.17587 |
| Sweating | 0.161 | 0.00064 |
| Feeling uncomfortable | 0.055 | 0.24253 |
| Less time for other activities [...] | -0.148 | 0.00166 |

ct1 <- cor.test(rowSums(A), D$PA)
ct2 <- cor.test(rowSums(BBNS), D$PA)
ct1r <- ct1$estimate %r% 3
ct2r <- ct2$estimate %r% 3
ct1p <- ct1$p.value %r% 5
ct2p <- ct2$p.value %r% 5

The sumscores of acceptance and negative behavioural beliefs had opposite correlations with self-reported PA: the respective *r*s were 0.325 and -0.221 (both *p*s < 0.00001).

Item-level correlations are presented in the following table

ct1 <- corr.test(A, D$PA)
ct2 <- corr.test(BBNS, D$PA)
tab3 <- data.frame(item=getEng(A), r1 = ct1$r %r% 3, p1 =ct1$p %r% 5, r2 = ct2$r %r% 3, p2 = ct2$p %r% 5)
#qflextable(tab3)

###**Table X2.** Negative behavioural beliefs and acceptance of negative consequences: EFA loadings, IRT parameters, and correlations with PA

qft.bbn$r <- tab3$r2
qft.bbn$p <- tab3$p2
qft.acc$r <- tab3$r1
qft.acc$p <- tab3$p1
qft.bbn[1,1] <- qft.acc[1,1] <- "Feeling pain [...]"
ft.bbn <- qflextable(qft.bbn)
ft.acc <- qflextable(qft.acc)

|  | EFA loading | | IRT loading | | discrimination | | location | | r | | p | |  |
| --- | --- | --- | --- | --- | --- | --- | --- | --- | --- | --- | --- | --- | --- |
| Negative behavioural beliefs | | | | | | | | | | | | |  |
| Feeling pain [...] | 0.55 | | 0.57 | | 1.17 | | 1.35 | | -0.166 | | 0.00041 | |  |
| Muscle pain | 0.60 | | 0.63 | | 1.37 | | -0.51 | | -0.143 | | 0.00250 | |  |
| Injury/injuries | 0.40 | | 0.39 | | 0.71 | | 1.77 | | 0.097 | | 0.04034 | |  |
| Feeling tired | 0.64 | | 0.68 | | 1.58 | | 0.14 | | -0.211 | | 0.00001 | |  |
| Feeling exhausted | 0.79 | | 0.84 | | 2.64 | | 0.45 | | -0.237 | | 0.00000 | |  |
| Sweating | 0.34 | | 0.37 | | 0.68 | | -3.15 | | 0.091 | | 0.05567 | |  |
| Feeling uncomfortable | 0.67 | | 0.74 | | 1.86 | | 0.54 | | -0.334 | | 0.00000 | |  |
| Less time for other activities | 0.42 | | 0.48 | | 0.93 | | -0.01 | | -0.143 | | 0.00236 | |  |
| Acceptance of negative consequences of PA | | | | | | | | | | | | | |
| Feeling pain [...] | | 0.47 | | 0.48 | | 0.92 | | 0.96 | | 0.061 | | 0.20059 | |
| Muscle pain | | 0.59 | | 0.65 | | 1.44 | | -1.29 | | 0.254 | | 0.00000 | |
| Injury/injuries | | 0.41 | | 0.41 | | 0.77 | | 0.90 | | 0.176 | | 0.00019 | |
| Feeling tired | | 0.69 | | 0.74 | | 1.85 | | -0.65 | | 0.245 | | 0.00000 | |
| Feeling exhausted | | 0.60 | | 0.64 | | 1.43 | | 0.07 | | 0.147 | | 0.00183 | |
| Sweating | | 0.58 | | 0.68 | | 1.58 | | -1.68 | | 0.196 | | 0.00003 | |
| Feeling uncomfortable | | 0.64 | | 0.68 | | 1.59 | | -0.34 | | 0.117 | | 0.01307 | |
| Less time for other activities [...] | | 0.50 | | 0.53 | | 1.07 | | -0.29 | | 0.431 | | 0.00000 | |

# Injunctive norms

IN <- grabit("IN")
INS <- grabit("IN", Short = TRUE, Order="short", FUN=AVU)
KIN <- subset(K, K$section %in% "IN")
KMO(IN)

ℹ 'x' was not a correlation matrix. Correlations are found from entered raw data.

── Kaiser-Meyer-Olkin criterion (KMO) ──────────────────────────────────────────

✔ The overall KMO value for your data is meritorious.
 These data are probably suitable for factor analysis.

 Overall: 0.846

 For each variable:
 IN1 IN2 IN3 IN4 IN5 IN6 IN7 IN8 IN9 IN10
0.884 0.810 0.825 0.885 0.899 0.806 0.795 0.875 0.762 0.930

BARTLETT(IN)

ℹ 'x' was not a correlation matrix. Correlations are found from entered raw data.

✔ The Bartlett's test of sphericity was significant at an alpha level of .05.
 These data are probably suitable for factor analysis.

 𝜒²(45) = 4348.83, p < .001

k <- kurtosi(IN)
s <- skew(IN)
data.frame(item=getEng(IN), skewness=s %r% 2, kurtosis = k %r% 2, complete.obs=sapply(IN, \(x) sum(!is.na(x)))) |> qflextable()

| item | skewness | kurtosis | complete.obs |
| --- | --- | --- | --- |
| My mother | 0.49 | -1.28 | 381 |
| My father | 0.52 | -1.27 | 328 |
| My brother(s) | 0.86 | -0.65 | 227 |
| My sister(s) | 1.00 | -0.38 | 216 |
| My friend(s) | 0.72 | -0.68 | 360 |
| My colleague(s) | 1.39 | 1.05 | 211 |
| Other (university)students | 1.27 | 0.62 | 254 |
| My partner | 0.34 | -1.29 | 256 |
| My child(ren) | 0.67 | -1.06 | 68 |
| Relatives (other than parents/siblings/children) | 0.89 | -0.57 | 242 |

Parallel analysis of the original items (using pairwise complete observations)

Parallel(IN)

ℹ 'x' was not a correlation matrix. Correlations are found from entered raw data.

Parallel Analysis performed using 100 simulated random data sets
Eigenvalues were found using EFA

Decision rule used: means

── Number of factors to retain according to ────────────────────────────────────

◌ EFA-determined eigenvalues: 4


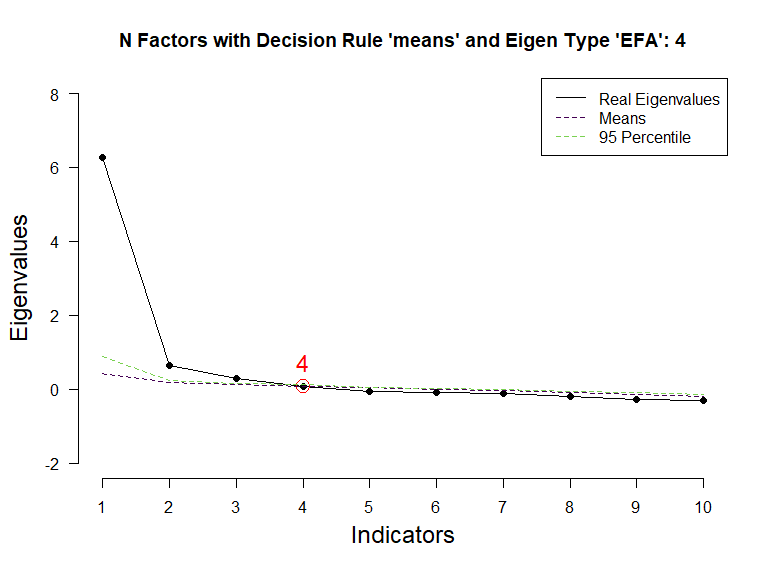


Parallel analysis of the short-version-approximation (using the average of merged item pairs, rounded upwards)

Parallel(INS)

ℹ 'x' was not a correlation matrix. Correlations are found from entered raw data.

Parallel Analysis performed using 100 simulated random data sets
Eigenvalues were found using EFA

Decision rule used: means

── Number of factors to retain according to ────────────────────────────────────

◌ EFA-determined eigenvalues: 3


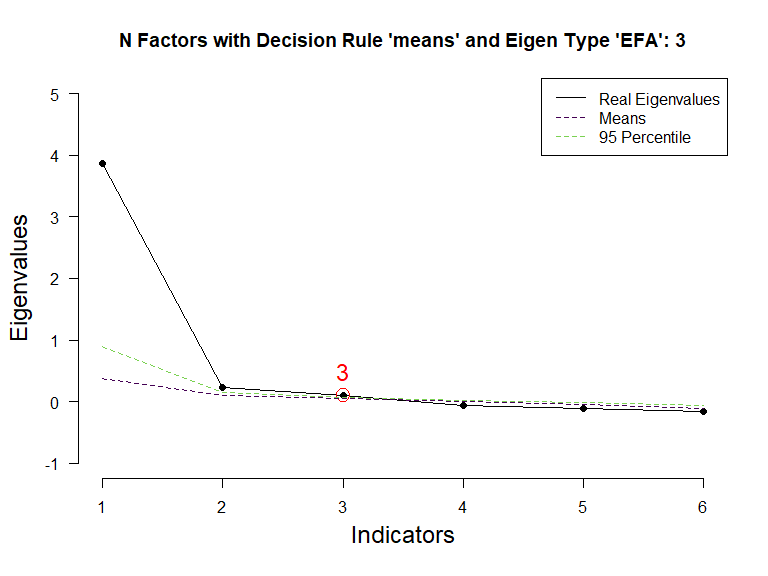


efa.ins <- doefa(INS,1)
exp <- 100*attr(efa.ins, "exp") %r% 3
expn <- 100*attr(efa.ins, "expnext")[2] %r% 3

The first factor explains 64.4% of the variance whereas the second factor adds 7.1%.

ao <- alphaomega(INS) %r% 2

The average correlation among the remaining 8 items was 0.61, with Cronbach alpha of 0.91 and McDonalds’ omega of 0.96.

In the next table, EFA loadings are presented as well as IRT discrimination and generalized location parameters. In the last (rightmost) column, there are standardized factor loadings from IRT (GRM) model. Note that IRT factor loadings correspond well to the EFA ones, except for being a little bit higher in most cases. The generalized location parameter varies from 0.3 (partner) to 0.89 (colleagues and co-students). The items that are best reflective of the general factor are (1) colleagues and co-students and (2) friends, followed by brothers/sisters, and children.

mirtcoef <- coefirt1(mirt.ins)
qft.in <- data.frame(efa.ins, mirtcoef[,-1])
names(qft.in)[1:3] <- c(" ", "EFA loading", "IRT loading")

ft.in <- qflextable(qft.in)
add_header_row(ft.in, values="Injunctive norms", colwidths=5, top=FALSE)

|  | EFA loading | IRT loading | discrimination | location |
| --- | --- | --- | --- | --- |
| Injunctive norms | | | | |
| My mother+My father | 0.67 | 0.74 | 1.87 | 0.51 |
| My brother(s)+My sister(s) | 0.80 | 0.86 | 2.91 | 0.68 |
| My friend(s) | 0.88 | 0.91 | 3.84 | 0.68 |
| My colleague(s)+Other (university)students | 0.82 | 0.94 | 4.69 | 0.89 |
| My partner | 0.79 | 0.80 | 2.28 | 0.30 |
| My child(ren) | 0.84 | 0.86 | 2.91 | 0.56 |

# Descriptive norms

DN <- grabit("DN")
DNS <- grabit("DN", Short = TRUE, Order="short", FUN=AVU)
KMO(DN)

ℹ 'x' was not a correlation matrix. Correlations are found from entered raw data.

── Kaiser-Meyer-Olkin criterion (KMO) ──────────────────────────────────────────

✖ The overall KMO value for your data is miserable.
 These data are hardly suitable for factor analysis.

 Overall: 0.555

 For each variable:
 DN1 DN2 DN3 DN4 DN5 DN6 DN7 DN8 DN9 DN10
0.714 0.349 0.650 0.552 0.523 0.615 0.817 0.377 0.512 0.623

BARTLETT(DN)

ℹ 'x' was not a correlation matrix. Correlations are found from entered raw data.

✔ The Bartlett's test of sphericity was significant at an alpha level of .05.
 These data are probably suitable for factor analysis.

 𝜒²(45) = 1378.97, p < .001

k <- kurtosi(DN)
s <- skew(DN)
data.frame(item=getEng(DN), skewness=s %r% 2, kurtosis = k %r% 2, complete.obs=sapply(DN, \(x) sum(!is.na(x))) ) |> qflextable()

| item | skewness | kurtosis | complete.obs |
| --- | --- | --- | --- |
| My mother | 0.08 | -1.29 | 407 |
| My father | -0.08 | -1.47 | 366 |
| My brother(s) | -0.44 | -1.09 | 265 |
| My sister(s) | -0.17 | -1.11 | 245 |
| My friend(s) | -0.17 | -0.48 | 402 |
| My colleague(s) | -0.05 | -0.69 | 201 |
| Other (university)students | -0.06 | -0.41 | 290 |
| My partner | -0.23 | -1.28 | 256 |
| My child(ren) | -0.97 | -0.35 | 54 |
| Relatives (other than parents/siblings/children) | 0.18 | -0.45 | 242 |

Parallel analysis of the original items (using pairwise complete observations)

Parallel(DN)

ℹ 'x' was not a correlation matrix. Correlations are found from entered raw data.

Parallel Analysis performed using 100 simulated random data sets
Eigenvalues were found using EFA

Decision rule used: means

── Number of factors to retain according to ────────────────────────────────────

◌ EFA-determined eigenvalues: 4


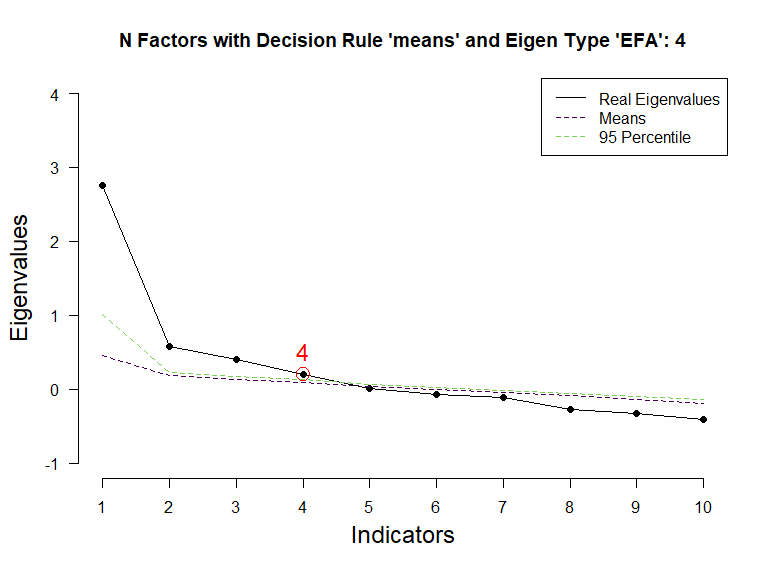


Parallel analysis of the short-version-approximation (using the average of merged item pairs, rounded upwards)

Parallel(DNS)

ℹ 'x' was not a correlation matrix. Correlations are found from entered raw data.

Parallel Analysis performed using 100 simulated random data sets
Eigenvalues were found using EFA

Decision rule used: means

── Number of factors to retain according to ────────────────────────────────────

◌ EFA-determined eigenvalues: 4


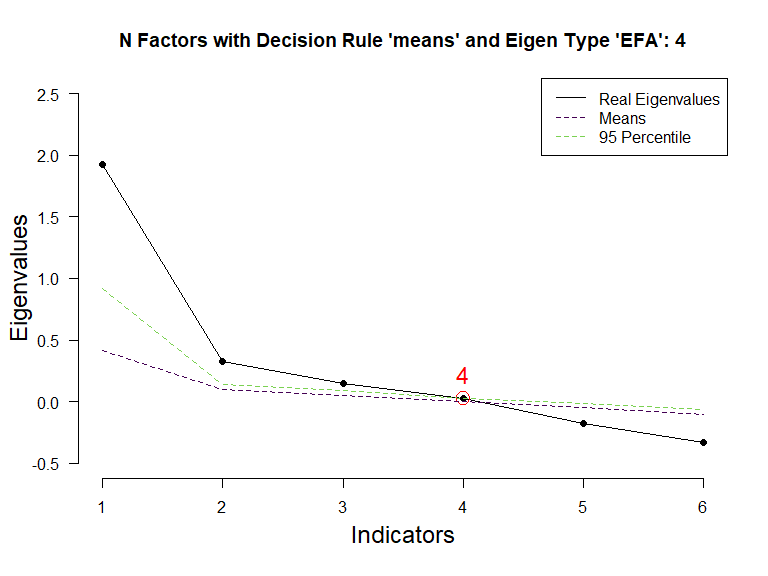


efa.dns <- doefa(DNS,1)
exp <- 100*attr(efa.dns, "exp") %r% 3
expn <- 100*attr(efa.dns, "expnext")[2] %r% 3

The first factor explains 32% of the variance whereas the second factor adds 11.8%.

ao <- alphaomega(DNS) %r% 2

The average correlation among the remaining 8 items was 0.29, with Cronbach alpha of 0.67 and McDonalds’ omega of 0.84.

In the next table, EFA loadings are presented as well as IRT discrimination and generalized location parameters. In the last (rightmost) column, there are standardized factor loadings from IRT (GRM) model. Note that IRT factor loadings correspond well to the EFA ones, except for being a little bit higher in most cases. The generalized location parameter varies from 0.3 (partner) to 0.89 (colleagues and co-students). The items that are best reflective of the general factor are (1) colleagues and co-students and (2) friends, followed by brothers/sisters, and children.

mirtcoef <- coefirt1(mirt.dns)
qft.dn <- data.frame(efa.dns, mirtcoef[,-1])
names(qft.dn)[1:3] <- c(" ", "EFA loading", "IRT loading")

ft.dn <- qflextable(qft.dn)
add_header_row(ft.dn, values="Descriptive norms", colwidths=5, top=FALSE)

|  | EFA loading | IRT loading | discrimination | location |
| --- | --- | --- | --- | --- |
| Descriptive norms | | | | |
| My mother+My father | 0.19 | 0.27 | 0.47 | -0.16 |
| My brother(s)+My sister(s) | 0.48 | 0.44 | 0.83 | -0.67 |
| My friend(s) | 0.63 | 0.67 | 1.52 | -0.50 |
| My colleague(s)+Other (university)students | 0.55 | 0.55 | 1.11 | -0.32 |
| My partner | 0.27 | 0.42 | 0.79 | -0.60 |
| My child(ren) | 0.93 | 0.83 | 2.56 | -0.95 |

# Motivation to comply

MC <- grabit("MC")
MCS <- grabit("MC", Short = TRUE, Order="short", FUN=AVU)
KMO(MC)

ℹ 'x' was not a correlation matrix. Correlations are found from entered raw data.

── Kaiser-Meyer-Olkin criterion (KMO) ──────────────────────────────────────────

✔ The overall KMO value for your data is meritorious.
 These data are probably suitable for factor analysis.

 Overall: 0.801

 For each variable:
 MC1 MC2 MC3 MC4 MC5 MC6 MC7 MC8 MC9 MC10
0.739 0.846 0.855 0.796 0.928 0.867 0.821 0.670 0.676 0.911

BARTLETT(MC)

ℹ 'x' was not a correlation matrix. Correlations are found from entered raw data.

✔ The Bartlett's test of sphericity was significant at an alpha level of .05.
 These data are probably suitable for factor analysis.

 𝜒²(45) = 3833.59, p < .001

k <- kurtosi(MC)
s <- skew(MC)
data.frame(item=getEng(MC), skewness=s %r% 2, kurtosis = k %r% 2, complete.obs=sapply(MC, \(x) sum(!is.na(x))) ) |> qflextable()

| item | skewness | kurtosis | complete.obs |
| --- | --- | --- | --- |
| My mother | -0.25 | -1.21 | 410 |
| My father | 0.00 | -1.33 | 368 |
| My brother(s) | 0.18 | -1.30 | 271 |
| My sister(s) | 0.20 | -1.30 | 245 |
| My friend(s) | -0.24 | -0.88 | 407 |
| My colleague(s) | 0.77 | -0.48 | 241 |
| Other (university)students | 0.75 | -0.55 | 352 |
| My partner | -1.08 | 0.34 | 261 |
| My child(ren) | -0.53 | -1.27 | 62 |
| Relatives (other than parents/siblings/children) | 0.74 | -0.51 | 292 |

Parallel analysis of the original items (using pairwise complete observations)

Parallel(MC)

ℹ 'x' was not a correlation matrix. Correlations are found from entered raw data.

Parallel Analysis performed using 100 simulated random data sets
Eigenvalues were found using EFA

Decision rule used: means

── Number of factors to retain according to ────────────────────────────────────

◌ EFA-determined eigenvalues: 4


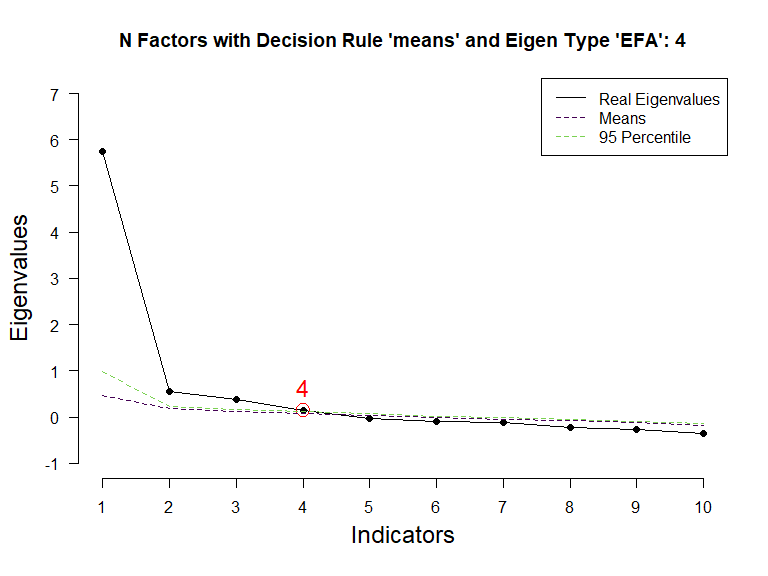


Parallel analysis of the short-version-approximation (using the average of merged item pairs, rounded upwards)

Parallel(MCS)

ℹ 'x' was not a correlation matrix. Correlations are found from entered raw data.

Parallel Analysis performed using 100 simulated random data sets
Eigenvalues were found using EFA

Decision rule used: means

── Number of factors to retain according to ────────────────────────────────────

◌ EFA-determined eigenvalues: 4


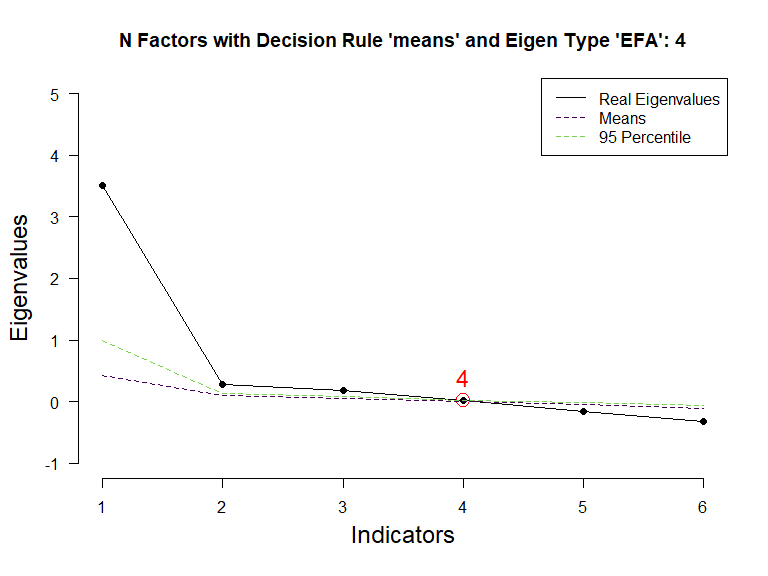


efa.mcs <- suppressWarnings(doefa(MCS,1, max_iter = 1000))
exp <- 100*attr(efa.mcs, "exp") %r% 3
expn <- 100*attr(efa.mcs, "expnext")[2] %r% 3

The first factor explains 58.4% of the variance whereas the second factor adds 30.1%.

ao <- alphaomega(MCS) %r% 2

The average correlation among the remaining 8 items was 0.67, with Cronbach alpha of 0.89 and McDonalds’ omega of 0.96.

In the next table, EFA loadings are presented as well as IRT discrimination and generalized location parameters. In the last (rightmost) column, there are standardized factor loadings from IRT (GRM) model. Note that IRT factor loadings correspond well to the EFA ones, except for being a little bit higher in most cases. The generalized location parameter varies from -0.95 (children) to -0.162 (parents). The items that are best reflective of the general factor are (1) children [but this is not applicable to respondents who do not have children], (2) friends and (3) colleagues.

mirtcoef <- coefirt1(mirt.mcs)
qft.mc <- data.frame(efa.mcs, mirtcoef[,-1])
names(qft.mc)[1:3] <- c(" ", "EFA loading", "IRT loading")
ft.mc<-qflextable(qft.mc)
add_header_row(ft.mc, values="Motivation to comply", colwidths=5, top=FALSE)

|  | EFA loading | IRT loading | discrimination | location |
| --- | --- | --- | --- | --- |
| Motivation to comply | | | | |
| My mother+My father | 0.72 | 0.86 | 2.82 | -0.10 |
| My brother(s)+My sister(s) | 0.83 | 0.88 | 3.17 | 0.15 |
| My friend(s) | 0.78 | 0.83 | 2.52 | -0.17 |
| My colleague(s)+Other (university)students | 0.64 | 0.72 | 1.77 | 0.85 |
| My partner | 0.79 | 0.83 | 2.55 | -0.98 |
| My child(ren) | 0.81 | 0.82 | 2.43 | -0.64 |

# Normative beliefs´

### Table X3. Normative beliefs: EFA loadings and IRT parameters

|  | EFA loading | IRT loading | discrimination | location |
| --- | --- | --- | --- | --- |
| Injunctive norms | | | | |
| My mother+My father | 0.67 | 0.74 | 1.87 | 0.51 |
| My brother(s)+My sister(s) | 0.80 | 0.86 | 2.91 | 0.68 |
| My friend(s) | 0.88 | 0.91 | 3.84 | 0.68 |
| My colleague(s)+Other (university)students | 0.82 | 0.94 | 4.69 | 0.89 |
| My partner | 0.79 | 0.80 | 2.28 | 0.30 |
| My child(ren) | 0.84 | 0.86 | 2.91 | 0.56 |
| Descriptive norms | | | | |
| My mother+My father | 0.19 | 0.27 | 0.47 | -0.16 |
| My brother(s)+My sister(s) | 0.48 | 0.44 | 0.83 | -0.67 |
| My friend(s) | 0.63 | 0.67 | 1.52 | -0.50 |
| My colleague(s)+Other (university)students | 0.55 | 0.55 | 1.11 | -0.32 |
| My partner | 0.27 | 0.42 | 0.79 | -0.60 |
| My child(ren) | 0.93 | 0.83 | 2.56 | -0.95 |
| Motivation to comply | | | | |
| My mother+My father | 0.72 | 0.86 | 2.82 | -0.10 |
| My brother(s)+My sister(s) | 0.83 | 0.88 | 3.17 | 0.15 |
| My friend(s) | 0.78 | 0.83 | 2.52 | -0.17 |
| My colleague(s)+Other (university)students | 0.64 | 0.72 | 1.77 | 0.85 |
| My partner | 0.79 | 0.83 | 2.55 | -0.98 |
| My child(ren) | 0.81 | 0.82 | 2.43 | -0.64 |

# Control beliefs

CB <- grabit("CB")
KMO(CB)

ℹ 'x' was not a correlation matrix. Correlations are found from entered raw data.

── Kaiser-Meyer-Olkin criterion (KMO) ──────────────────────────────────────────

✔ The overall KMO value for your data is meritorious.
 These data are probably suitable for factor analysis.

 Overall: 0.829

 For each variable:
 CB1 CB2 CB3 CB4 CB5 CB6 CB7 CB8 CB9 CB10 CB11 CB12 CB13
0.865 0.920 0.867 0.833 0.889 0.873 0.847 0.605 0.608 0.847 0.761 0.863 0.788
 CB14 CB15 CB16 CB17 CB18 CB19 CB20 CB21 CB22 CB23 CB24
0.790 0.842 0.893 0.900 0.893 0.870 0.520 0.758 0.869 0.899 0.910

BARTLETT(CB)

ℹ 'x' was not a correlation matrix. Correlations are found from entered raw data.

✔ The Bartlett's test of sphericity was significant at an alpha level of .05.
 These data are probably suitable for factor analysis.

 𝜒²(276) = 4366.85, p < .001

k <- kurtosi(CB)
s <- skew(CB)
data.frame(item=getEng(CB), skewness=s %r% 2, kurtosis = k %r% 2 ) |> qflextable()

| item | skewness | kurtosis |
| --- | --- | --- |
| The weather | -0.08 | -1.31 |
| Whether I have time or not | -1.11 | 0.41 |
| How motivated I am | -1.03 | 0.09 |
| My financial situation | 0.82 | -0.58 |
| How I am feeling | -1.00 | 0.30 |
| My energy levels | -0.84 | -0.20 |
| Where I live | 0.14 | -1.36 |
| Whether there is a gym nearby | 0.86 | -0.67 |
| Whether the gym is open or not | 0.84 | -0.89 |
| Whether I have someone to do it with or not [...] | 0.73 | -0.95 |
| My planning skills | -0.68 | -0.59 |
| Whether the environment is safe | -0.32 | -1.17 |
| The amount of obligations that I have | -1.18 | 0.60 |
| How many work or school commitments I have | -1.23 | 0.84 |
| Whether I have a car or not [...] | 1.47 | 0.85 |
| My mood | -0.56 | -0.79 |
| My stress levels | -0.53 | -0.81 |
| Having the necessary equipment | 0.46 | -1.09 |
| Whether people around me are physically active | 0.52 | -0.94 |
| My children | 0.41 | -1.43 |
| The Covid-19 situation | 0.45 | -1.24 |
| My health | -1.16 | 0.88 |
| How fast I see the results | 0.40 | -0.98 |
| My other goals | -0.40 | -0.78 |

CBS <- grabit("CB", Order="short", Short=TRUE, FUN=AVU)
eng <- attr(CBS, "Eng")[1:14]
CBS <- structure(CBS[,1:14], Eng=eng)

KMO(CBS)

ℹ 'x' was not a correlation matrix. Correlations are found from entered raw data.

── Kaiser-Meyer-Olkin criterion (KMO) ──────────────────────────────────────────

✔ The overall KMO value for your data is middling.
 These data are probably suitable for factor analysis.

 Overall: 0.749

 For each variable:
 CB1 CB5 CB22 CB11 CB23 CB2 CB8 CB9 CB15 CB18 CB4 CB7 CB12
0.786 0.777 0.741 0.757 0.834 0.764 0.609 0.604 0.843 0.863 0.861 0.827 0.788
 CB10
0.813

BARTLETT(CBS)

ℹ 'x' was not a correlation matrix. Correlations are found from entered raw data.

✔ The Bartlett's test of sphericity was significant at an alpha level of .05.
 These data are probably suitable for factor analysis.

 𝜒²(91) = 1471.66, p < .001

ao <- alphaomega(CBS) %r% 2
cm <- cor(CBS, use="com")
cm <- cm[lower.tri(cm)]

The shortened scale of CBs cannot be treated as unidimensional, even if the internal consistency indicators may be numerically satisfactory (alpha = 0.75, omega = 0.81, average interitem correlation = 0.18).

Parallel(CBS)

ℹ 'x' was not a correlation matrix. Correlations are found from entered raw data.

Parallel Analysis performed using 100 simulated random data sets
Eigenvalues were found using EFA

Decision rule used: means

── Number of factors to retain according to ────────────────────────────────────

◌ EFA-determined eigenvalues: 4


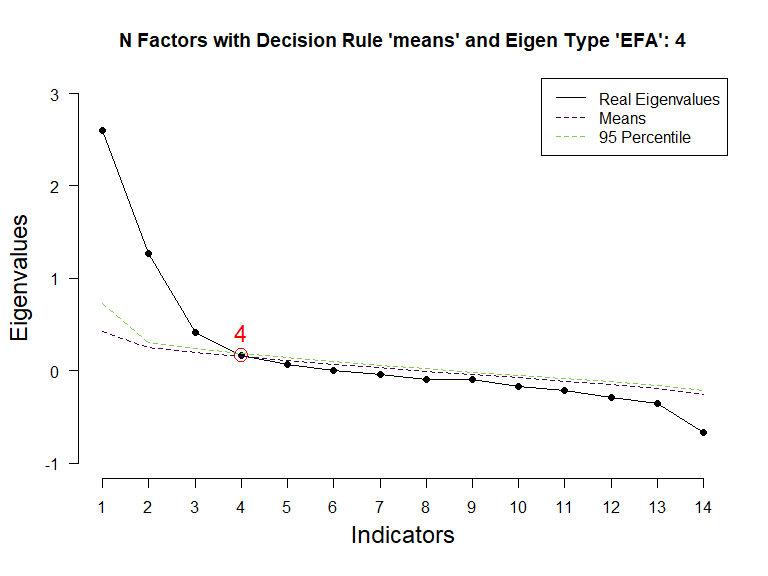


Parallel analysis suggests 4 factors; according to eyeballing the scree plot, one might also consider a 3-factor solution.

Three-factor solution (promax rotation): loadings and factor correlations

EFA(CBS, 3, rotation="promax") -> efa3

ℹ 'x' was not a correlation matrix. Correlations are found from entered raw data.

EFA(CBS, 4, rotation="promax") -> efa4

ℹ 'x' was not a correlation matrix. Correlations are found from entered raw data.

lim <- 0.4
data.frame(" "=getEng(CBS), efa3$rot_loadings |> unclass() |> round(3), check.names=FALSE) |> qflextable() |> bold(i= ~ F1> lim, j=2) |> bold(i=~ F2>lim, j=3) |> bold(i=~ F3>lim, j=4)

|  | F1 | F2 | F3 |
| --- | --- | --- | --- |
| The weather | -0.202 | 0.331 | 0.297 |
| How I am feeling | 0.040 | **0.688** | -0.013 |
| My health | 0.026 | **0.582** | -0.172 |
| My planning skills | 0.107 | 0.378 | -0.048 |
| How fast I see the results | -0.105 | 0.068 | **0.556** |
| Whether I have time or not | 0.022 | **0.421** | 0.068 |
| Whether there is a gym nearby | **0.900** | 0.049 | -0.028 |
| Whether the gym is open or not | **0.938** | -0.029 | -0.027 |
| Whether I have a car or not [...] | 0.159 | -0.195 | **0.499** |
| Having the necessary equipment | 0.204 | 0.053 | **0.481** |
| My financial situation | 0.288 | 0.086 | 0.338 |
| Where I live | 0.091 | 0.103 | **0.441** |
| Whether the environment is safe | -0.070 | **0.496** | 0.020 |
| Whether I have someone to do it with or not [...] | -0.132 | -0.102 | **0.557** |

data.frame(efa3$Phi)|> round(3) |> qflextable()

| X1 | X2 | X3 |
| --- | --- | --- |
| 1.000 | 0.050 | 0.319 |
| 0.050 | 1.000 | 0.563 |
| 0.319 | 0.563 | 1.000 |

Four-factor solution (promax rotation): loadings and factor correlations

rolo <- rbind(efa4$rot_loadings, efa4$vars_accounted_rot[2,])[, c(1, 4,2,3)]

xt4 <- data.frame(" "=c(getEng(CBS), "% var accounted"), round(rolo,2), check.names=FALSE) |> qflextable() |> bold(i= ~ F1> lim, j=2) |> bold(i=~ F2>lim, j=3) |> bold(i=~ F3>lim, j=4) |> bold(i=~F4>lim, j=5)

### Table X4. Control beliefs: EFA loadings of the shortened version

|  | F1 | F2 | F3 | F4 |
| --- | --- | --- | --- | --- |
| The weather | -0.14 | -0.08 | 0.18 | **0.56** |
| How I am feeling | 0.06 | 0.33 | -0.05 | **0.44** |
| My health | -0.02 | **0.52** | -0.12 | 0.08 |
| My planning skills | 0.03 | **0.54** | 0.03 | -0.15 |
| How fast I see the results | -0.13 | 0.04 | **0.54** | 0.08 |
| Whether I have time or not | 0.12 | -0.02 | -0.08 | **0.61** |
| Whether there is a gym nearby | **0.92** | -0.02 | -0.04 | 0.07 |
| Whether the gym is open or not | **0.93** | -0.03 | -0.01 | -0.03 |
| Whether I have a car or not [...] | 0.12 | -0.08 | **0.51** | -0.12 |
| Having the necessary equipment | 0.13 | 0.18 | **0.55** | -0.13 |
| My financial situation | 0.27 | 0.04 | 0.33 | 0.08 |
| Where I live | 0.11 | -0.04 | 0.39 | 0.22 |
| Whether the environment is safe | -0.12 | **0.49** | 0.06 | 0.05 |
| Whether I have someone to do it with or not [...] | -0.14 | -0.10 | **0.52** | 0.05 |
| % var accounted | 0.14 | 0.07 | 0.11 | 0.08 |

data.frame(efa4$Phi)|> round(3) |> qflextable()

| X1 | X2 | X3 | X4 |
| --- | --- | --- | --- |
| 1.000 | 0.364 | -0.010 | 0.125 |
| 0.364 | 1.000 | 0.492 | 0.457 |
| -0.010 | 0.492 | 1.000 | 0.620 |
| 0.125 | 0.457 | 0.620 | 1.000 |

FS.CBS <- FACTOR_SCORES(CBS, efa4)$scores
efa4_o <- EFA(CB, 4, rotation="promax")

ℹ 'x' was not a correlation matrix. Correlations are found from entered raw data.

FS.CB <- FACTOR_SCORES(CB, efa4_o)$scores

smirt.cbs <- summary(mirt.cbs, rotate = "promax", verbose=FALSE)
irtldngs <- smirt.cbs$rotF |> unclass() |> round(RT) |> data.frame()

irtldngs <- data.frame(" " = getEng(CBS), irtldngs)
qflextable(irtldngs) |> bold(i= ~ F1> lim, j=2) |> bold(i=~ F2>lim, j=3) |> bold(i=~ F3>lim, j=4) |> bold(i=~F4>lim, j=5)

| X. | F1 | F2 | F3 | F4 |
| --- | --- | --- | --- | --- |
| The weather | 0.40 | 0.18 | -0.28 | 0.16 |
| How I am feeling | -0.01 | **0.68** | 0.07 | 0.15 |
| My health | -0.14 | **0.74** | 0.07 | -0.10 |
| My planning skills | -0.03 | **0.46** | 0.13 | -0.04 |
| How fast I see the results | **0.63** | 0.02 | -0.17 | 0.04 |
| Whether I have time or not | -0.04 | 0.07 | 0.08 | **0.97** |
| Whether there is a gym nearby | 0.10 | 0.04 | **0.90** | 0.07 |
| Whether the gym is open or not | 0.06 | 0.04 | **0.98** | 0.01 |
| Whether I have a car or not [...] | **0.62** | -0.24 | 0.19 | -0.02 |
| Having the necessary equipment | **0.62** | 0.00 | 0.19 | -0.05 |
| My financial situation | **0.45** | 0.10 | 0.26 | -0.06 |
| Where I live | **0.66** | -0.03 | -0.02 | -0.01 |
| Whether the environment is safe | 0.29 | 0.38 | -0.22 | -0.07 |
| Whether I have someone to do it with or not [...] | **0.41** | 0.05 | 0.03 | -0.03 |

Factor correlations

smirt.cbs$fcor |> as.data.frame() |> round(RT) |> qflextable()

| F1 | F2 | F3 | F4 |
| --- | --- | --- | --- |
| 1.00 | 0.53 | 0.34 | 0.30 |
| 0.53 | 1.00 | -0.09 | 0.43 |
| 0.34 | -0.09 | 1.00 | -0.06 |
| 0.30 | 0.43 | -0.06 | 1.00 |

# Prediction of physical activity

rsq <- \(x) round(summary(x)$r.squared*100,2)
mbbp1 <- cor.test(D$PA , rowSums(BBP))
mbbp2 <- cor.test(D$PA , rowSums(BBPS))
mbbp3 <- cor.test(D$PA , rowSums(BBPS2))
mbbn1 <- cor.test(D$PA , rowSums(BBN))
mbbn2 <- cor.test(D$PA , rowSums(BBNS))
ma <- cor.test(D$PA , rowSums(A))
min <- cor.test(D$PA, rowMeans(IN, na.rm=TRUE))
mins <- cor.test(D$PA, rowMeans(INS, na.rm=TRUE))
mdn <- cor.test(D$PA, rowMeans(DN, na.rm=TRUE))
mdns <- cor.test(D$PA, rowMeans(DNS, na.rm=TRUE))
mmc <- cor.test(D$PA, rowMeans(MC, na.rm=TRUE))
mmcs <- cor.test(D$PA, rowMeans(MCS, na.rm=TRUE))
lmcb <- lm(D$PA ~ FS.CB)
lmcbs <- lm(D$PA ~ FS.CBS)

muds1 <- list(mbbp1, mbbp1, mbbn1, ma, min, mdn, mmc, lmcb )
muds2 <- list(mbbp2, mbbp3, mbbn2, ma, mins, mdns, mmcs, lmcbs )

getit <- function(x) {
 if("htest" %in% class(x)) c(x$estimate, x$p.value) else c(summary(x)$r.squared^.5, anova(x)$`Pr(>F)`[1])
}

res <- data.frame(cbind(t(sapply(muds1, getit)), t(sapply(muds2, getit))))
res <- data.frame(" " = c("Positive BB", "Positive BB(2)", "Negative BB", "Acceptance", "Injunctive norms", "Descriptive norms", "Motivation to comply", "Control beliefs"), res)
names(res) <- c("var", "r", "p", "r2", "p2")
res$r <- round(res$r, 2)
res$r2 <- round(res$r2, 2)
res$p <- round(res$p, 5)
res$p2 <- round(res$p2, 5)
res$r[2] <- NA
res$p[2] <- NA

### Table X5. Correlations with PA

| var | r | p | r2 | p2 |
| --- | --- | --- | --- | --- |
| Positive BB | 0.29 | 0.00000 | 0.26 | 0.00000 |
| Positive BB(2) |  |  | 0.29 | 0.00000 |
| Negative BB | -0.18 | 0.00010 | -0.22 | 0.00000 |
| Acceptance | 0.32 | 0.00000 | 0.32 | 0.00000 |
| Injunctive norms | -0.40 | 0.00000 | -0.39 | 0.00000 |
| Descriptive norms | 0.11 | 0.02124 | 0.15 | 0.00197 |
| Motivation to comply | 0.00 | 0.97826 | 0.00 | 0.94239 |
| Control beliefs | 0.48 | 0.00490 | 0.48 | 0.00000 |

lmbig <- lm(scale(D$PA) ~ scale(rowSums(BBPS2)) + scale(rowSums(BBNS)) + scale(rowSums(A)) + scale(rowMeans(INS, na.rm=T)) + scale(rowMeans(DNS, na.rm=T)) + scale(rowMeans(MCS, na.rm=T)) + apply(FS.CBS,2, scale))
res2 <- coef(summary(lmbig))[-1,]
res2 <- data.frame(var=c(res$var[-2], "F3", "F4", "F2"), res2)
res2$var[7:10] <- paste0("Control beliefs F", c(1,3,4,2))
names(res2) <- c("var", "b", "se(b)", "t", "p")
res2[, c(2,3,4)] <- round(res2[, c(2,3,4)], 2)
res2$p <- round(res2$p,4)

### Table X6. Multiple regression analysis, predicting PA from TPB+acceptance

| var | b | se(b) | t | p |
| --- | --- | --- | --- | --- |
| Positive BB | 0.08 | 0.05 | 1.46 | 0.1453 |
| Negative BB | -0.07 | 0.05 | -1.52 | 0.1291 |
| Acceptance | 0.18 | 0.05 | 3.81 | 0.0002 |
| Injunctive norms | -0.22 | 0.05 | -4.53 | 0.0000 |
| Descriptive norms | 0.15 | 0.05 | 3.21 | 0.0015 |
| Motivation to comply | 0.02 | 0.05 | 0.48 | 0.6318 |
| Control beliefs F1 | 0.08 | 0.05 | 1.49 | 0.1359 |
| Control beliefs F3 | -0.04 | 0.07 | -0.59 | 0.5544 |
| Control beliefs F4 | -0.32 | 0.08 | -3.80 | 0.0002 |
| Control beliefs F2 | 0.11 | 0.08 | 1.37 | 0.1704 |

R^2^ = 0.351, F `(10,330) =17.88, p< 2.22e-16.

lmbig2 <- lm(scale(D$PA) ~ scale(rowSums(BBP)) + scale(rowSums(BBN)) + scale(rowSums(A)) + scale(rowMeans(IN, na.rm=T)) + scale(rowMeans(DN, na.rm=T)) + scale(rowMeans(MC, na.rm=T)) + apply(FS.CB,2, scale))
